# A review of gorgonian coral species (Cnidaria, Octocorallia, Alcyonacea) held in the Santa Barbara Museum of Natural History research collection: focus on species from Scleraxonia, Holaxonia, Calcaxonia – Part III: Suborder Holaxonia continued, and suborder Calcaxonia

**DOI:** 10.3897/zookeys.860.34317

**Published:** 2019-07-04

**Authors:** Elizabeth Anne Horvath

**Affiliations:** 1 Westmont College, 955 La Paz Road, Santa Barbara, California 93108 USA Westmont College Santa Barbara United States of America; 2 Invertebrate Laboratory, Santa Barbara Museum of Natural History, 2559 Puesta del Sol Road, Santa Barbara, California 93105 USA Santa Barbara Museum of Natural History Santa Barbara United States of America

**Keywords:** Allan Hancock Foundation (AHF) – ’Velero’ Expeditions, colony form, deep-water gorgonians, geographical/ecological variation, museum collection, sclerite morphology, soft corals, *
Swiftia
*, *
Thesea
*, “thread-like” forms

## Abstract

Alcyonacean (Gorgonian) coral species from Holaxonia (not previously reviewed in this three-part work), family Plexauridae, as well as species in Calcaxonia were reviewed. Specimens examined were collected from the California Bight and adjacent areas, many now held in the research collection of the Santa Barbara Museum of Natural History (SBMNH). The collection has incorporated numerous specimens collected by the Allan Hancock Foundation (AHF) ‘Velero’ Expeditions of 1931–1941 and 1948–1985. This historic collection displays an emphasis on species belonging to the Holaxonia, particularly gorgoniids and plexaurids. This third part of the larger work presented a thorough, in-depth discussion of at least one genus (*Swiftia* Duchassaing & Michelotti, 1864) in the Plexauridae found within the California Bight that has generated some taxonomic confusion; in that discussion are comments on other genera (such as *Psammogorgia* Verrill, 1868a, to which several species had been previously ascribed). The discussion of *Swiftia* includes description of a morphological trend (encompassing colony form, color and sclerite form), likely influenced by geography and ecology, not noted or discussed previously. Additionally, a preliminary discussion of the genus (*Thesea* Duchassaing & Michelotti, 1860) was presented; this genus, both historically and currently, has not been fully examined in California waters. Finally, a short review was given for the few species of Calcaxonia represented in the SBMNH research collection. This paper, Part III of the full review, continued and concludes the systematic examination of species represented in the SBMNH research collection begun in Part I, continued in Part II, focusing on all species of gorgonian coral held in the SBMNH research collection, known to currently inhabit the California Bight and adjacent areas.

## Introduction

Species in the family Plexauridae (Holaxonia) are well represented in the Santa Barbara Museum of Natural History’s (SBMNH) research collection. While a sufficient number of specimens, representing the genera *Swiftia* Duchassaing & Michelotti, 1864 and *Thesea* Duchassaing & Michelotti, 1860, were already present, with the acquisition of gorgonian materials from the Allan Hancock Foundation (AHF) ‘Velero’ Expeditions, the collection was enhanced. Studies were undertaken to identify (or to correct identification of) not only specimens already in the collection, but the many specimens collected during the ‘Velero’ years of operation that were being added to the collection. Additionally, the staff of a number of National Oceanographic and Atmospheric Administration (NOAA) facilities throughout the country generously provided additional material, representing the genus *Swiftia*, for study. In that multiple-year study, the key morphological features were clarified, and an interesting south to north morphological trend, likely predicated on geographic and ecological parameters was defined. Additionally, the SBMNH has an extensive holding of specimens in the genus *Thesea* collected off California; this genus requires further work. A preliminary discussion of the situation as seen in California waters is presented. Holdings in the genus *Thesea* have been significantly enhanced by collection and survey work done by both the Los Angeles County and Orange County Sanitation Districts (LACSD, OCSD, respectively). Finally, while representation of the Calcaxonia and its members is not extensive, the SBMNH research collection does include specimens from both the families Primnoidae and Isididae; these are briefly described and discussed, concluding this review of the SBMNH alcyonacean gorgonian research collection, begun with Parts I and II.

## Materials and methods

A large majority of the specimens examined in this work (housed currently as part of the Santa Barbara Museum of Natural History’s permanent research collection, invertebrate laboratory), were collected over a period of years dating from the 1930s to the present, in either dry or wet condition. A large percentage of these specimens came to the SBMNH through a diverse 10,000-lot cnidarian collection, a portion of the Allan Hancock Foundation (AHF) collection built upon the historic ‘Velero’ expeditions of 1931–1941 and 1948–1985. To assist with the identification of the SBMNH specimens, examinations of specimens of known species from or collected in the Bight were performed on material found in the collections of the National Museum of Natural History, Smithsonian (**USNM** = NMNH), the California Academy of Sciences, San Francisco (**CAS**), the Los Angeles County Museum of Natural History (**LACoMNH**), Scripps Institute of Oceanography (**SIO**), the Monterey Bay Aquarium Research Institute (**MBARI**), Moss Landing Marine Laboratories (**MLML**) and the small museum which is a part of the Cabrillo Marine Aquarium in San Pedro, California (**CMA**) (see Appendix 3: List of material examined). These were compared to SBMNH specimens, informing the identification of species represented in the SBMNH collection. Additionally, several National Oceanographic and Atmospheric Administration (NOAA) offices throughout the country provided further material for study; the genus *Swiftia* is predominantly represented in that material.

All specimens were examined for gross colony morphology; more importantly, examination of the calcareous sclerites, present in different parts of the colony, was conducted for nearly all specimens. The standard method for sclerite extraction (tissue sample in common household bleach) was performed, and light microscopy via a compound Olympus (CH) microscope, was used initially to determine the genus to which a specimen belonged. Scanning Electron Microscopy (SEM) of the sclerites was then undertaken. All samples were coated with gold, using a Cressington Sputter Coater Unit, 108auto. Samples were examined, and digital images taken using a Zeiss Scanning Electron Microscope EVO 40, at 10 kV. This third part covers some eighteen species, spread over roughly eleven genera. A summative overview of species housed in the SBMNH research collection is included below.

**Table:** Part III: Collective specimen and species data

# of specimens analyzed with sclerite preparations	~275
# of specimens examined without sclerite preparation	5–10
Breakdown of specimens examined:	
# of specimens analyzed from SBMNH collection	~125
# of specimens analyzed from USNM-Smithsonian	50
# of specimens analyzed from CAS	3–5
# of specimens analyzed from other institutions (primarily NOAA)	~97–100
Breakdown of species examined:	
Total # of species that underwent sclerite observations	~18
# of new species described	0
# of species examined from the SBMNH collection	~9
# of species examined from USNM-Smithsonian	13
# of species examined from CAS	3
# of species examined from other sources (primarily NOAA and Scripps)	10
# of species shown in Figures (colony)	9
# of species shown in Figures (either light microscopy and/or SEM of sclerites)	9

**Table:** Species covered in this part:

	SBMNH	Other institutions	Colony figure	Sclerite figure
* Swiftia kofoidi *	Yes	Yes	Yes	Yes
* Swiftia pacifica *	Yes	Yes	Yes	Yes
* Swiftia simplex *	Yes	Yes	Yes	Yes
* Swiftia spauldingi *	No	Yes	Yes	Yes
* Swiftia torreyi *	No	Yes	No	No
* Swiftia pusilla *	No	Yes	No	No
*Thesea* spp.	Yes	Yes	Yes	Yes
* Thesea variabilis *	No	Yes	No	No
* Callogorgia kinoshitai *	Yes	Yes	Yes	Yes
* Parastenella pacifica *	Yes	Yes	Yes	Yes
* Parastenella ramosa *	No	Yes	No	No
* Pulmarella longispina *	Yes	Yes	Yes	Yes
* Primnoa pacifica *	No	Yes	No	No
*Narella* sp.	No	Yes	No	No
*Acanella* sp.	No	Yes	No	No
*Isidella* sp.	Yes	Yes	No	No
*Keratoisis* sp.	Yes	Yes	Yes	Yes
*Lepidisis* sp.	No	Yes	No	No

This information for Part III (examination of colony morphology and sclerites) is a summation of more detailed information found in the Appendix 3: List of material examined – Part III. It is evident from this summative overview that the SBMNH research collection houses species from the holaxonian family Plexauridae, specifically the genera *Swiftia* and *Thesea*, but lacks a comprehensive collection of calcaxonian species present in the California Bight.

## Systematic accounts

(Classification used throughout this paper conforms to that of Bayer 1981c)

### Diagnosis of Holaxonia Studer, 1887

With distinct central axis composed of horny material alone or of horny material more or less heavily permeated with calcareous substance, continuous or with alternating horny and calcareous joints. In center of axis is a relatively narrow, largely hollow, tubular space partitioned into series of small chambers, referred to as the cross-chambered central chord. Calcareous material of the peripheral zone of axis is in nonscleritic form (single exception in Keroeididae).

#### Key to the genus *Swiftia* and the California “red whips” (many mistaken for *Swiftia* or other “red whip” genera and species)

**Table:** 

1	Coenenchyme of moderate thickness, containing spindles and warted clubs; clubs are coarse, irregular thorn-clubs, usually pink or red (no purple or lavender sclerites), uncommonly yellow or white; color of colony a bright red to orange-red	**Genus *Psammogorgia*** (only briefly discussed for comparison purposes; there are no species of this genus represented in the CA Bight)
–	Coenenchyme of moderate thickness, containing spindles and/or capstans, but no warted clubs; color red, orange, pinkish-red, reddish-purple (genera including those of *Leptogorgia, Chromoplexaura, Swiftia*; first two genera listed, Part II)	**2**
2	Colonies primarily single, whip-like; or few, slender branches, loose, long; if multiple branches, only loosely flabellate, perhaps irregularly dichotomous. Not reticulated; color orange-red, coral, reddish-pink or red	**3**
–	Colonies can be sparsely to rather densely branched, opposite/alternate or pinnate; possibly reticulated. Polyp mounds prominent, conical, creating branch profile such that zig-zag pattern seen. Colors orange-red, salmon, brick-red to deep red or a deep reddish-purple	**6**
3	Acute or subacute spindles with warts forming rings/disks; small, thin, minimally warted anthocodial rods (pale orange); see Part II	*** Leptogorgia chilensis ***
–	Sclerites as spindles, capstans; may have conspicuous fingerbiscuit rods	**4**
4	Polyp mounds flush, or very slightly raised as low mounds, or more obvious rounded protuberances. If colonies branched, mostly dichotomous	**5**
–	Polyp-mounds (? calyces) broad conical to cylindrical; with (?) collaret; anthocodia exsert; polyp color generally pale pink to nearly white	**Unknown Red Whip** (transitional in appearance between *L.chilensis* and *C.marki*, see Part II; regional endemic?)
5	Color of colony orange, orange-red or red, with heavy branches, mostly lateral; may appear dichotomous. Anthocodia white; spindles as large double-dunce caps; fingerbiscuit rods absent; shallow to moderately deep water (see Part II)	*** Chromoplexaura marki ***
–	Color of colony red/orange, salmon to coral, with moderately heavy branches, irregularly dichotomous. Anthocodia white; polyp mounds rounded protuberances, closely spaced; small, short spindles and double-spindles; fingerbiscuit rods present, heavily warted	** Swiftia cf. spauldingi **
–	Color of colony pinkish red (brick red), few branches round, of moderate uniform diameter. Anthocodia red; polyp mounds not prominently raised, generally flush; large spindles long, thin; pronounced fingerbiscuit rods orange; found in deep water	** Swiftia cf. simplex **
6	Color of colony bright orange, polyps white; moderately branched in pinnate pattern. Polyps widely spaced, as large, prominent, conical mounds, usually in irregular biserial rows; anthocodia well-developed, often exsert. Spindles short, thorny; those longer, slender; few to no anthocodial bars (fingerbiscuit rods)	*** Swiftia kofoidi ***
–	Color of anthocodia (well-developed, often exsert) and polyps deep red to deep gray/greenish-red, coenenchyme deep red. Moderately branched; in general, an opposite/alternate pattern. Polyp mounds lateral, prominent, rounded, moderately to closely spaced. Sclerites symmetrical capstans and spindles; anthocodial bars (fingerbiscuit rods) clearly seen, large, warted	*** Swiftia pacifica ***
–	Color of colony deep reddish-purple; branches usually opposite from main stem, commonly anastomosing. Polyp mounds truncated, tubular cones, scattered on all sides of branches, closely spaced. Spindles of moderate length, sometimes slightly curved, deep reddish-purple; anthocodial fingerbiscuit rods orange, very conspicuous	*** Swiftia torreyi ***

#### List of species of Holaxonia Studer, 1887

Class Anthozoa

Subclass Octocorallia Haeckel, 1866

Order Alcyonacea Lamouroux, 1816

Holaxonia Studer, 1887

Family Plexauridae Gray, 1859

Swiftiacf.kofoidi (Nutting, 1909)

*Swiftiapacifica* (Nutting, 1912)

*Swiftiasimplex* (Nutting, 1909)

Swiftiacf.spauldingi (Nutting, 1909)

*Swiftiatorreyi* (Nutting, 1909)

*Swiftiapusilla* (Nutting, 1909)

*Thesea* spp.

#### Descriptions of species of Holaxonia Studer, 1887

##### Genus Primnoidae

Taxon classificationAnimaliaAlcyonaceaPrimnoidae

Duchassaing & Michelotti, 1864

Gorgonia (part) Valenciennes, 1855: 12.
Swiftia Duchassaing & Michelotti, 1864: 13. Kükenthal 1924: 236. Deichmann 1936: 185–186. Bayer 1956: F206. Grasshoff 1977: 161. Muzik 1979: 167. Bayer 1981: 945. Breedy et al. 2015: 329.
Stenogorgia Verrill, 1883: 29 [= Swiftia, des. by Deichmann 1936: 186]. Grieg 1887: 5, 18. Studer (and Wright) 1887: 64. Studer 1901: 51. Nutting 1909: 723; 1910c: 6. Jungersen 1917: 1186. Bielschowsky 1918: 45. Kükenthal 1924: 347 (Stenogorgia synonymy).
Platycaulos Wright & Studer, 1889: 61, 146–147. Nutting 1912: 94. Bayer 1981: 945.
Callistephanus Wright & Studer, 1889: 62, 148. Nutting 1912: 96. Bayer 1981: 945.
Allogorgia , Verrill, 1928: 8.
Thesea (pars) Verrill, 1869: 428.
Filigorgia Stiasny, 1937: 307.

###### Type species.

*Gorgoniaexserta* Ellis & Solander, 1786: 87 (non *Theseaexserta* Duchassaing & Michelotti, 1860); [= *Stenogorgia* Verrill, 1883].

###### Diagnosis.

Colonies chiefly in one plane, with lax branching (dichotomous or pinnate-like); branches/branchlets tend to curve upwards; in some species, anastomoses possible (fan-like); in others, minimal branching or none. Polyps widely scattered, or crowded; often lateral or biserial, forming prominent conical or cylindrical mounds; on tips of branchlets, two polyps always opposed; conical anthostele seldom retracted; generally, polyps retractile. Anthocodiae commonly tall, exsert. Coenenchyme thin to moderate, somewhat rough/granular, outer layer filled not only with spinous rods or spindles, but with capstans having warts more or less conspicuously modified as double disks; some capstans quite foliate; inner layer mostly restricted to areas between longitudinal canals, containing only small capstans. Mound margins, base of tentacles, with numerous rows of conspicuous, stout spindles as bar-like rods, characteristic for species in the genus (fingerbiscuit shaped; see Bayer et al. 1983, pp 72–73, pl 19, figs 184–185). Axis is horny, flexible, somewhat flattened. Colony colors generally red, red-orange, pink or white.

###### Etymology.

Deichmann (1936) stated that the definition of the genus *Swiftia* corresponded exactly with *Stenogorgia* Verrill, 1883; the problem discussed there stemmed from a misinterpretation of *G.exserta* Ellis & Solander, 1786 by Verrill (also by Kükenthal 1924). See remarks, following.

###### Remarks.

**Map A1.**
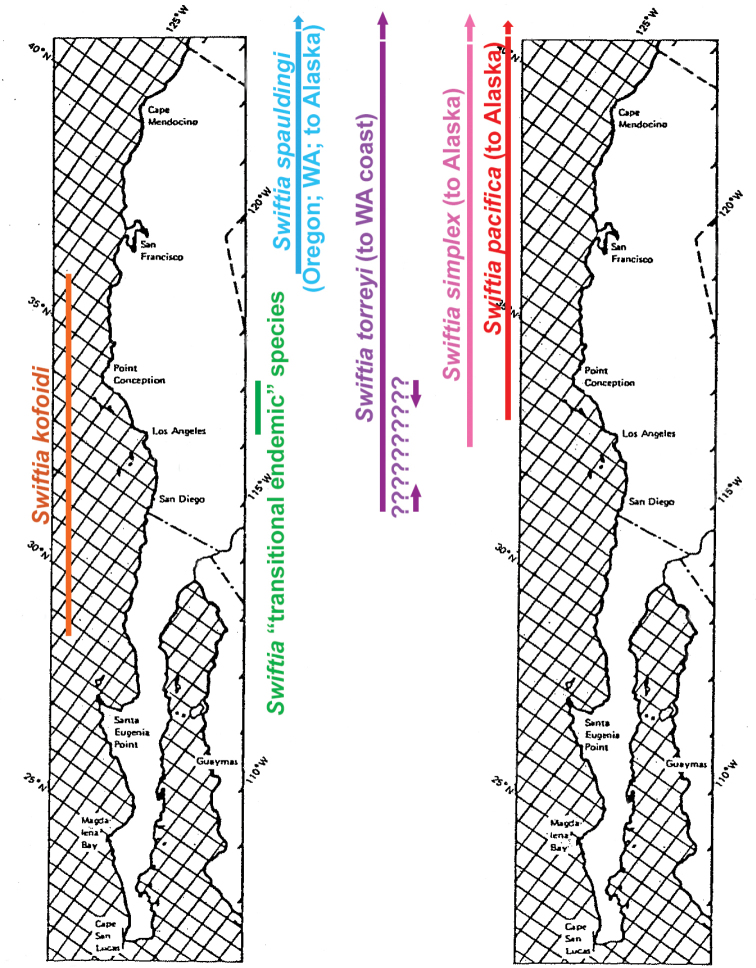
Distribution of species in genus *Swiftia* seen in, or immediately adjacent to, the California Bight. Range extensions are not definitive, but illustrative of the geographic distribution of specimens housed in SBMNH collection.

The stout, anthocodial rods (seen at mound margins and bases of tentacles), are definitive for this genus. Examinations of multiple specimens (several different species) within this genus usually revealed the appearance of these rods; when present, looking much like the fingerbiscuit sclerite form shown in Bayer et al. (1983) for the genera *Clavularia* Blainville, 1830 and *Ptilosarcus* Verrill, 1865 (neither of these gorgonian genera), where sclerites are described as minute, flattened rods (rods here have a bit of depth). Further examinations (multiple species) revealed that some individual colonies of species in the genus did not have these conspicuous rod forms (having only spindles and capstans). Other colonies displayed spinous spindles and/or capstans and anthocodial rods; some few species had only the fingerbiscuit rods, numerous throughout all tissue structures. The rod form is not always easy to obtain in a sclerite array; some specimens without rods may actually have them, but they may be quite small, not very numerous and very widely scattered. A trend observed is that colonies further north in the Pacific (Alaska) have very obvious rods, while specimens of some of the same species collected in California (specifically central and southern California) may have rods, but infrequently. By way of comparison, in several species from the genus examined from waters in/near New Zealand, some had only rods, and no other form of sclerite. It appeared that colder, temperate to subpolar species had the rods (to the exclusion of all others) but species from warmer, albeit temperate water, tended to display a minimal number or complete absence of rods. Examination of many more specimens, collected in both hemispheres from poles to equator, could reveal further insight into the appearance of this key sclerite form. To further clarify questions surrounding location ranges for each of the *Swiftia* species discussed, Appendix 2: Map A1 shows the distributional range of each and Appendix 1: Table A1 shows key features used to distinguish one species from another.

Regarding use of the generic name *Swiftia*, Muzik (1979) stated: “(t)o preserve the generic name Swiftia a petition to” the International Commission on Zoological Nomenclature “(ICZN) must be made;” uncertain as to whether this was ever done. “For a full explanation, see Challenger Reports 31: 146 and Deichmann 1936: 185” (Muzik 1979). The complete explanation can be found in Deichmann (1936) and Muzik (1979: 168); they serve to confirm the confusion that had developed, through the work of previous investigators, regarding generic status for the species discussed below. In Madsen (1970: 5), “A total of about a dozen gorgonarian species referred or referable to *Swiftia* (syn. *Stenogorgia*) from widely scattered localities in all three oceans have been recorded, but only a few of them are sufficiently described.” In the WoRMS Database (Cordeiro et al. 2018), status of this genus has been accepted, but Breedy et al. (2015: 329) stated that a “thorough review is needed in order to clarify taxonomic problems related to *Swiftia*.” This paper attempts to clarify some of the issues related only to those species that are found in, near, or extending geographically slightly south or north of the California Bight.

##### Swiftia kofoidi

Taxon classificationAnimaliaAlcyonaceaPrimnoidae

(Nutting, 1909)

**Figure 1.**
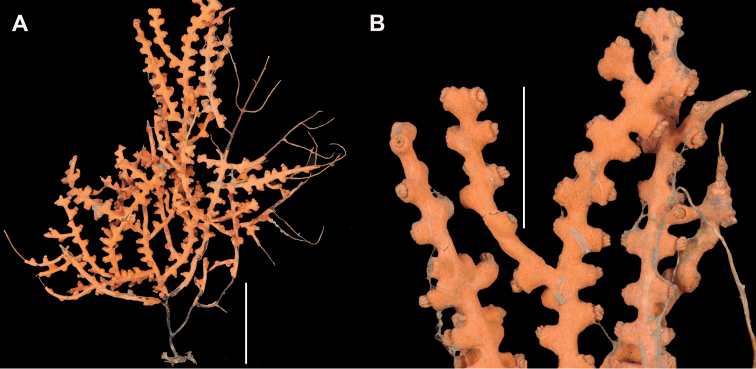
*Swiftiakofoidi*, SBMNH 422965 (delicate orange “morph”). **A** Colony 7.5 cm at tallest point, 7.5 cm broad at widest point **B** Closer view of several branch tips showing distinctive placement of the calyces, creating zig-zag profile of each branch. Scale bars: 2 cm (**A**); 1 cm (**B**).

**Figure 2.**
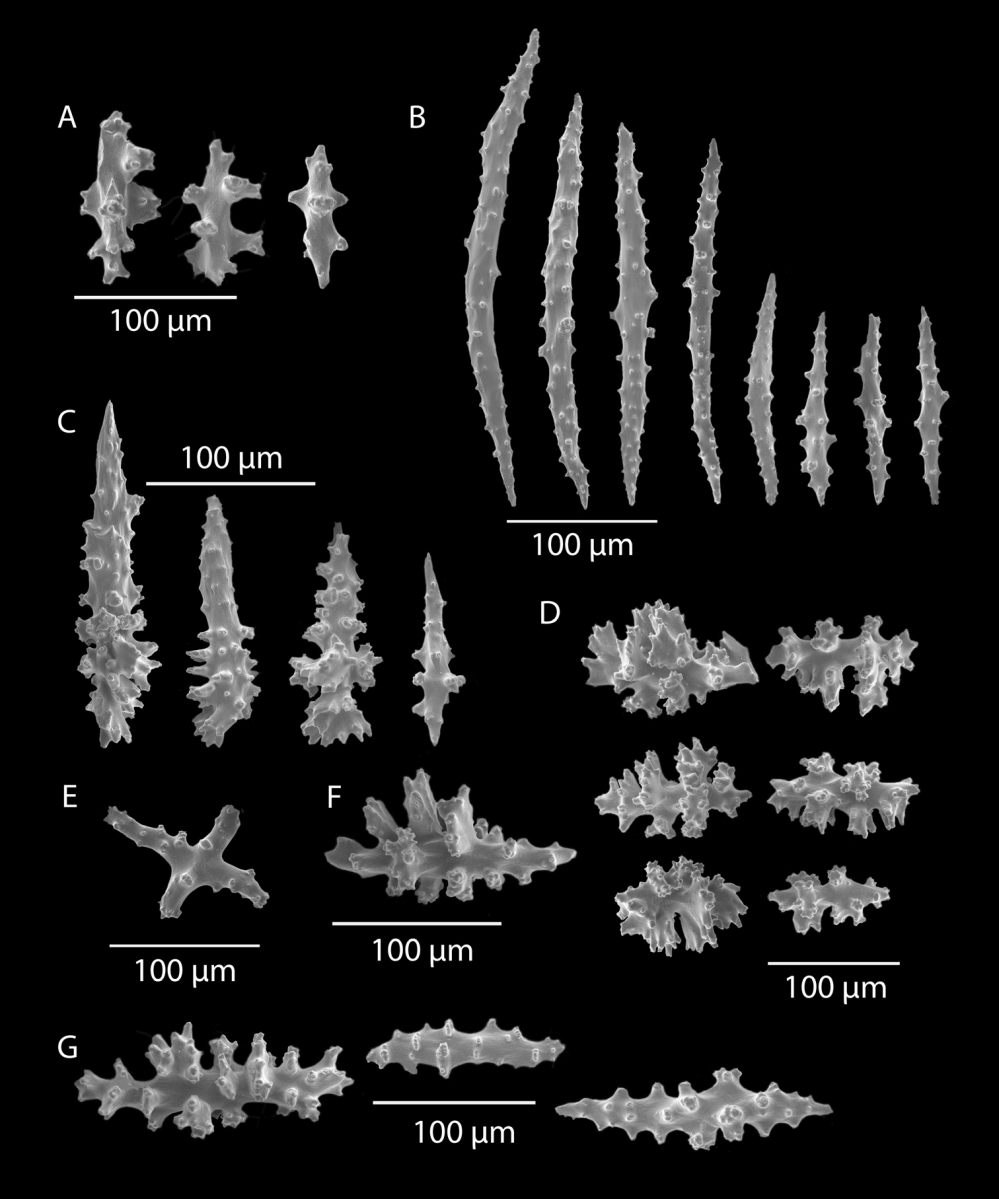
*Swiftiakofoidi*, SBMNH 422965, SEM image. **A** Potential anthocodial sclerites **B** Very elongated spindles, prevalent in species in CA and Mexican waters **C** Irregular spindles **D** Truncated, disk-spindle/capstan forms **E** Unusual quadri-radiate **F** Jagged torch-like form **G** Shorter, jagged spindles

Figures 1A, B , 2A–G , 15A–E , 16A, B , 17A–C

Stenogorgia kofoidi Nutting, 1909: 724, pl 89 (figs 5, 6), pl 90 (fig. 6).

###### Type locality.

USA, California, Monterey Bay, bearing S 67°E, 3.7 mi off Point Piños light-house, ~36°38'00"N, 121°55'00"W, 119–135 m.

###### Type specimens.

**Holotype**USNM 25432 [wet]; specimen was examined.

###### Material examined.

~20–25 lots (see Appendix 3: List of material examined).

###### Description.

*Colony* (Figure 1A) flabellate, loosely branched, sparsely reticulate; often large areas of open space between branches; on rare occasions, branches anastomose; irregular, pinnate branching chiefly in one plane. Appears moderately delicate; some distance from base, main stem generally divides into several main branches, center one ascending nearly unbranched, lateral ones at first widely divaricating, then ascending, giving forth pinnate branches which tend to be opposite, often irregular. Branchlets ~6.0 mm apart (where regular), somewhat flattened (0.5–1.0 mm); terminal branchlets usually end with two oppositely disposed polyps (Figure 1B). Polyps form prominent, conical, volcano-shaped mounds, with broad base (not boxy), with distinct exsert anthocodiae; polyps scattered or biserial (Figure 1B), in two irregular, lateral rows, creating a very narrow front and back; laterally, opposite or (often) alternate on branchlets; colony and branch profile appearing in form of zig-zag pattern. Polyps more numerous on front than behind; mound summits 2.0–4.0 mm apart on one lateral side, 0.6–1.0 mm high, surmounted by 0.4–1.0 mm tall anthocodiae, ~1.5 mm across. Margins with eight-lobed edge; outer sides of tentacles crowded with sclerites, tentacles retractile, bent inward at rest. Coenenchyme thin; outer layer filled with spinous rods, spindles, and capstans. Color of living colony typically true salmon, also bright orange to deep coral red; polyps may be same color (paler), cream or white; axis dark greenish-brown, lightening distally. Sclerites (Figure 2A–G) can be always exclusively small to somewhat long, warty (thorny), slender spindles, larger ones often curved. On shorter, thorny ones, some have very prominent, jagged teeth, projecting off one side (reminiscent of sclerites seen in some *Muricea*); also few small, elongated ones with median waists (“some as granules, some as foliate capstans,” Nutting 1909). In current examinations, those that almost appeared as radiate nuggets could be the granular-appearing or foliate capstans mentioned by Nutting (1909). As well, some can resemble torches, and can be quite evident; these may be the shorter, thorny ones mentioned above. Mound surface sclerites lie transversely. Sclerites in tentacles described as stout, blunt rods; in all specimens examined (identified as this species), rods were sometimes rarely seen in very small numbers, but never obvious.

###### Etymology.

Named in honor of Professor CA Kofoid from the University of California.

###### Common name.

None specifically indicated. Could appropriately be called the Orange ‘rick-rack’ gorgonian.

###### Distribution.

Found off California and Oregon coasts (refer to Appendix 3: List of material examined). Off California in locations between 175 and 218 m, off Oregon in 101–106 m, at 126 m and at 138 m. Speculative range from Upper Baja, California, to NE of Tanner Bank in the south (USNM 59817), along southern California coast through Monterey Bay (**Holotype**, USNM 25432), to south of the Farallon Islands, California (USNM 57235). May continue range northward, off the coast (NW) of Portland, Oregon (USNM 53972), at least (as well, possibly USNM 49538 and USNM 56989, both from Heceta Bank, Oregon). Extreme range makes identification of some specimens questionable; possible range overlap with similar-looking species, *S.pacifica* (Nutting, 1912). If following a slightly deeper, moderately cooler, water gradient in southerly locations, then a possibility. Appendix 2: Map A1 shows apparent range of this species in relation to those such as *S.pacifica* and *S.torreyi* (Nutting, 1909) that it might be confused with when viewed in situ.

###### Biology.

Appears to be a moderate depth to deep-water species, generally deeper off California coast, slightly shallower in more northerly locales. Several lots of this species in SBMNH collection displayed associated organisms: on several, the attached organism looked like a clump of small bubbles, reminiscent of that which “spittle bugs” produce on branches of some land plants; at least some of these clumps were small anemones. Some specimens had species of hydroid attached; few colonies harbored tiny Ophiuroidea.

###### Remarks.

Multiple labels associated with some specimens examined (some with as many as three different labels, each with a different genus/species name, as determined by three separate investigators), complicated identification. This called into question whether all of them were indeed *S.kofoidi*, a different species in the genus *Swiftia* or some other gorgonian (belonging to an entirely different genus); some specimens had been identified previously as belonging to the genus *Psammogorgia* (sclerites did not support this).

Bayer made personal notations in his copy of Nutting (1909), indicating that this species was “stouter than *torreyi*, calyces closer, bars in anthocodiae large and stout. Similar to *Callistephanuspacificus* Nutting, 1912: Pg. 96.” This species is stouter than *S.torreyi* (in the form of the polyps, and in total colony height), but polyps rarely as close together as those in *S.torreyi*, nor were the large, stout bars that Bayer mentioned obvious; additionally, the color is completely different for these two. In other respects, specimens strongly match images for this species given by Nutting (1909). Bayer’s notation regarding similarity between it and *Callistephanuspacificus* Nutting, 1912 is generally affirmed. Overall, most similar to *Swiftiapacifica*, but *S.kofoidi* is often more open and delicate; many colonies of *S.pacifica* can be thicker-branched and bulky in overall appearance (Figures 15, 21). The bright orange color (salmon), overall delicate appearance, widely-spaced polyp mounds and very distinct, jagged/sharp zig-zag profile (due to predominantly alternate and lateral placement of prominent polyps) of this species set it apart from the other two species (*S.pacifica* or *S.torreyi*) that it is often compared with. As indicated in the WoRMS Database (Cordeiro et al. 2019), this species has accepted status.

The California Academy of Sciences (CAS) has some thirty cataloged records (only some identified to species) from this genus in its collection. Eleven of them are from Alaska and are likely *Swiftiapacifica*. Of the remaining, sixteen or so were identified as this species. Most of these however, were collected from northern California (Monterey Bay, Humboldt County, Sonoma County, Marin County; many likely the more northerly-dwelling *S.pacifica*). The Monterey Bay Aquarium Research Institute (MBARI) collection records indicated specimens of this species, collected and/or surveyed/photographed throughout their study areas (of those identified as this species, many may actually be *S.pacifica*). One colony examined via video/photo, was T1101-A21; based on colony shape and color, it appeared to be this species. Sclerite examination of the actual specimen could have confirmed it as such but specimen was not collected. Two other data collection events should be considered. However, neither of the specimens in question were located; they apparently are not housed at the National Museum of Natural History, Smithsonian (NMNH). Both from: North Pacific Ocean, USA, California, Monterey County: [Monterey Bay], bearing S 46°E, 8.4 miles off Point Piños light-house and S 76°E, 3 miles off Point Piños light-house, at 1,544 m and 109 m, respectively. Both collected by USBCF ‘Albatross’, at stations 4546 and 4554, respectively.

##### Swiftia pacifica

Taxon classificationAnimaliaAlcyonaceaPrimnoidae

(Nutting, 1912)

**Figure 3.**
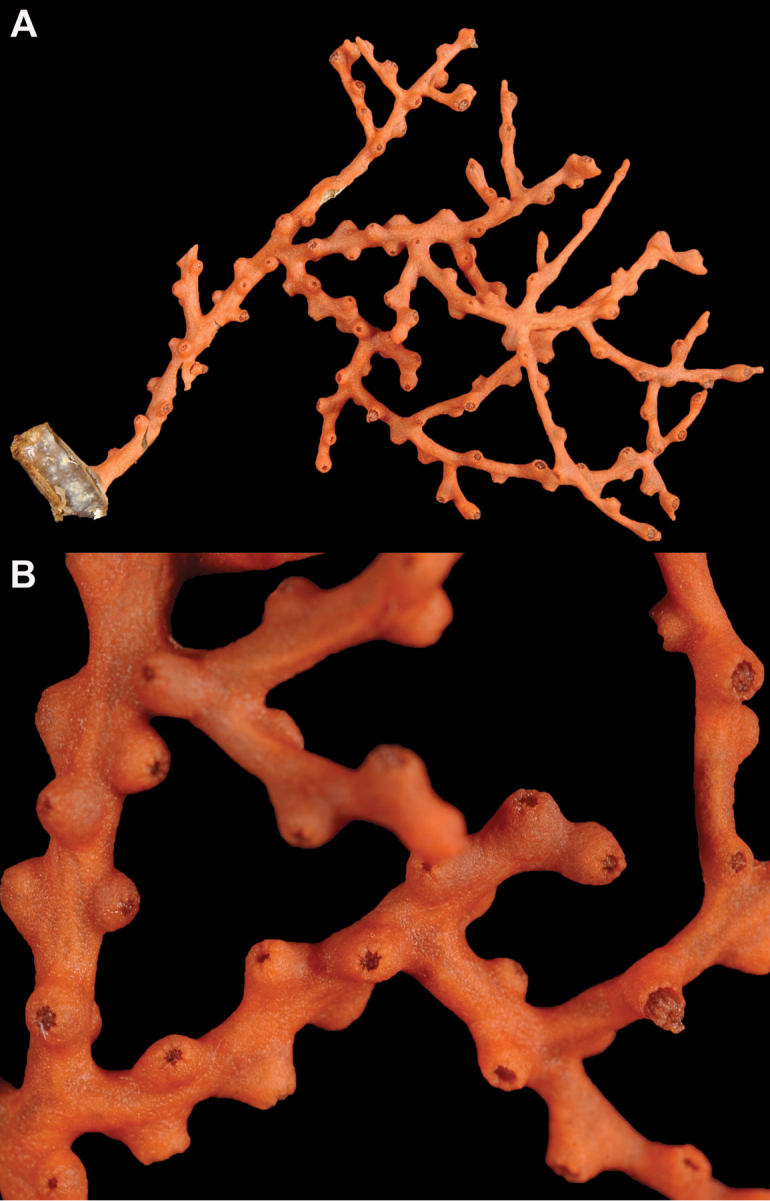
*Swiftiakofoidi* [? *Swiftiapacifica*] (thicker dark red “morph”), SBMNH 232036. **A** Colony measures 6.0 cm tall, 5.5 cm broad at widest point, demonstrating zig-zag appearance of branches due to calycular placement **B** Branch close-up, showing placement of prominent conical calyces on branches; calyces measure ~1.0 mm tall.

**Figure 4.**
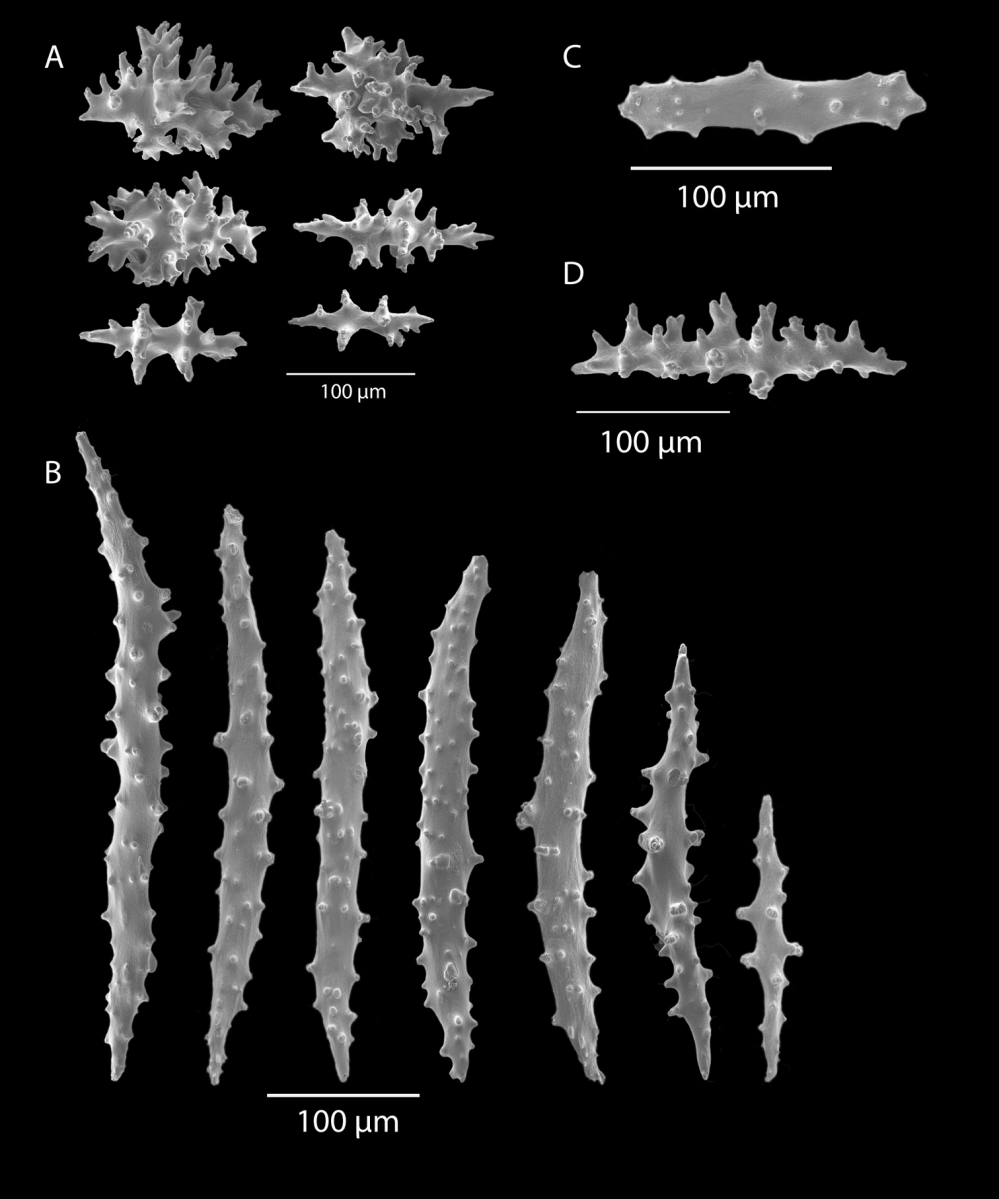
*Swiftiakofoidi* [? *Swiftiapacifica*], SBMNH 232036, SEM image. **A** Truncated, jagged disk-spindle/capstan-like forms **B** Elongated spindles **C** Might be a typical “fingerbiscuit-rod” form typical of the genus; seen very rarely, if at all in this species **D** An irregular spindle.

**Figure 5.**
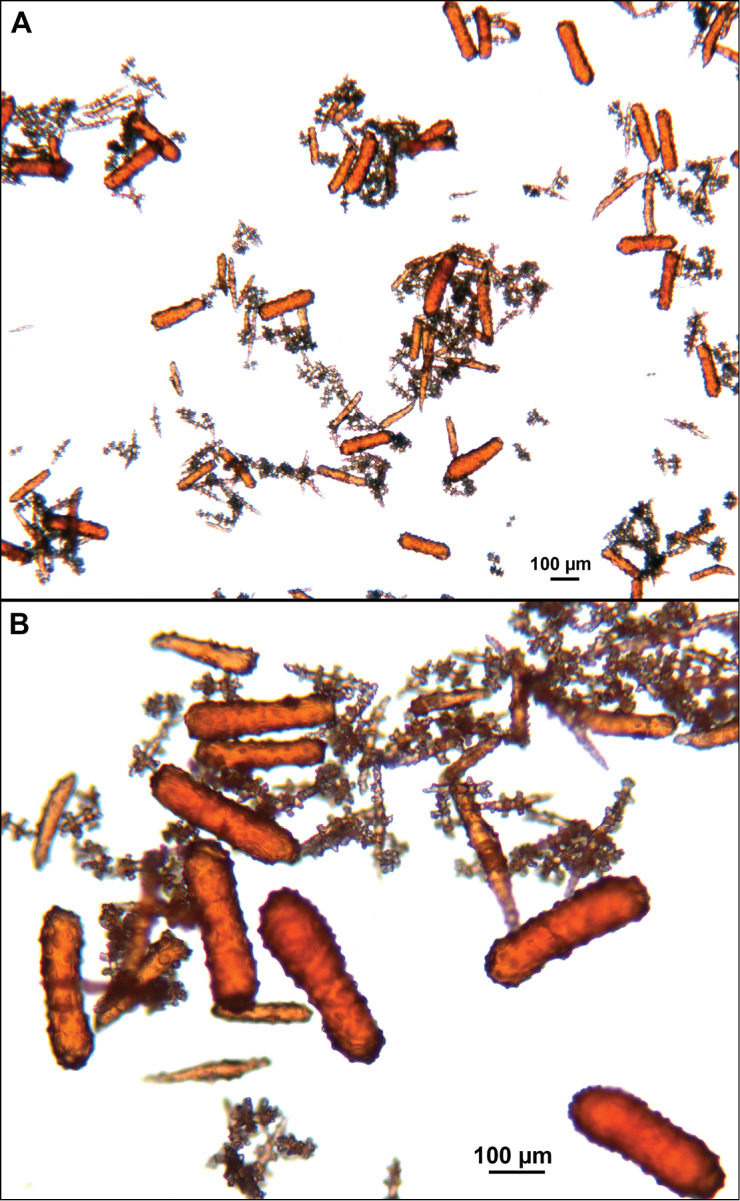
*Swiftiapacifica*, specimen 41-39-1 (Alaska Fisheries Service, Gulf of Alaska); looking in same form of that seen in Figure 3, light microscopy arrays. **A** (4×) showing variety of sclerites, particularly the characteristic “fingerbiscuit-rod” seen in the genus *Swiftia*. Sclerites from specimen examined for Bob Stone, Alaska Fisheries Service **B** Higher magnification, 10×, showing all sclerite forms, including obvious anthocodial fingerbiscuit-rods. The larger spindles measure ~300 µm long, smaller spindles of ~200 µm, and the rods range from 308–370 µm in length.

**Figure 6.**
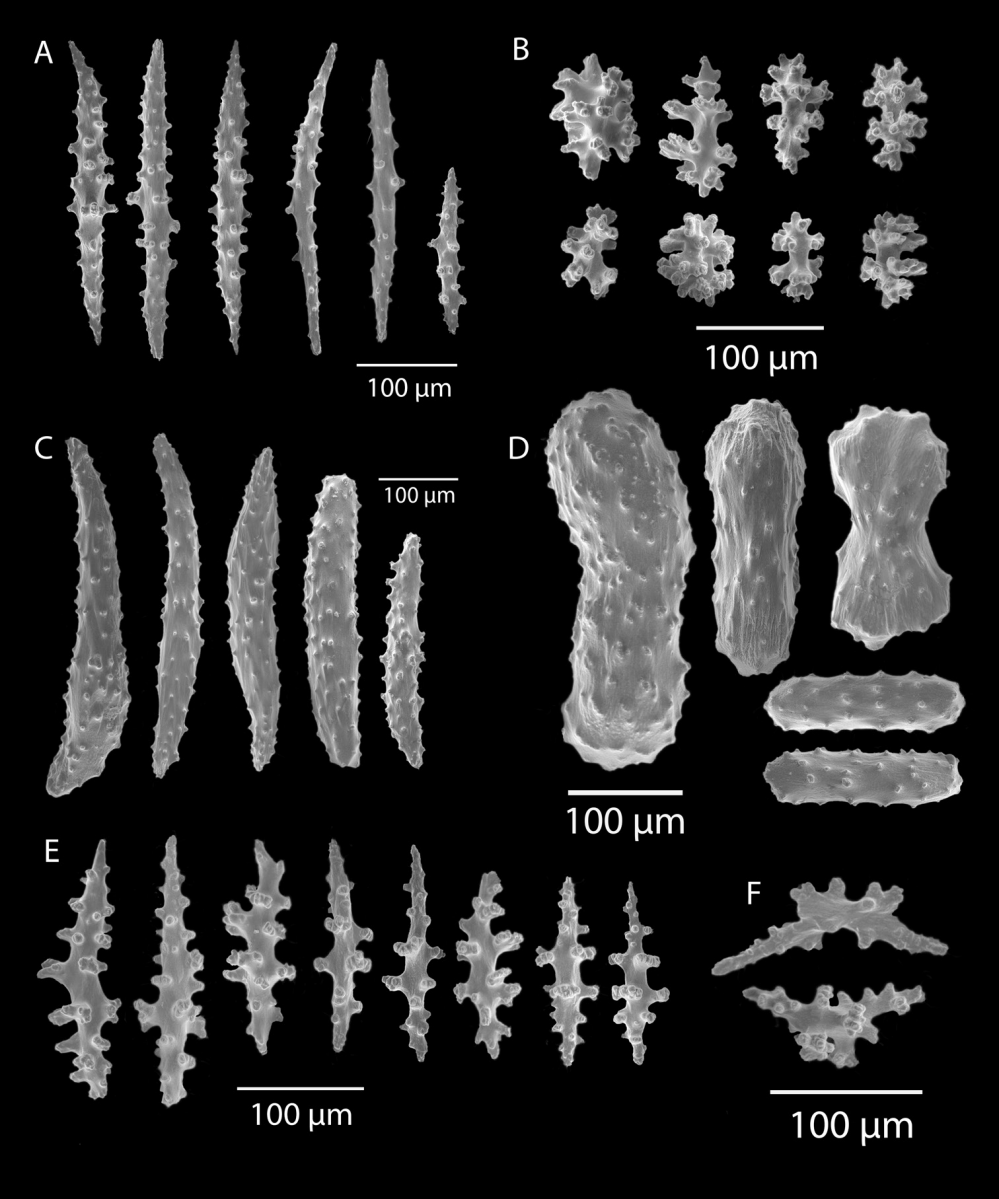
*Swiftiapacifica*, specimen 41-100A-2 (Alaska Fisheries Service, Gulf of Alaska, via Bob Stone), SEM image. **A** Long spindles **B** Jagged disk-spindle/capstan-like sclerites **C** Thick, elongated rod-like spindles **D** Anthocodial “fingerbiscuit-rod” forms characteristic of the genus **E** Irregular spindles **F** Odd irregular spindles.

Figures 3A, B , 4A–D , 5A, B , 6A–F , 21A, B , 22A–D , 23A, B

Swiftia pacifica (Nutting, 1912): 96, 97, pl 14 (figs 2, 2a) and pl 21 (fig. 6) [= Stenogorgiapacificus Nutting, 1912]: Muzik 1979: 168–173, fig. 26, pl XXVI.
Callistephanus pacificus Nutting, 1912: 96, 97.
Allogorgia exserta Verrill, 1928: 8.
Swiftia rosea pacificus (Nutting, 1912): stat. nov. Madsen 1970: 8.

###### Type locality.

For holotype, unknown (erroneously labeled); for type, ‘Albatross’ Station 4781, 52°14'30"N, 174°13'00”W, south and east of the Bering Sea. (See Remarks below.)

###### Type specimens.

**Holotype**USNM 49513 (colony portion only); **Type**USNM 30024; both specimens were examined.

###### Material examined.

~23 lots (see Appendix 3: List of material examined).

###### Description.

*Colony* moderately sized (up to 18–19 cm tall), planar, flabellate, flexible, rubbery in appearance (Figure 3A); branches nearly forming net-like reticulations, but usually not anastomosing; generally, moderate open appearance to branches. Main stem extends upwards some few cm (above base), 1.0–2.0 mm wide; branches from main stem opposite or alternate, coming off at 45° to 90° angles, then tending upwards; distance between branches 0.5–2.0 cm; terminal branches to 2.0 cm long, l.0 mm in diameter. Polyps lateral (mostly alternate, sometimes opposite), very few on front, with back generally free of polyps, thus flat; polyps conical (sometimes tubular), three, four or five per cm, arising from small mounds (Figure 3B); anthostele 0.5–2.0 mm H, 1.0–2.0 mm W (seeming rather broad and boxy), anthocodiae preserved exert up to 2.0 mm long, but often appearing as dense tuft with 1.0 mm or less showing above polyp mound. At distal end of terminal branches, two (or two pair) oppositely disposed polyps. Color of colony bright to deep crimson or muddy red (brick-red) in life, but both darker and lighter red (dull pink) colonies occur; polyps sometimes dark greenish-grey; sclerites bright red or orange (rods) to pale pinkish-red (most common color, usually true of spindles and capstans). Sclerites (Figures 4A–D, 5A, B, 6A–F) symmetrical; unilaterally developed superficial capstans and spindles, 0.08 to 0.17 mm long in coenenchyme; flatter warty spindles to 0.35 mm in layer below; also eight radiates. Axial sheath sclerites short (to 0.12 mm), narrow-waisted, blunt-ended. Anthocodial bases can have numerous prominent blunt bars (fingerbiscuit rods, in shape comparable to a bacterial rod), curved or straight (Figures 5B, 6D); arranged transversely at bases, more longitudinal at distal end. Examination of multiple specimens did not always reveal presence of rods, but when present, very obvious. Pinnular scales 0.06–0.1 mm long.

###### Etymology.

Species name likely refers to locality where type specimen was collected, outer Aleutian Islands, Alaska in the North Pacific.

###### Distribution.

North Pacific Ocean, Aleutian Islands, Alaska down to California (not a common occurrence), and from Alaska down through western Pacific to Hawaii. Range determined from collection location information provided with specimens examined (see Appendix 3: List of material examined).

###### Biology.

Usually bathyal, but depth range extends from ~18 to ≥ 2,000 m, based on depth information provided with specimens examined.

###### Remarks.

Bayer made personal notations in his copy of Nutting (1909); species is quite similar to *Swiftiakofoidi*. In overall shape, this species ranges from a rather open-spaced and delicately appearing colony (rarely) to one that seems bulkier. The zig-zag profile is evident but is much more rounded (less jagged-looking) than that of *S.kofoidi*. Polyp mounds in *S.pacifica* are somewhat lower, more rounded than those seen in *S.kofoidi*. In Nutting’s 1912 description, he noted this species’ “very close resemblance to *Calllistephanuskoreni* Wright and Studer” but also added that “(g)eographical considerations render it unlikely that the two are identical.” Madsen (1970) noted that the species described here so closely agreed “with the Scandinavian *rosea* (it too, has radiate capstans among the spicules) that it is reasonable to consider it its amphiboreal representative, and to regard it as a subspecies of *rosea*, stat. nov. [*Swiftiarosea* (Grieg 1887)];” an example of a discontinuous circumboreal octocoral.

Examination of specimens collected in the Gulf of Mexico, 2009 (provided by P Etnoyer, NOAA’s National Ocean Service Office, South Carolina), indicated Madsen may have been correct. Three specimens were sent (without collection data). Sclerite preparations were performed, and specimens were tentatively identified as *S.pacifica*; when informed (pers. comm. from A Quattrini, then a doctoral candidate, Temple University) that these three were actually collected from the Gulf of Mexico, further investigation was warranted. Specimens of *S.pallida* Madsen, 1970 had been examined and sequenced (via barcoding of those specimens), and a close match was found between *S.pallida* specimens and other lots of the same specimens examined. Referring back to Madsen’s (1970) discussion, *S.pallida* was considered by Madsen to be, at most, a subspecies of *Swiftiarosea* (*Swiftiaroseapallida* Madsen, 1970), of the north Atlantic, based on the color of its colony form (pale gray) and sclerites (colorless). *S.pacifica* from the Pacific Ocean, examined here (considering Madsen) looked very similar to the species *S.rosea* (which could certainly be the correct species identification for the three specimens from the Gulf of Mexico sent by Etnoyer) in its colony color, branch detail, arrangement of polyps and shape of its sclerites. It appeared that *S.pacifica* (eastern North Pacific), *S.rosea* of the Atlantic and its subspecies, *S.pallida* (a northern Atlantic bathyal form) were strongly related. *S.rosea* is the nominate form, found not only in the bathyal North Atlantic, but also in the Scandinavian sublittoral (Madsen 1970). It would appear that the species described here could be the Pacific Ocean extension (of the Atlantic species *S.rosea*) in its distribution, having moved into the Pacific Ocean via waters circumscribing the North Pole. It can be inferred that as *S.pacifica* appeared in the Pacific, it dispersed down the western coast of the North American continent (at least as far as, generally, central California), just as *S.rosea* has apparently spread across the north Atlantic and down the eastern side of the North American continent (and presumably, into the Gulf of Mexico, perhaps as a new subspecies). The WoRMS Database (Cordeiro et al. 2019) shows the accepted status of this species.

Muzik (1979) noted discrepancies regarding locality for the holotype. The specimen is housed in the Bishop Museum, Hawaii (as *Allogorgiaexserta*, #101), with a portion of it housed at NMNH (USNM 49513). This specimen “agrees in details of color, sclerites, and polyp size and shape with the type from Alaska of Swiftiapacifica (USNM 30024) collected from the Aleutians in 1906 and described by Nutting in 1912. One can conclude that there was an error in the locality of the so-called Hawaiian specimen. It is entered in the Bishop Museum catalog as ‘Albatross’ 2742 without locality. Entry 2741 is from Station 3353 off Panama. Prior to that station, the ‘Albatross’ had been collecting in the Pacific Northwest, so it is conceivable that this S. pacifica was collected there and later confused.” It appeared that normal range for *Swiftiapacifica* is from central/northern California northward, but occasionally may appear south of that range. CAS has thirty cataloged records from this genus; of these, eleven lots are from Alaska, and are likely *Swiftiapacifica*.

There appeared to be a distinct morphological trend, from southern to northern waters, along the California coast up through the coastal areas of Oregon, Washington and Alaska that required discussion; a proposed explanation for the range distribution of this species follows the descriptions of all species (with red color) found in or near the Bight covered in this paper. Briefly, an extensive examination of colonies collected from Baja California to the remote northern aspect of the Bering Sea (see Appendix 3: List of material examined) revealed a very distinctive trend in the appearance of colonies and sclerites from south to north. The sequential trends seen within the two species, *S.kofoidi* and *S.pacifica* (or morphs of one) are discussed in the Further Remarks section (along with variation in sclerite morphology of other eastern Pacific *Swiftia* species). The observation of this phenomenon has never been discerned or noted previously.

##### Swiftia simplex

Taxon classificationAnimaliaAlcyonaceaPrimnoidae

(Nutting, 1909)

Figures 7 , 8A, B , 9A, B , 10A–H , 24A–D , 25A, B , 26A–C , 27A, B

Psammogorgia simplex Nutting, 1909: 720, pl 88 (figs 4, 5), pl 90 (fig. 4).

###### Type locality.

[USA], California, Santa Cruz Island, bearing N 35°E, 7 miles off Point San Pedro, ~34°02'02.76"N, 119°31'11.77"W, 447–510 fm [813–927 m].

###### Type specimens.

**Syntype**USNM 25431 and USNM 43130 [both wet]; both specimens were examined.

###### Material examined.

~24 lots (see Appendix 3: List of material examined).

###### Description.

**Figure 7.**
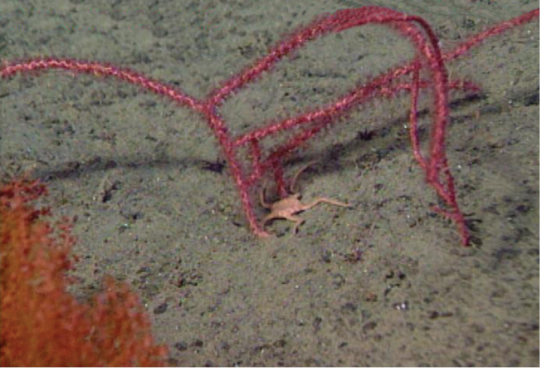
In situ shot, identified as *Swiftiasimplex*. This is what one would expect to see of live colony. Notice strong similarity to that seen in Figure 28, Part II. As specimen was not collected, identification cannot be confirmed. Image (02_57_27_20) courtesy of Lonny Lundsten and Kim Fulton-Bennet, “(c) 1992 MBARI.”

**Figure 8.**
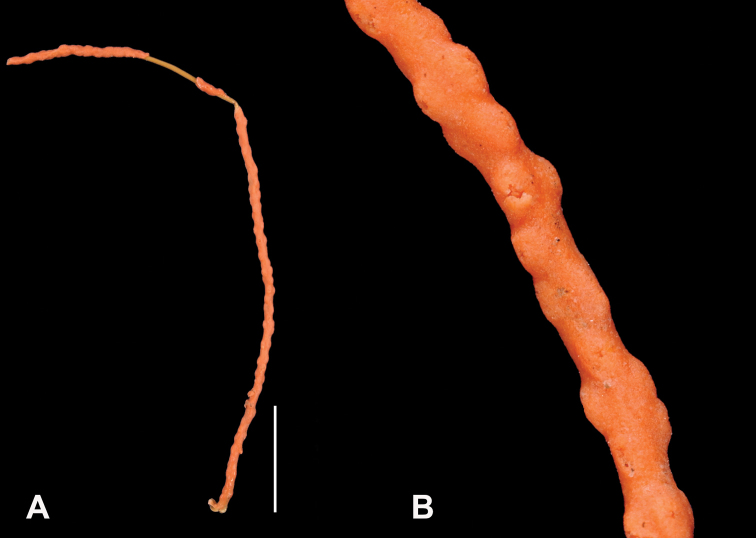
*Swiftiasimplex*, SBMNH 422979. **A** Entire colony, ~21 cm long, diameter ≤3 cm, including calyx **B** Branch close-up showing calyx placement on branch. Scale bar: 3 cm (**A**).

**Figure 9.**
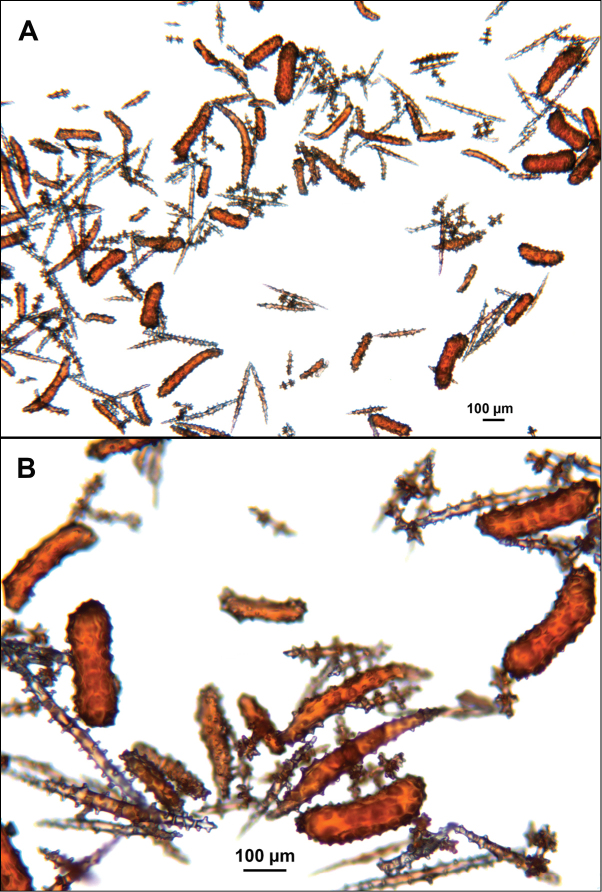
*Swiftiasimplex*, specimen #81-99B-1 (Alaska Fisheries Service), light microscopy arrays. **A** 4× magnification, showing long, slender spindles and anthocodial fingerbiscuit-rods **B** 10× magnification of sclerites, emphasizing fingerbiscuit-rods of anthocodium. These rods measure between 340–350 µm, while long, thin spindles measure between 430–500 µm. Specimen provided by Bob Stone, Alaska Fisheries Service.

**Figure 10.**
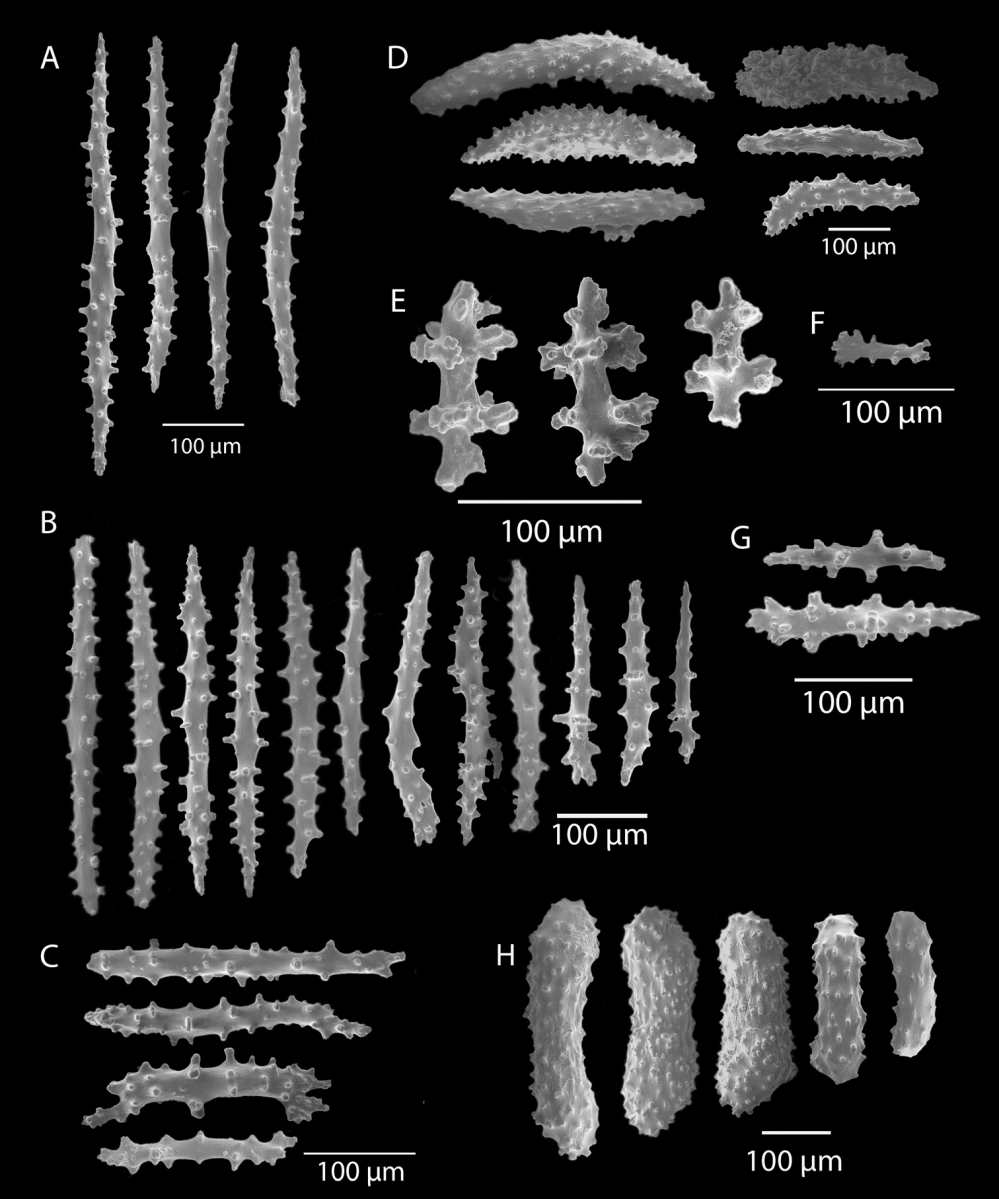
*Swiftiasimplex*, specimen #81-99B-1, SEM image. **A** Elongated spindles **B** Moderate-length spindles **C** Shorter spindles **D** Thick, elongated rod-like spindles **E** Less jagged double-disk/capstan-like forms **F** Tiny, odd spindle **G** Irregular spindles **H** Anthocodial “fingerbiscuit-rod” forms typical of genus. Alaska Fisheries Service.

*Colony* (Figures 7, 8A) straggling, whip-like, not always erect; branched slightly, mostly unbranched; largest specimens ~13+ cm tall. Stem round, slender, of uniform diameter throughout. On stem/branches, polyps uniformly distributed on all sides, not crowded (Figure 8B), up to 2.0 mm apart; tubular, small, ~1.0 mm high, usually higher than broad; when polyps contracted, nearly flush with branch surface. Coenenchyme moderately thick; color of living colony, including polyps, salmon, brick reddish-pink (commonly) to coral-red throughout; sclerites reddish-pink; rods reddish-orange; sometimes long, warty spindles colored and colorless. Sclerites (Figures 9A, B, 10A–H) of three main kinds: 1) small double-spindles, rosettes, stars and/or small clubs, found mostly in superficial layer of coenenchyme (all much less numerous than second kind; clubs much less numerous than other kinds); 2) larger spindles, slender, pointed, some slightly curved, covered with regularly distributed small warts (Figure 10A–C); 3) not always numerous, but when present, very conspicuous, colored anthocodial rods (fingerbiscuit rods; Figures 9A, B, 10H); moderately to heavily warted, much shorter than long spindles, longer than first type. Polyps, generally, with spindle-shaped sclerites in walls and near/on tentacular bases, arranged more or less in chevrons. Otherwise, longitudinally arranged.

###### Etymology.

Earlier genus designation (Nutting, 1909), *psammo*- = sand); *simplex*- = simple, perhaps referencing the very simple, usually unbranched colony, found generally on soft-bottomed sites. However, no derivation for species name given in Nutting’s (1909) description.

###### Common name.

Whip coral (suggested: Brick-red whip coral).

###### Distribution.

Kükenthal (1919), in Chun: California, coast to abyssal. Total distributional range (surmised from collection location data reported by various institutions) extends from southern California Channel Islands (and further west--San Juan and Rodriquez Seamounts), up the coast of California (Monterey Bay, Carmel Canyon), sparsely along Oregon coast (Tillamook Head, Columbia River), to Washington coast (Grays Harbor, Quinault Canyon, Queets; general site locations off Oregon and Washington gleaned from NMNH material), up to Gulf of Alaska, found on seamounts and elsewhere (NMNH material, Alaska Fisheries Service). Fairly recent collection event (2008, Olympic Coast National Marine Sanctuary) produced at least one sample that may be this species, collected at ~48°07'53"N, 125°05'20"W at 335 m.

###### Biology.

Appears to prefer at least subtidal depths, generally deeper, according to collection location data; frequently encountered on Seamounts (~190–900 m). MBARI T630-A13 had attached to it what appeared to be a cluster of white eggs (cluster identity not determined); these flexible branch strands, projecting up into localized water currents, would make good attachment sites for eggs needing oxygenation and/or flow to keep them clean, being suspended above muddy bottoms found at depth.

###### Remarks.

CAS has three specimens (likely this species), all from northern California (Cordell Bank, W of Point Reyes, CAS-IZ-96739; off Pigeon Point in San Mateo County, CAS-IZ-96744 and Eel River Canyon in Humboldt County, CAS-IZ-96758), labeled as *Euplexaurasimplex*. This is a hitherto unknown application of a genus name, done by D Harden in the early 1970s (likely, an attempt to be comparable with the then named *Euplexauramarki*). This genus designation is incorrect; the specimen is the species *Swiftiasimplex*. While Nutting (1909) placed it in the genus *Psammogorgia* Verrill, 1868a, geographic location of specimen(s) he described, geographic locations of specimens examined here, appearance of sclerites, along with molecular work conducted by M Everett et al. (2016), do not warrant that species designation either. While the WoRMS Database (Cordeiro et al. 2019) did indicate an accepted status for this species designation under the genus *Swiftia*, it also showed accepted status for the species as *Psammogorgiasimplex* (Cordeiro et al. 2018). Based on the genus description for *Psammogorgia* by Verrill (1868a), Bayer (1958) on the morphological characters mentioned above and discussion provided by Bayer and Deichmann (1960), which also discussed the probability of appearance in the Panamanian province, material examined in this work warrants placement in the genus *Swiftia*, not in the genus *Psammogorgia*.

Sclerite examinations revealed a few individual colonies (several species) in the genus *Swiftia* (such as that shown in Figure 8), with minimal/no fingerbiscuit rods. Nothing examined and identified as *Chromoplexauramarki* (Kükenthal, 1913) (species closest in superficial colony appearance) ever displayed these rods, as expected for this genus. It was easy to understand how identification done in the field, on in situ colonies (with water depth distorting color), could label colonies from the two species (this and *C.marki*) as the same organism. Current examinations discussed here shed some light on the confusion. The explanation provided regarding *S.pacifica* in Further Remarks section, is an attempt to clarify (and explain) why some colonies of *Swiftia* have scleritic anthocodial rods and others do not.

California specimens identified from the genus *Euplexaura* (now the genus *Chromoplexaura* Williams, 2013) on several MBARI video clips that were viewed could actually be this species. *C.marki* (which this species can so closely resemble), is usually bright deep red, with white or pale yellow anthocodiae/polyps (Kükenthal 1913, 1924; also Johnson and Snook 1927) but not always (see discussion, Part II, this work) while this species is a dull pinkish to brick red color, with colony coenenchyme and anthocodiae/polyps the same color; sclerites are very different for the two genera. It is likely that MBARI is seeing both this species and *C.marki*, but not able to clearly distinguish between the two due to color distortion at depth under field conditions, if not collecting.

A specimen (R1159_EPI_164_0015) collected by Olympic Coast National Marine Sanctuary in 2008 superficially appeared to be this species; polyps were mostly contracted into very round, prominent mounds, although these had larger dimension than that given in the above description (tentacles were more or less the same salmon color as the coenenchyme, but polyp bodies, closely proximal to branch, were white when dissected out). Based on further examinations, specimen was tentatively identified as *Swiftiaspauldingi* (Nutting, 1909); however, lack of fingerbiscuit rods points in the direction of *Chromoplexauramarki*. Recent DNA sequencing (communications with M Everett, NOAA affiliate, 2013–2014) indicated that some *Swiftia* species might need subdividing (three different species or variants a possibility).

##### Swiftia cf. spauldingi

Taxon classificationAnimaliaAlcyonaceaPrimnoidae

(Nutting, 1909)

**Figure 11.**
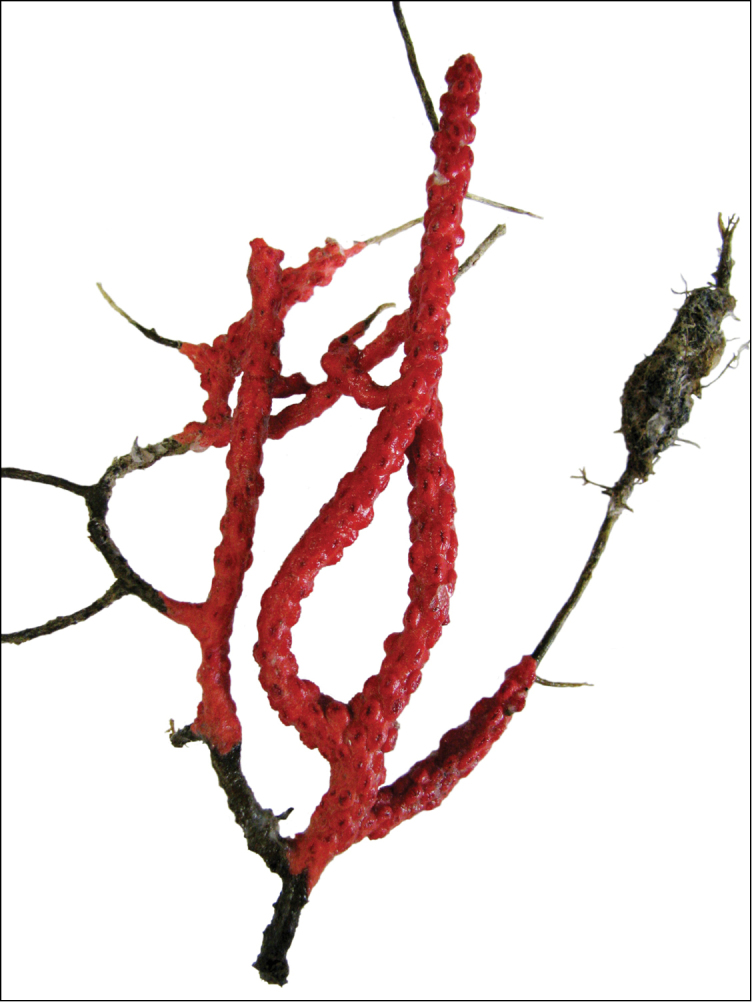
*Swiftiaspauldingi*, CB#34806-455. Full colony height approximately 12 cm. Image taken by Carla Stehr, courtesy of Ewann Berntson, National Fisheries Science Center, Port Orchard, WA.

**Figure 12.**
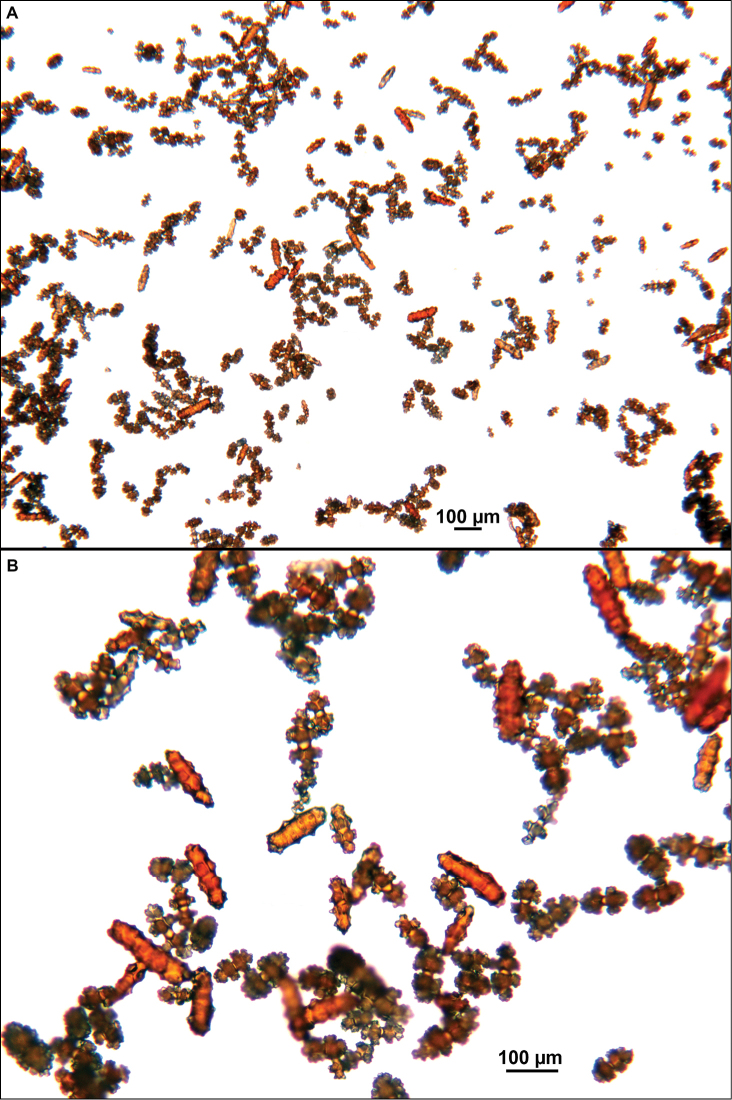
*Swiftiaspauldingi*, CB#34806-455, light microscopy arrays. **A** 4× magnification **B**10× magnification; anthocodial fingerbiscuit-rods very obvious, measuring from 128–171 µm. The dense “ovals” measure between 100–115 µm; smallest sclerites are ~86 µm.

**Figure 13.**
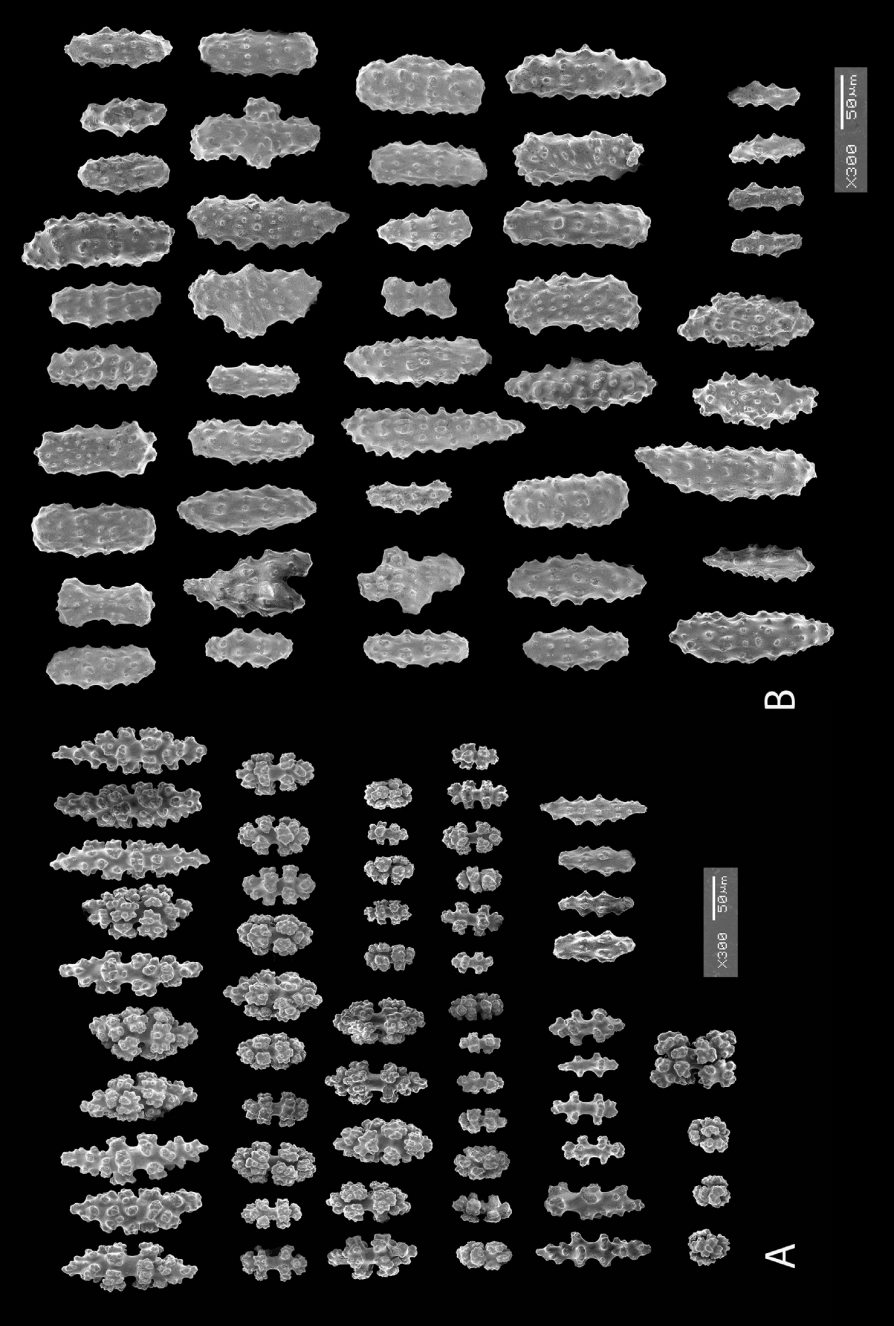
*Swiftiaspauldingi*, CB#34806-455, SEM image. **A** An array of coenenchymal sclerites (spindles) **B** An array of, primarily, “fingerbiscuit-rods,” the characteristic sclerites for the genus. Images prepared by Carla Stehr, courtesy of Ewann Berntson, National Fisheries Science Center, Port Orchard, WA.

Figures 11 , 12A, B , 13A, B

Psammogorgia spauldingi Nutting, 1909: 721, 722, pl 89 (figs 3, 4), 90 (fig. 7).
? = Euplexauramarki Kükenthal, 1913: 266; noted by Bayer 1979: 1034.
? Chromoplexauramarki (Kükenthal, 1913): Williams 2013.

###### Type locality.

[USA], North Pacific, California, Monterey Bay, Pacific Grove.

###### Type specimens.

**Holotype**; transferred from Hopkins Marine Laboratory Collection; [USNM 91854, wet]; specimen was examined.

###### Material examined.

~9 lots (see Appendix 3: List of material examined). [See also discussion regarding “red whip” forms, in Remarks section for species *Chromoplexauramarki* discussed in Part II of this work.]

###### Description.

*Colony* (Figure 11) low, moderately bushy (tending to one plane); flabellate. Sparsely branched; irregularly dichotomous, subdividing some distance above base; branches round in cross section. Terminal branches somewhat stout, 5.0–10.0 cm long, as large as main stem, nearly as round (2.0–3.0 mm thick); slender, whip-like. Polyps scattered closely, uniformly, over surface on all sides, as very low, fairly large, somewhat rounded warts; in some specimens, scarcely raised above general surface of colony, almost entirely included, hardly evident (flush), seeming to be nearly absent, yet in others readily visible; less than 1.0 mm across, 1.0 mm apart. Color of living colony bright coral or salmon-orange; sclerites range from yellowish to very pale red (most commonly, moderate to pale salmon pink), with orange rods; polyps, in preserved specimens, appear to be pure white, while in some colonies (preserved) can appear light salmon-pink. Sclerites (Figures 12A, B, 13A, B) of several kinds, but generally small, short, exceedingly warty spindles and double-spindles. Sclerites in body wall of polyps somewhat longer, more slender spindles (double-spindles), with more delicate warts and points, tapered with wide, median space, but stout, scarcely acute (but never as long as those seen in other eastern Pacific species of *Swiftia*). Bayer (1956) noted these longer sclerites as symmetrical or with warts on one side simple and conical, elsewhere compound. With appearance of two or three whorls of large, compound, rough warts on each end, those nearest middle usually the largest. These longer sclerites tend to longitudinal arrangement in body wall in eight rows; rows sometime extending part way up outer sides of tentacles. Stouter sclerites (double-spindles) also tapered with wide median space, but shorter, blunt, each end with two or three crowded, usually somewhat confused whorls of large rough warts, forming large terminal cluster. (Bayer, 1956 noted these sclerites as having warts of one side fused like those of disc spindles). In some colonies identified as this species, presence of sclerite form approaching that of double heads (Figure 13A, top row), with narrow median space and large cluster of closely crowded warts on each end, resembling dark, dense triangular tip; these sclerites are of particular interest in comparison with *Chromoplexauramarki* and the double dunce-cap sclerite. Other heads shorter, lacking median space, entirely covered with crowded warts. Crosses, with four short, roughly-warted branches said to occur frequently; not evident in examinations undertaken. Fingerbiscuit rods (Figure 13B) more heavily warted than those seen in other species from genus (but may not be abundantly present).

###### Etymology.

Named in honor of Mr MH Spaulding from Stanford University.

###### Distribution.

Rarely, southern California (Los Angeles County); may extend from central California, northern California Channel Islands, north to coast of Washington State (Strait of Juan de Fuca). Distribution based on specimens examined with collection location data, from several sources (NMNH, NOAA offices, MBARI). The specimen collected by Olympic Coast National Marine Sanctuary in 2008, collected at 335 m, ~48°07'53"N, 125°05'20"W, confirmed WA coastal waters as a location for this species.

###### Biology.

Conspicuous Ophiuroidea found intertwined on branches, as those seen on specimen from “Oregon State, R/V ‘Yaquina’ NH15” (SBMNH 423073) and that collected by Olympic Coast National Marine Sanctuary in 2008.

###### Remarks.

Multiple labels (NMNH) were associated with some specimens examined, along with differences in literature usage of the genus name *Swiftia* and at least one specimen at NMNH had been given the name *Psammogorgiaspauldingi* (while elsewhere, Bayer’s SEM files, the folder of SEM images for this same specimen, was labeled “*Leptogorgiacaryi* = *Swiftiaspauldingi*,” with the numbers “57157, SEM 2787 & 2790” [note use of the genus name *Swiftia*]; however, this synonymy designation is in error).

The discussion of *Chromoplexauramarki* in Part II (along with remarks given for *S.simplex*) is pertinent. At least one specimen of *C.marki* examined had sclerites very similar to those seen in *S.spauldingi*, but there were no anthocodial fingerbiscuit rods. Could *S.spauldingi* sometimes be seen as a less-branched colony, resembling *C.marki* (or be very unbranched, and look more like *S.simplex*)? *S.simplex*, *S.spauldingi* and *C.marki* can have similar colony appearance; but the first two will have the fingerbiscuit rods, and only the latter two will have sclerites showing other similarities of form (but not entirely). A key difference (and justification for keeping the two, *C.marki* and *S.spauldingi*, as separate species) is the consistent lack of fingerbiscuit rods in *C.marki*, but which does have the unusual double dunce-cap spindles that are only uncommonly seen in *S.spauldingi* (and in this latter species, usually smaller-sized; refer to Figure 13A). Bayer (1979: 1034) offered support for a synonymy between the two, but this synonymy seems questionable. More specimens will need to be collected and examined. Despite confusion regarding the status of this species, Cordeiro et al. (2019), shows *S.spauldingi* as the accepted name, with *Psammogorgiaspauldingi* the only synonymized name.

##### Swiftia torreyi

Taxon classificationAnimaliaAlcyonaceaPrimnoidae

(Nutting, 1909)

Swiftia torreyi Nutting, 1909: 721 pl 89 (figs 1, 2), pl 90 (fig. 5) [= Psammogorgiatorreyi Nutting, 1909].

###### Type locality.

[USA], California, Monterey Bay, 36°38'00"N, 121°55'00"W (bearing S 78°E, 6.8 miles) off Point Piños light-house, 755–958 fm [1373–1742 m].

###### Type specimens.

**Holotype**USNM 25433, [wet]; specimen was examined.

###### Material examined.

None of the material examined (~16 lots) came from the SBMNH collection (see Appendix 3: List of material examined).

###### Description.

*Colonies* strictly flabellate (usually), ~15–30 cm tall, ~16–17 cm in breadth. Branches commonly anastomosed; branches dense, closely spaced. Main stem bears branches on opposite sides separated by distance of 4.5 mm to +7.0 mm; branches generally thin (no more than 1.0 mm wide) in appearance. The whole forms a loose reticulation, somewhat comparable to that seen in a few species of the genus *Pacifigorgia*, such as *P.gracilis* (Kükenthal, 1924). Polyp mounds slightly truncated to (commonly) tubular cones, 1.0 mm high or less, can be as wide as high; extended polyp can add ~1.0 mm to height; distributed primarily on sides of branches, ~2.0 mm or less apart on one lateral side. In front view, there appeared to be two opposite rows, but can be alternate; body and tentacles of polyps tend to bend (curl) toward front of colony somewhat, giving appearance of numerous polyps on the colony’s front, when just a very few, scattered, are present; often back of colony without polyps or very few. Curling of polyp body and polyp tentacles gives colony a somewhat lacy look. Color of living colony dark, purplish-red (maroon), deep brick red to nearly black throughout; when placed in alcohol, tends to nearly black. Sclerites warty spindles, generally; those on stem, branches smaller than those on polyps. Largest appear to be those in polyp walls and basal parts of tentacles; large, warty, fusiform, sometimes curved, arranged longitudinally, extending downward in meridional bands to near base of polyps. Smaller spindle-types almost with appearance of a radiate (capstan) shape; some few almost appear as disk spindles. Some few club-shaped sclerites, nearly all of which are the warty, fusiform type. Rods (fingerbiscuit shape) very conspicuous, when present, though not always numerous; generally not heavily warted; most sclerites rich reddish-purple; conspicuous rods vibrant pumpkin orange. The color combination of purple-red and orange is unmistakable.

###### Etymology.

Named in honor of Dr Harry B Torrey from the University of California.

###### Common name.

Dwarf red gorgonian.

###### Distribution.

From MBARI, CAS and Moss Landing Marine Lab (MLML) collection data, found in Monterey Bay (‘Albatross’ stations 4514, 4537, 4546). Range may extend from northern-most end of California Bight to areas off mid-Washington coast (Quinault Canyon, 47°28'59"N, 125°11'45"W; some specimens listed as this species may be *S.pacifica*; further examination required). If specimens, identified as this species off Oregon and Washington coasts, are actually *S.pacifica*, then range for this species from at least Rodriquez Seamount, ~33°59'16"N, 120°59'52"W (west of San Miguel Passage) to Pioneer Seamount (~37°17'44"N, 123°29'58"W) off California; there are ample records at MBARI for sightings of this species in the region between these two seamounts. NMNH records extend the range up through Oregon and Washington, as far north as the mouth of the Strait of Juan de Fuca (48°32'42"N, 124°52'44"W). A recent collection by Olympic Coast National Marine Sanctuary (2008), with specimen collected at ~47°55'16"N, 125°30'00"W, 429 m, was examined, confirming species does extend up into waters of Washington State. As well, one specimen (apparently this species, not examined) was collected from San Diego, Point Loma, at 201–262 m [USNM 49522]. This would put the species in the Bight, but identification may be incorrect. Species most commonly found in the region of Monterey Bay.

###### Biology.

MBARI records would indicate a moderately deep-water form (1,029–2,200 m). It also seems to prefer steep walls of seamounts based on collection details.

###### Remarks.

A brief description is included here as this species is often confused with others in the genus by field investigators, when simply viewing colony morphology in situ (it has been found just north of the California Bight’s upper geographic limit), and completes, to date, descriptions for all colored species within the genus *Swiftia* currently known to exist in the waters along the western North American continent.

In minor ways, previously published descriptions roughly matched that published for *Muricellacomplanata* Wright & Studer, 1889; Harden (1979) listed *M.complanata* as synonymous with this species, and an unpublished Bayer annotation noted: “*Muricellacomplanata* = a *Swiftia*?” Overall, however, descriptions did not match. A brief study of CAS specimens identified by Harden was undertaken, but did not sufficiently clear up his proposed synonymy. For two specimens identified by Harden as this species, one had no locality data; the other was from Monterey Bay. Identifications made by Harden often proved problematic. While definitive specimens with correct identification were needed, was not able to locate specimens with confirmed identification as *Muricellacomplanata* in any of the research collections examined so as to compare known specimens of *Swiftiatorreyi* against it; study of new material, which needs to be collected, is required. As well, Harden (1979: 171, unpublished PhD dissertation) did designate *Psammogorgiatorreyi* Nutting, 1909 (= *Swiftiatorreyi* Nutting, 1909). Cordeiro et al. (2018) does show *P.torreyi* with accepted status, but that as well, *Swiftiatorreyi* is also given accepted status (Cordeiro et al. 2019). Based on a number of descriptions given for members of the genus *Psammogorgia* and Bayer and Deichmann’s (1960) statement regarding the marine province where *Psammogorgia* is likely to be found, specimens examined and studied, identified as *Swiftiatorreyi*, cannot be synonymously identified with any *Psammogorgia* species. At the time that Bayer and Deichmann were working (1960), they suggested that *Psammogorgia* would not/does not occur anywhere outside of the Panamanian province, which then encompassed the area from Cape Blanco, Peru to Lower Baja California, including the Gulf of California (Verrill 1868a, b, c, 1870). As discussed in current literature (Briggs and Bowen 2011), there now exists a California Transition Zone (CTZ), within the Oregon province, extending from Monterey, California to Los Angeles; the California province then extends from Los Angeles to Magdalena Bay, Mexico. Running south of Magdalena Bay around the tip of the Baja California Peninsula, and including all of the Gulf of California, is the Cortez province, with the Panamanian province now extending from the mouth of the Gulf of California to the Gulf of Guayaquil on the border between Ecuador and Peru. Specimens of *Swiftia* described in this work barely make an appearance in upper portions of the California province, but appear no further south, based on a review of all the collection location data for all specimens examined. What is of interest is their appearance in the upper California province, the CTZ and the northeastern Pacific province. In any event, that still definitively puts all specimen/species of the genus *Psammogorgia* outside of the California Bight, either in the Cortez or Panamanian provinces.

Bayer (unpublished annotations) contemplated differences between this species, *S.kofoidi* and *S.pacifica*; his comments do not entirely fit with what has been determined for the species here, and in Nutting’s 1909 description. However, he stated that the species is a “slender form, . . .” whereas *S.kofoidi* is “stouter than *torreyi*. . .” (this can be confirmed). “Similar to *Callistephanus pacificus* Nutting, 1912, pg. 96.” “*P. pacificus* is *Swiftiapacifica*, a brighter red species, with more robust branches, found commonly in waters of Washington and Alaska (bathyal North Pacific). *S.pacifica* is, generally, comparatively more sparsely-branched, with distinctive bar-like sclerites on the anthocodiae and eight-radiates,” than is this species.

Several portions of statements in the discussion section of Breedy et al. (2015: 332) were of interest. Those sections read much the same as several statements this author made regarding the above five species of *Swiftia* in an earlier, pre-revision draft of this volume submitted for review in the spring of 2014. It was interesting to see those comments used as contrast for the new Chilean species that was described.

In the MLML collection, one specimen (C0072) of this species was found; the orange rod sclerite form, generally seen below the tentacles, and anastomosing branches were present (a note furnished with the specimen made a point of the distinctive rods). Two others were labeled as such, but either color was markedly off or, more significantly, branching pattern did not match (no branch anastomoses). Of the MBARI specimens examined, at least six appeared to be this species. Some were originally identified as *S.kofoidi*, but it is fairly certain they are this species; the deep purple-red color is a consistent characteristic, along with many anastomosed branches. As well, presence (or absence) of the vibrant orange rods was a telling feature; if other colonies seen in videos were collected, they should be examined for their sclerites. Overall, colony C0072 has a very distinct deep red-wine color, numerous, dense, thin, anastomosing branches, with polyps having a tendency to curl. In most colonies, a definite front and back is present; the sclerite form that is most evident and obvious in this species is the vibrant orange bacillus-shaped, fingerbiscuit rod, easily seen in a light microscopy array.

#### Further remarks: Consideration of morphological trends, based on geography and possible ecology, of eastern Pacific *Swiftia* species, focusing on those species with colored colonies.

As alluded to in remarks for the description of *Swiftiapacifica*, there appears to be a subtle, yet distinct gradual variation in colony morphology and sclerite form, seen in multiple species of the genus *Swiftia* from the eastern North Pacific, displayed along a geographical and ecological continuum. Historically, the genus *Swiftia* has been assigned variably to the Gorgoniidae, Paramuriceidae and Plexauridae at different times over an historical time frame (Bielschowsky 1918; Deichmann 1936; Bayer 1956; 1961). It is also a genus (particularly so within the context of the eastern Pacific Ocean), that has received limited (and very sporadic) attention (Duchassaing and Michelotti 1864; Verrill 1883 [as *Stenogorgia*]; Wright and Studer 1889 [as *Callistephanus*]; Kükenthal 1924 (*Stenogorgia* synonymy); Deichmann 1936 [*Stenogorgia* = *Swiftia*]; Madsen 1970; Muzik 1979), often resulting in mixed and, at times, confusing species identification. Confusion over identification of species within the genus in the northeastern Pacific stems, in part, from a lack of material, and material collected during widely separated collection events with little or no attempt to look at all pertinent species comprehensively over a wide geographical area. In truth, the presence of the genus *Swiftia* in the eastern North Pacific is an interesting, but, likely multifaceted evolutionary story. Early work that pointed to an explanation for the presence of pertinent species of *Swiftia* in the eastern Pacific began with the work of Madsen (1970). Madsen considered Nutting’s (1912) species, particularly in reference to *S.pacifica*, collected south of the Bering Sea, as a subspecies of the Atlantic species *S.rosea* and concluded that Nutting’s species was an “amphiboreal representative” of *S.rosea*, thus making *S.rosea* an example “of a discontinuous circumboreal octocoral” (Madsen 1970). Thus, the presence of *Swiftia* in the eastern North Pacific could represent a progressive migration of a particular species found in the Atlantic, that has worked its way through waters of the North Pole and ultimately down into eastern Pacific waters. His work also discussed what he considered a subspecies of *S.rosea*, that being *S.roseapallida* (that one or more species, either from the Atlantic or the Pacific, may be related to); Grasshoff (1977) listed the two (*S.rosearose*a and *S.roseapallida*) as two distinct species.

While molecular work (Wirshing et al. 2005; Thoma 2013; Quattrini, pers. comm., Quattrini et al. 2014) has revealed new insights into relationships between *Swiftia* and other genera as well as molecular connection between several species within the genus, both from the Atlantic and the Pacific, in some aspects, supporting Madsen’s (1970) hypothesis, many questions still remain. Molecular work on species in this genus has been undertaken or is currently in progress (M Everett, NOAA affiliate, numerous pers. comm. and Everett et al. 2016); this will certainly shed more light on the origins and dispersal of the genus. The discussion here, however, does not focus on how *Swiftia* member species came to be in the eastern North Pacific, or how they relate to other species in other parts of the world; the focus here is what has occurred morphologically within several member species since the migration and establishment of the genus in the eastern North Pacific. The variations in morphology (both in colony form and color as well as sclerite appearance) are likely to have occurred in response to ecological factors linked to geography.

While working to clarify what species within the genus *Swiftia* were present in the eastern Pacific, it became clear that there was a set of trends in colony appearance, color and sclerite form for *Swiftiakofoidi* (Nutting, 1909), *Swiftiapacifica* (Nutting, 1912) and *Swiftiasimplex* (Nutting, 1909) throughout their distributional ranges in the eastern Pacific, from the Bering Sea, Alaska, USA to upper Baja, Mexico (Isla Cedros). The geomorphological changes that can be seen in *S.koifoidi* and *S.pacifica* (both species forming markedly fan-like colonies) point to one or more of several different hypotheses. These hypotheses are: 1) either these two species are in actuality the same species, with considerable transitional geographic variability seen from south to north within their distributional range (ecological morphs), 2) these two species are distinct species but show high degrees of intermediary form in areas where they overlap (perhaps similar enough to hybridize), 3) these two species are distinct species but display, in the center portion of their range, interesting examples of regional endemism or 4) that both are distinct species, highly subject to varying environmental/ecological parameters, sharing some responses to ecological factors in areas where they live together (factors such as colder water, and depth). In the case of *Swiftiasimplex* (a species that displays a whip-like colony form), it has been determined to be a single species (Everett et al. 2016) but shows geographic and thus perhaps, ecological trends in its sclerite morphology over a vast distance.

**Figure 14.**
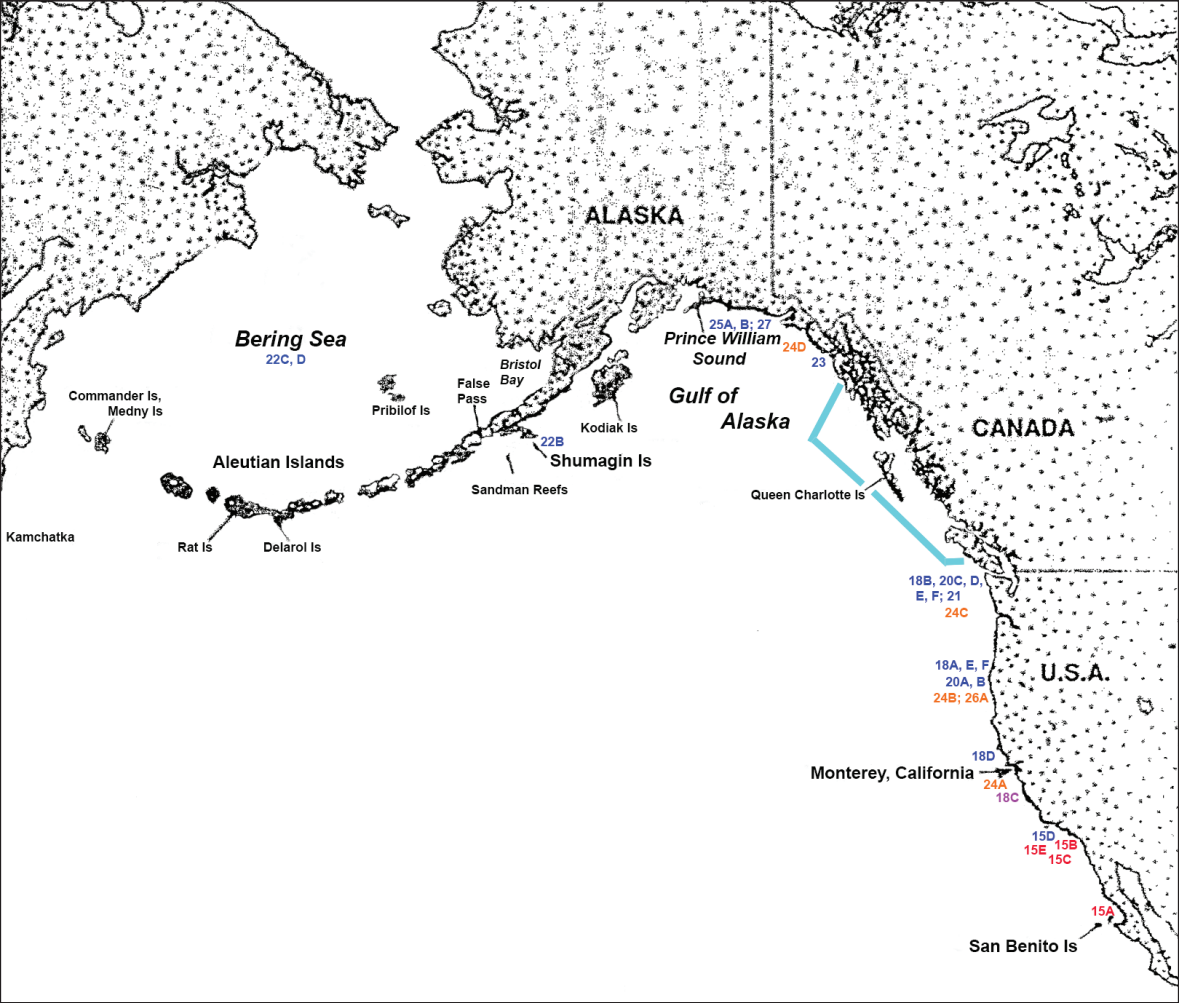
Geographic range of specimens utilized in this study; numbers shown reference Figures 15–27 mentioned in the text, with approximate location delineated by position of the number on the map. Red: *S.kofoidi*, Blue: *S.pacifica*, Fuschia: *S.kofoidi/pacifica* (a specimen decidedly intermediate between the two species), Orange: *S.simplex* and Turquoise: Canadian waters for which specimens are needed.

After examining well over 100 specimens (multiple times over a span of several years (see Appendix 3: List of material examined), transitional variations in colony shape, colony color, branch diameter, polyp distance on branches and in sclerite form became apparent. The geographic range of the specimens examined, and those species discussed here, are shown in Figure 14. Those in the extreme northern end of the range (Bering Sea, Aleutian Islands, Alaska) best show the definitive features of the genus while those furthest south consistently lack some of the key details. The northern-most end of the geographic continuum would be the area into which colonies of the genus first moved in their migration from the northern Atlantic, establishing the origin point for colonies that are now seen further south in this northeastern Pacific Ocean continuum.

**Figure 15.**
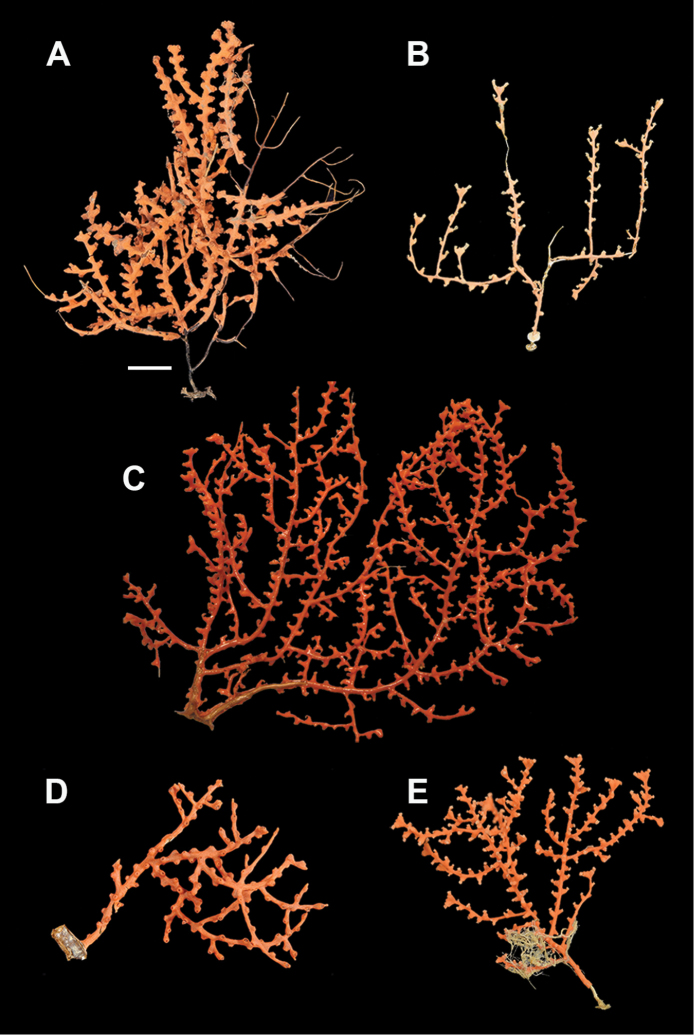
Colonies collected/examined from the southern end of the geographic continuum, identified as *Swiftiakofoidi*. **A**SBMNH 422965; scale bar 1 cm **B**SBMNH 422957; 7 cm H × 10.5 cm W **C**SBMNH 422959; 10 cm H × 10 cm W **D**SBMNH 232036, looking more like *S.pacifica*; 6.5 cm H × 5 cm W **E**SBMNH 422963; 7 cm H × 7.5 cm W.

**Figure 16.**
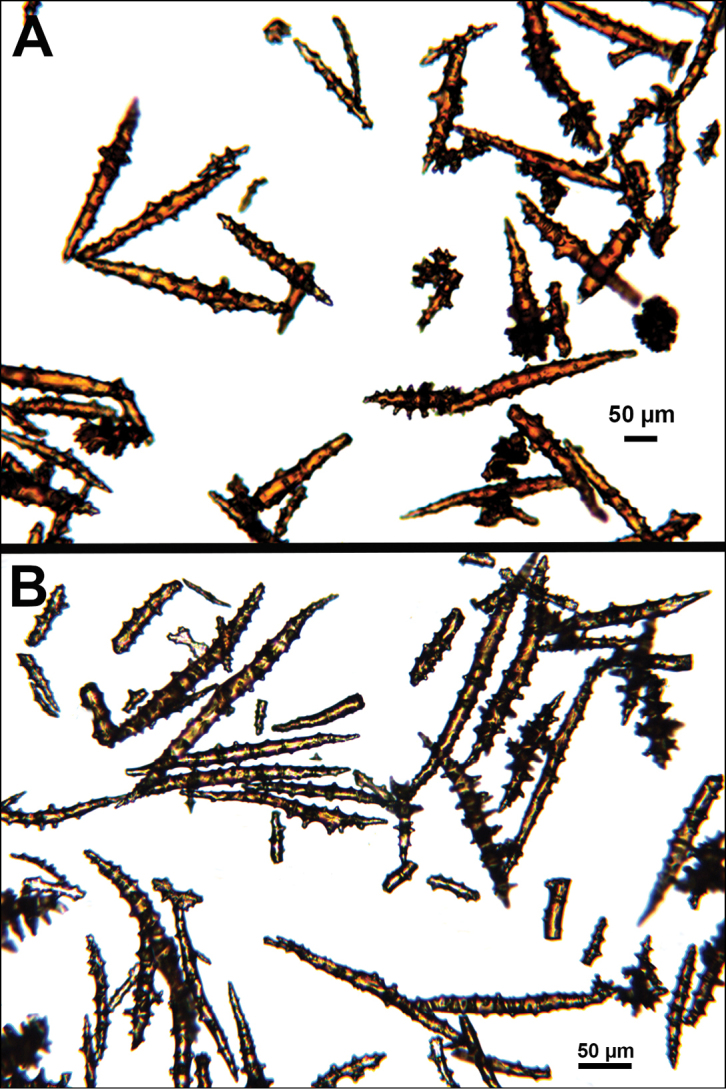
Common sclerite forms seen in colonies collected from the southern end of the geographic continuum, using standard light microscopy. **A** Sclerites taken from SBMNH 422965 **B** Sclerites from SBMNH 232036.

**Figure 17.**
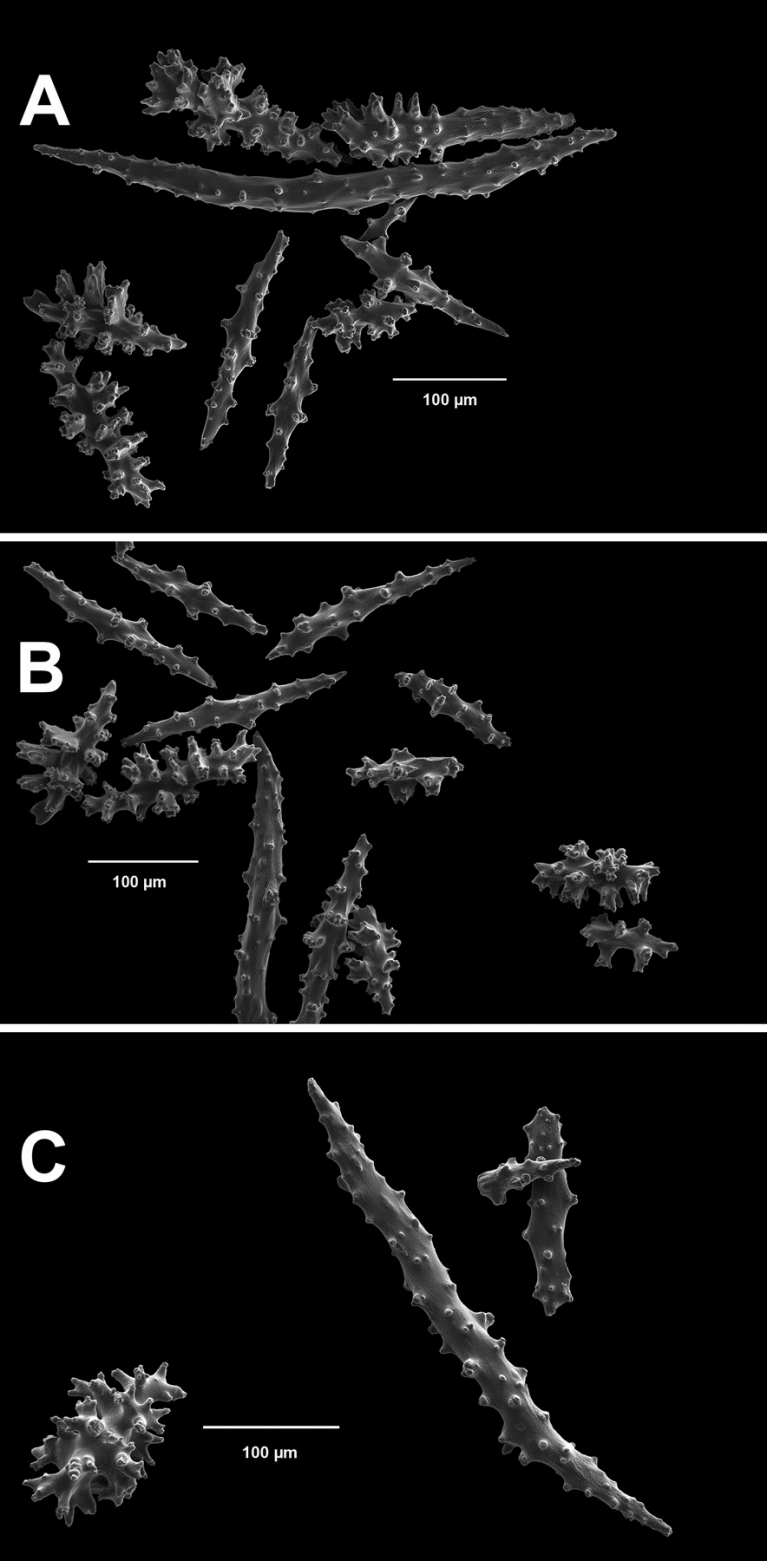
Common sclerite forms seen in colonies collected from the southern end of the geographic continuum, using SEM. **A, B** Sclerites from SBMNH 422965 **C** Sclerites from SBMNH 232036.

The current study of *Swiftia* began with specimens collected at the southern end of this continuum. In the species *Swiftiakofoidi* (the species which appears most commonly in the California Bight based on collection data of specimens examined), colony color is often a vibrant pale salmon-orange (Figures 1, 15), with polyps/tentacles fairly large, often white or a very pale yellow, very widely spaced, giving branches of the colony a distinct “rick rack” profile and overall, a rather delicate appearance, its thin branches rather lacy and open. In colonies collected in either the waters of Baja, Mexico or southern California, very rarely would fingerbiscuit rods be found; sclerite arrays consistently showed a distinct lack of the key sclerite form (referred to as an anthocodial fingerbiscuit rod), but instead the long spindles seen in Figures 2B, 4B would be common, always exceptionally long and thin. As well, numerous shorter, thorny capstan-types, along with a variety of other odd sclerite shapes (such as a torch) in far smaller numbers (Figures 16, 17) were often seen. Of interest is the colony shown in Figure 3, having the general appearance of *S.pacifica*, but collected in southern California, displaying very long, thin spindles and very few fingerbiscuit rods (Figure 4C).

As specimens collected in the vicinity of the CA Bight’s northern edge were examined (including specimens collected above Point Conception), the slender spindles and shorter, thorny capstans were still displayed, but on occasion there would be a few sclerites that nearly matched the key sclerite form of the genus (the fingerbiscuit rod), but were usually longer than expected (best described as a “Cheetos” cheese puff), as seen in Figures 19, 20. These latter, however, were by no means common; many colonies identified as *S.kofoidi* did not display them.

**Figure 18.**
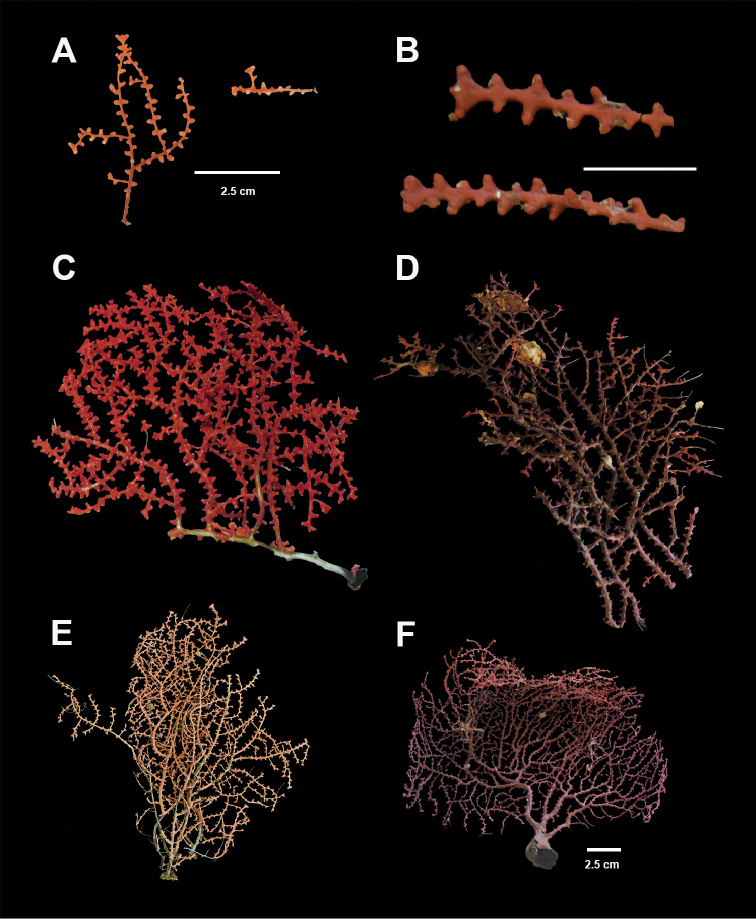
Colonies collected/examined from the central portion of the geographic continuum, ranging from roughly Monterey County, CA to southern Washington State. **A**LACoMNH (NOAA #CB 34019) **B**NOAA FRAM #100220840; scale bar 1 cm **C**MLML #C0072; size not determined **D**NOAA FRAM #100112080 (CB 34406-040); 11.5 cm H × 6.4 cm W **E**NOAA #CB 50003-008; 16.5 cm H × 12.6 cm W **F**LACoMNH (NOAA #CB 34010); 15 cm H × 19 cm W.

**Figure 19.**
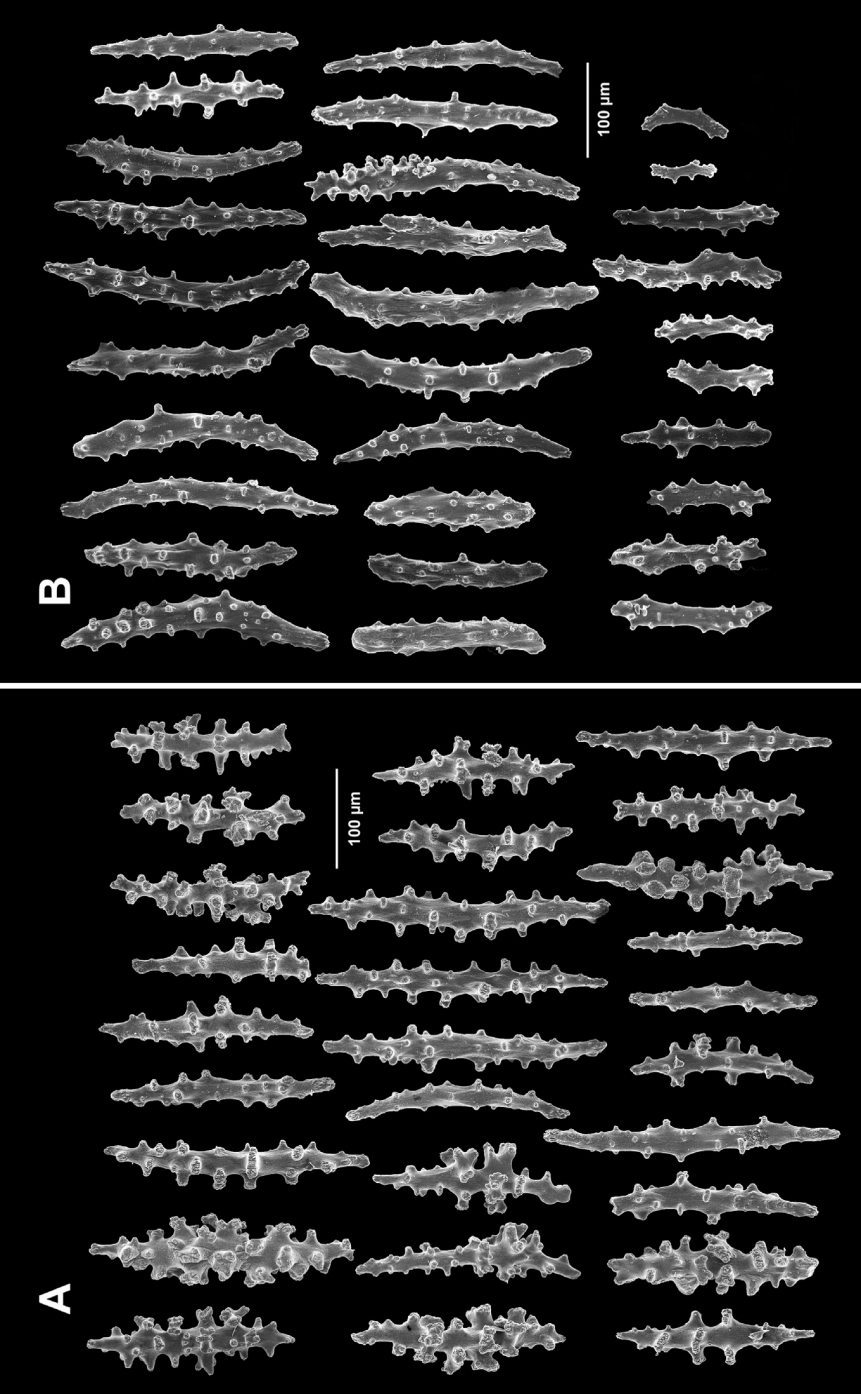
Variation in common sclerite forms seen in colonies from the central portion of the continuum, in SEM. **A** Coenenchymal sclerites from NOAA FRAM #100112080 (CB 34406-040) **B** Polyp and tentacular sclerites from the same specimen. SEM images taken by Carla Stehr (NOAA), provided by Ewann Berntson (NOAA).

**Figure 20.**
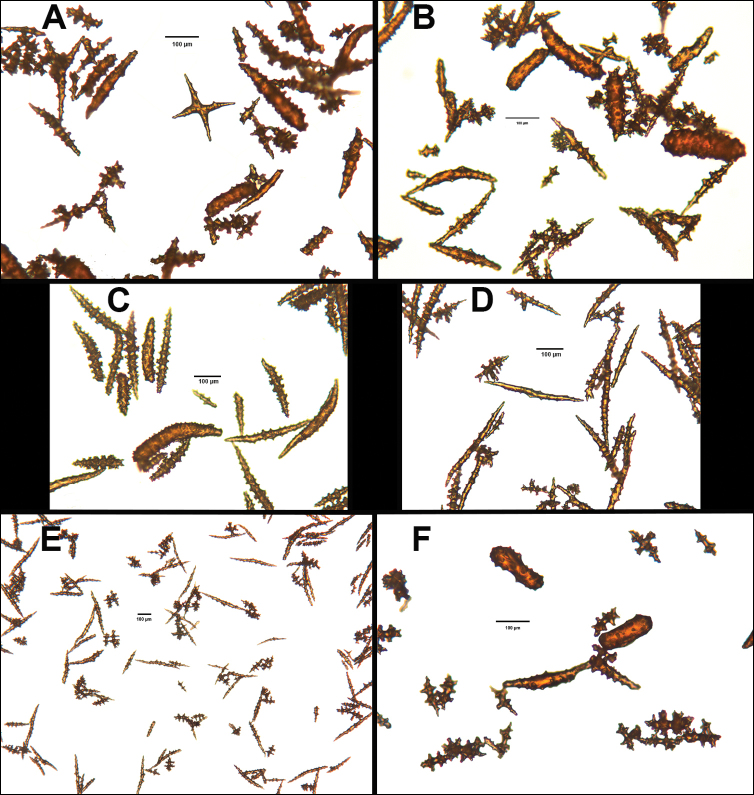
Common sclerite forms seen in colonies collected from the central portion of the geographic continuum, using standard light microscopy. **A, B**NOAA FRAM #100112070 **C**NOAA FRAM #100220840 **D**NOAA #CB 50001-004 **E** OCNMS #EPI 202 **F** OCNMS #EPI/SUC 216.

As specimens collected even further north were examined (along the coasts of Oregon, Washington and on up to Alaska), colony appearance was more and more as that seen in Figure 3, but the long spindles became shorter and shorter in specimens collected further and further north, while the fingerbiscuit rods became more and more common, obvious and larger (Figures 5A, 6A, C, D). Off the central coast of Oregon to the central portion of Washington State, the colonies looked more and more like *S.pacifica* (occasional colonies looking like *S.kofoidi* were found, however; Figure 18), but there was a marked transition (tendency to being shorter) in the appearance of long spindles while fingerbiscuit rods became more and more obvious. Again, it appeared as though *S.kofoidi* and *S.pacifica* might be: 1) two colony morphs of the same species, with “transitional” sclerite appearance (long or short spindles in some combination with presence or near absence of fingerbiscuit rods) or 2) interbreeding (two morphs of the same species) but equally 3) could be displaying degrees of hybridization between two very similar, but different species.

**Figure 21.**
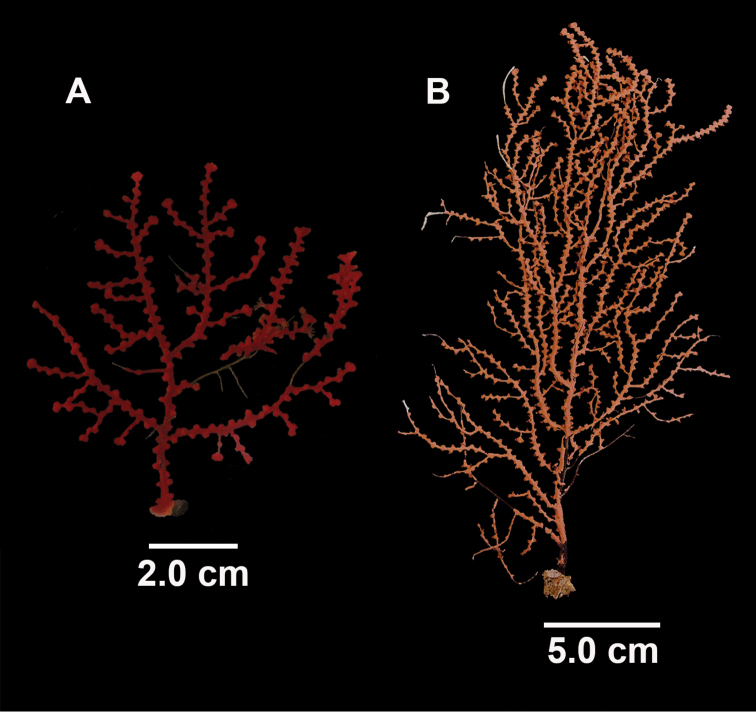
Colonies collected/examined from the northern end of the geographic continuum, confirmed identification as *Swiftiapacifica*. **A** OCNMS #OC06_0531, EPI 127 **B**NOAA 41-39-1 (AB17-0010). Image of specimen shown in A provided by Mary Brancato (OCNMS); specimen shown in B provided by Robert Stone (NOAA).

For specimens identified as *Swiftiapacifica*, the species appeared to be far more common the further north in location specimens were collected from. Only on rarest occasions did a colony reveal itself as a specimen of this species in the CA Bight (Figure 15D); it was far more common north of the CA Bight, on up through waters off Oregon, Washington and Alaska (Figures 18, 21). While unable to examine specimens collected off of Canada and its associated islands, it is hard to imagine that it would not be found in the waters of that region.

**Figure 22.**
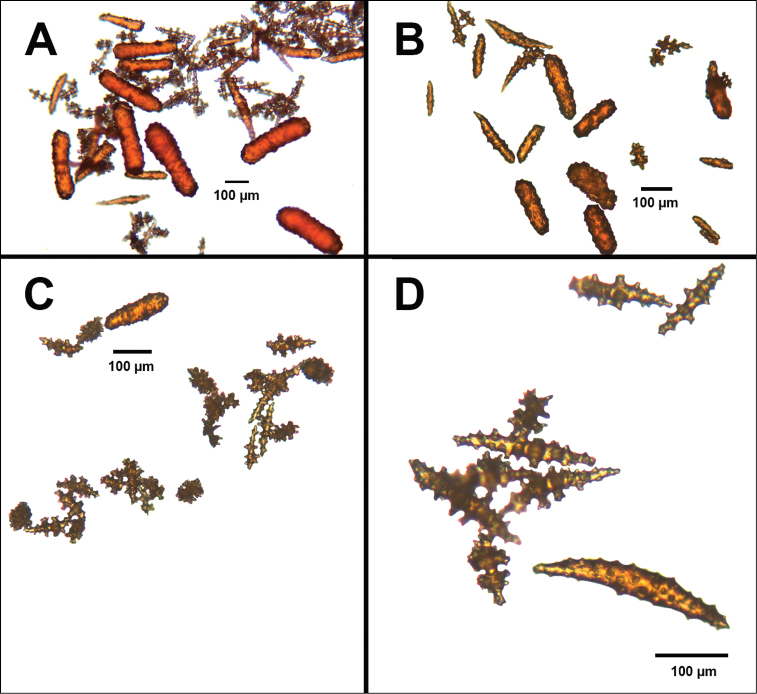
Common sclerite forms seen in colonies of *S.pacifica* collected from the northern portion of the geographic continuum, using standard light microscopy. **A**NOAA #41-39-1 (AB17-0010) **B**NOAA #CB 50003-021 **C, D**NOAA #CB 50003-032.

**Figure 23.**
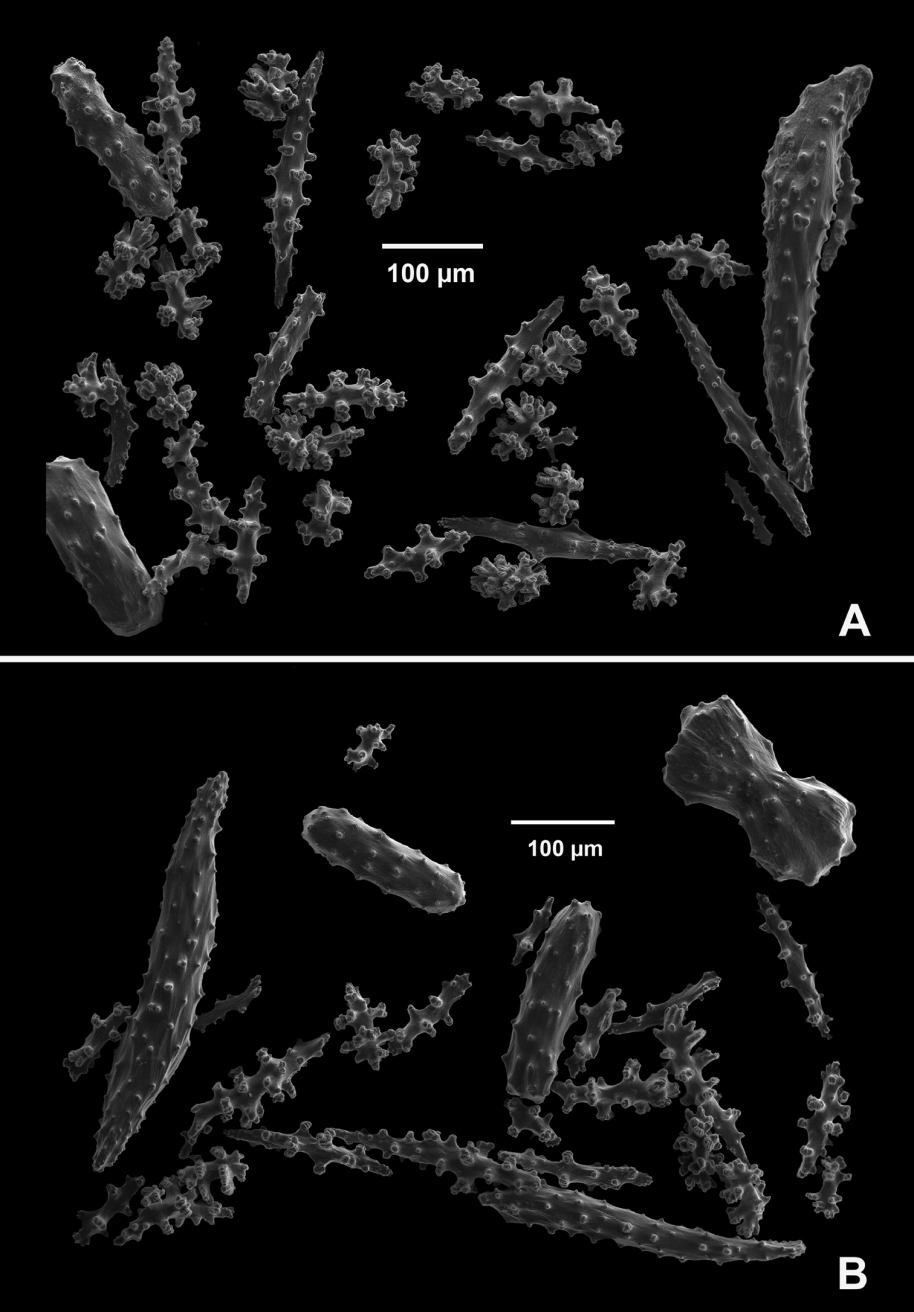
Sclerites from a specimen collected in the northern portion of the geographic continuum, in SEM. **A, B** From NOAA #41-100A-2 (AB17-0009).

In terms of *S.pacifica*’s overall appearance in the eastern Pacific, the colony was often (but not always) more robust, being thicker-branched and bulky in overall appearance (Figures 3, 15D, 18A, B, C, 21A), the color more commonly a deeper crimson red to brick-red (often with a grey or green tinge); polyps were a bit smaller (typically more boxy), and often (not always) much more closely situated next to each other, but not as closely situated as polyps seen in the species *S.torreyi* (Nutting, 1909); polyp/tentacle color usually a slightly darker version of the colony coenenchyme. However, throughout its range (Oregon, Washington, even in the far northern parts of its range, such as Alaska, the Bering Sea, Aleutian Islands), there were specimens that looked in overall appearance much more like the delicate colony form of *S.kofoidi* (yet color more in keeping with the darker red shades; for example Figures 18D, F, 21B). In some few of these latter specimens, in sclerite arrays, a lack of the fingerbiscuit rod could be found, though this was a fairly rare event (Figure 22C, D). However, the farther north a specimen was collected, the fingerbiscuit rods would be visible, obvious, with a vibrant orange to pumpkin color (Figures 20B, F, 22A, B, 23A, B). This sclerite form is characteristic in specimens identified as this species (Nutting, 1912). Additionally, long spindles became less and less numerous; those present were slender but displayed far shorter length than those seen in *S.kofoidi*. With specimens collected in waters off northern Washington to the Bering Sea, Alaska, the colonies very much looked like that of *S.pacifica*, a very deep gray-red, with much shorter long, thin spindles and very evident fingerbiscuit rods, as shown in Figures 5, 6.

In summary, at the extreme ends of the range (Baja and southern California vs. Bering Sea, Alaska), there is, in the south, the appearance of a delicate and bright orange colony and in the north the appearance of a thicker, denser colony of a deep red to gray-red. This could lead to the conclusion that there are two separate species, even as specimens found in the middle of the range showed an interesting mix of colony morphology, colony color and presence or absence of certain sclerite forms.

Morphologically at least, it would seem that *S.kofoidi* and *S.pacifica* are separate species. However, it was difficult to clearly see, as specimens from areas intermediary in the range were examined and considered, that they were separate species. It is these intermediate mixes of features in the areas of both Washington and Oregon, to the shores of the northern CA Bight boundary that are of most interest. Specimens from Oregon or northern California could look more like *S.kofoidi* in colony shape, but color was off, or there were hints of something that resembled a fingerbiscuit rod. Specimens from Washington or Oregon could look far more like *S.pacifica* in color and colony form, but sclerite arrays revealed what had been seen in arrays of sclerites from specimens clearly identified as *S.kofoidi*. Yet, overall, the sclerites labeled as “Cheeto-type” or those called the fingerbiscuit rods, became more and more common in specimens, the further north the specimens were collected.

**Figure 24.**
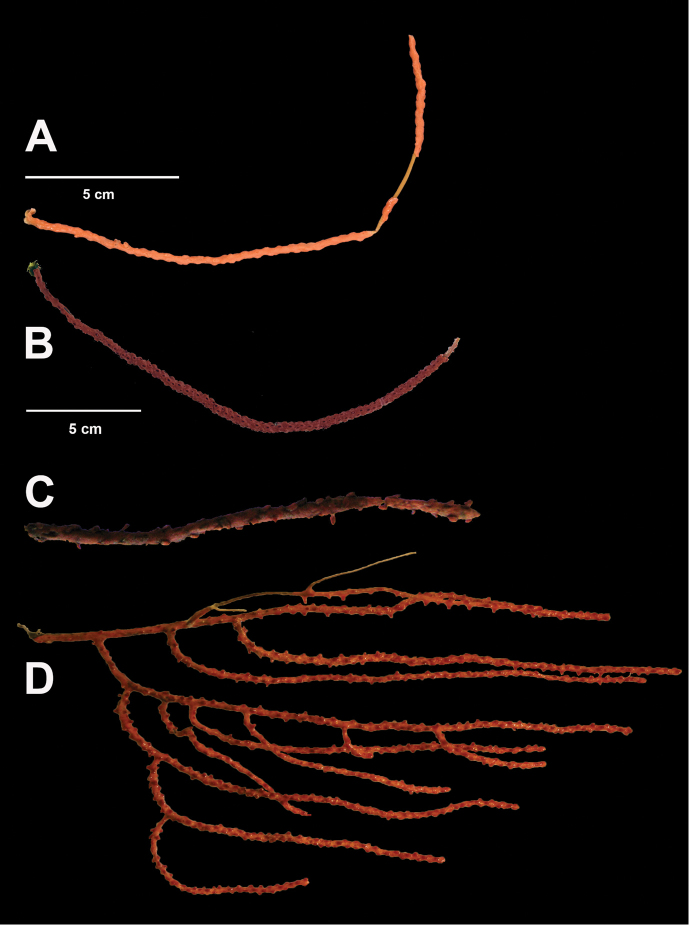
Colonies collected/examined from throughout the geographic continuum (shown **A–D** from south to north), identified as *Swiftiasimplex*. **A**SBMNH 422979 **B**NOAA #CB 34013 **C**NOAA #CB 34212-039 **D**NOAA CRW_3636 8; 35 cm H × 17–20 cm W. Image **C** Courtesy of Ewann Berntson (NOAA, WA); image **D** Courtesy of Robert Stone (NOAA, AK).

**Figure 25.**
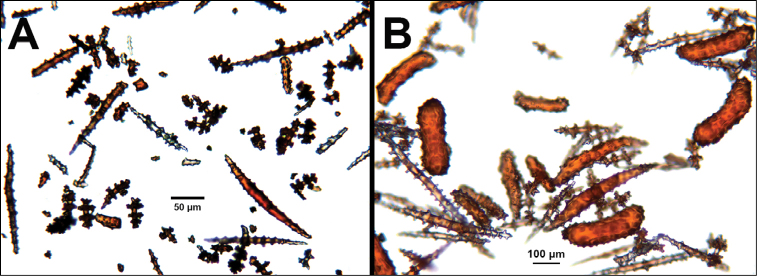
Sclerite arrays of *S.simplex* seen using light microscopy. **A**SBMNH 422979 **B**NOAA 81-99B-1 (AB12-0127).

**Figure 26.**
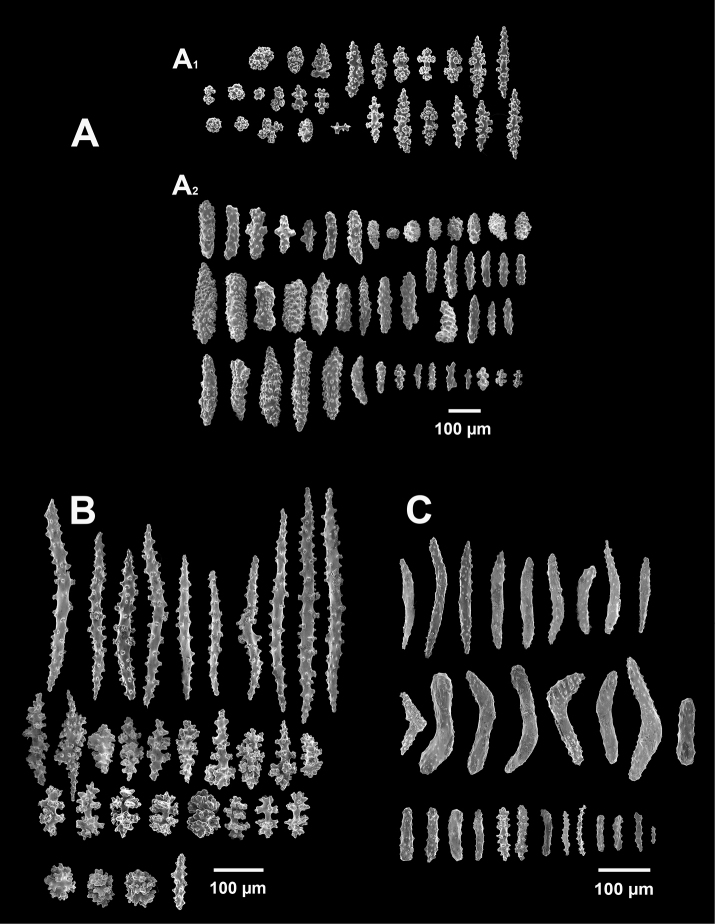
Sclerites of *S.simplex* in SEM. **A**NOAA #CB 34011; **A_1_**-sclerites from coenenchyme, **A_2_**-sclerites from polyp tentacles **B**NOAA #CB 34212-039, primarily coenenchymal sclerites **C** Also from #CB 34212-039, primarily sclerites from the polyp. All SEM images in this figure prepared by Carla Stehr (NOAA), provided by Ewann Berntson (NOAA).

**Figure 27.**
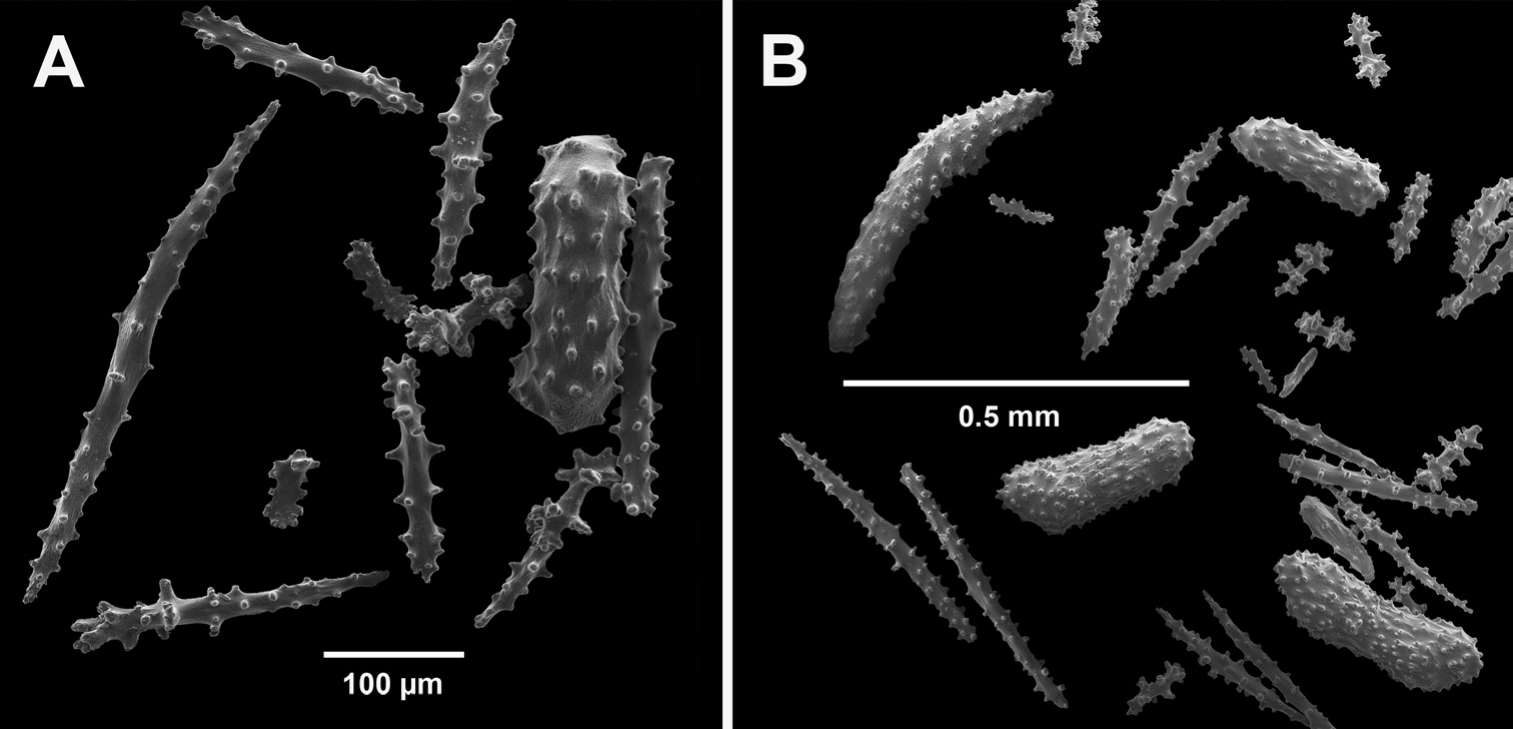
Further sclerite arrays in SEM, for a specimen from the far northern end of the continuum. **A, B** From NOAA #81-99B-1 (AB12-0127).

*Swiftiasimplex* revealed itself, morphologically, to be a single species, but within the species, as specimens were examined (following collection along the south-to-north continuum), while colony morphology (usually a single or rarely, minimally-branched stem) and color (a pinkish, dirty, brick-red), remained consistent (Figures 7, 8, 24), specimens in areas further north always displayed the fingerbiscuit rod (Figures 25B, 26, 27) while those in more southerly locations lacked the fingerbiscuit rod sclerite (the change in the appearance of this sclerite form, present to absent, appeared roughly in the vicinity of the northern edge of the CA Bight), but lack of fingerbiscuit rods could be confirmed as far north as Monterey Bay in some specimens; an example is SBMNH 422979 (See Figure 25A).

Are *S.kofoidi* and *S.pacifica* two separate species? Based on colony form, coenenchyme color, polyp color, size and placement, it would seem so. If so, are they similar enough in genetic makeup to be able to readily hybridize? Based on what has been related here in terms of morphological aspects, this might be a viable possibility, especially in the waters off Washington and Oregon down to the area of the northern CA Bight. Preliminary molecular work (M Everett, NOAA affiliate, personal communications) might lead to such a conclusion. However, the sclerite arrays demonstrated by both species turned out to be less clear. Generally, more southern-collected species lacked any hint of the fingerbiscuit rod. The further north a specimen was collected, the more likely the fingerbiscuit rods were to be seen. But, there were many exceptions to this generality. The general trend could mean an ecological response: thicker, shorter fingerbiscuit rods in the colder waters of the northern part of the total range (where food may be more abundant and energy to generate thicker and more forms of sclerite is available), and thinner, more slender spindles, but not the formation of rods, in the warmer to moderate temperate waters in the southern part of the total range (where food supplies may be more variable and/or less abundant, thus less energy available for extensive sclerite formation). In support of this hypothesis, two specimens (of an, as yet, undescribed species) of *Swiftia* in New Zealand’s National Institute of Water and Atmospheric Research, Ltd. (NIWA) Research Collection (not yet cataloged, but with the following identification numbers: the first, NZ01, Stn Z11059, Stn KAH0204/32, collected 17 April 2002, 780–810 m [wet] and the second, U582 [wet]) were examined, from waters offshore, northeast side of North Island, New Zealand, in which, throughout the entire colony, the only sclerite form to appear in multiple sclerite preparations undertaken were the fingerbiscuit rods. Is this sclerite form then a response to depth and/or variable temperatures? For *S.kofoidi* and *S.pacifica*, the intermediate, and variable, mix of sclerites in the intermediate region of the south-to-north continuum along the continental western US coast could represent responses to variable environmental conditions, prevalent in the vicinity of the western US coast, where major currents running through this area wax and wane throughout the seasons and years, subject to storm vagaries, etc.

Are the two species actually subspecies or ecological morphs of one species? (In this case, a case could be made that the one species retain the name *Swiftiapacifica*.) Molecular studies done by M Everett (NOAA affiliate, Port Orchard, WA) seemed to indicate a close affinity of the current two species. Based on the morphological studies reported here, especially with regards to those found in a wide central, intermediate area of the range (from northern California to the central-northern Washington coast), where there was wide variation and a mix of features in the specimens, especially with regards to the mix of sclerites seen in those specimens, the idea of the two really representing a wide array of ecological response in one species, perhaps to some shared ecological feature, is not outside the realm of possibility. There is however, an alternative hypothesis: presence of many regional endemic species, each with its own set of parameters, chosen from the array of features discussed here; this may require the need for further species designations for each endemic form, if indeed, they exist. More specimens need to be collected in the near future, with intentional effort made to hone in on specific areas within the north-to-south continuum, most notably in Canadian waters and in the “transitional, intermediate zone” of the continuum (Point Arguello to Point Conception, CA), to explore this conjecture. Molecular work being undertaken by M Everett and her lab now, and in future (especially should new collection events occur), will further clarify some of this.

As for *S.simplex*, it is a less complicated situation. In the southern portion of its range, where there is a tendency not to produce fingerbiscuit rods, the condition could be an ecological response to some environmental factor (be it temperature or food supplies, as examples) while the appearance of the fingerbiscuit rods, consistent with collection locations further north, in much deeper/colder water, equally could be an ecological response to those colder water conditions. Based on the work of Everett et al. (2016), there is a high level of gene flow within this single species throughout its range along the west coast of the United States.

In conclusion, differences in colony size, shape, branch diameter, polyp placement on branches, and color as well as presence or absence of key sclerite forms was obvious. These differences have generated degrees of confusion as to species identification along that geographical gradient. As those differences are considered, the conclusion could be drawn that the differences reflect ecological conditions and colony responses to them. They could, however, also lead to the assignment of distinctly different colony forms as different, but yet remarkably similar, species. Conversely, all colonies along the geographic range could actually be representatives, in a single species, of a high degree of variability in response to varying ecological situations. More work needs to be done to categorically determine whether *S.pacifica* (in its transition down the western coast of North America into southern California) is a single species or whether it has developed into a different species, represented as *S.kofoidi*, below the California Bight’s northern boundary. Further morphological study, intimately tied to molecular examinations, could help to further clarify the mechanisms (ecological or otherwise) behind the visible morphological/structural transitions seen throughout the geographic continuum discussed here, and aide in the confirmation of either separate species (*S.kofoidi* and *S.pacifica*) or a single, highly flexible and variable species that represents the eastern Pacific Ocean extension of the nominant Atlantic species, *Swiftiarosea*. For the present, reference is made to *S.kofoidi* and *S.pacifica* as separate, but closely related, species.

##### Swiftia pusilla

Taxon classificationAnimaliaAlcyonaceaPrimnoidae

(Nutting, 1909)

Eumuricea pusilla (Nutting, 1909): 718, 719; pl 88 (figs 3, 4). Kükenthal 1924: 152.
Swiftia pusilla (Nutting, 1909): comb. nov. Breedy and Guzmán 2015: 22, 23.

###### Material examined.

No material in the SBMNH collection (see Appendix 3: List of material examined).

###### Diagnosis.

Colonies likely small; branching presumed irregular; with material available, not possible to confirm plane configuration; may present only a few branches or is unbranched. A main stem could give rise to roughly alternate branches, at irregular intervals. Main stem and branches may tend to curve upwards, almost running parallel to one another; stem and branches with nearly same diameter; branches can be slightly swollen. Polyps on all sides of branches, fairly dense, roughly arising off branch surface at right angles; occasionally slanting, bending upwards; may give appearance of biserial rows, but often not distinct, usually sitting on opposite sides of branch. Polyps vertically placed, conical and prominent, perhaps slightly raised; distal-most end somewhat widened, showing eight-rayed figure in retraction. Anthocodiae appear to retract vertically into truncated tips, with polyps completely able to retract tentacles. Very few sclerites that could be extracted were generally sharp, acute needles (spindles). Present in coenenchyme (relatively thin) of polyps, coarse spindles; many unsymmetrical spindles bearing crenulated warts, jagged edges and processes. Marginal sclerites tending to converge as eight calycular processes, tips projecting more or less distinctly. In polyp body walls, spindles may be partially overlapping, transverse in orientation; not arranged in convergent double-rows. No presence of any fingerbiscuit rods could be detected. Colonies (when live?) colored in shades of brown; faded to gray or white with time (preservatives).

###### Type locality.

**Holotype** USA, California, San Diego County, San Diego, Point Loma, 176 meters.

###### Type specimen.

**Holotype** NMNH 25430 [wet/dry]; all material was examined (as well as could be done), several times.

###### Remarks.

Examination of preserved material at NMNH, both wet and dry, was not at all enlightening. Specimens very small; wet material in very bad shape, due to protracted storage in formalin (while now water washed and placed in 70% ETOH, the damage had already been done, long ago). The dry fragment was very small, thin and whitish, with zig-zag appearance. This correlated with photographs shown in Nutting’s (1909) work. No other institution, where collections were examined, had any material with this species designation. Nutting’s (1909) description of the colony, being more or less flabellate and in one plane, does not negate the possibility of his specimen being in the genus *Swiftia* (appearance of polyps on branches is similar), but there is doubt as to whether this is a separate species; specimens in question may be badly preserved or bleached examples of something else. In general, appearance of fragments most closely resembled a species of *Thesea* seen in southern California waters; coloring, however, does not match most *Thesea* (fragments bleached?) and any species in the genus *Thesea* should have the distinctly large, spheroidal sclerite form (not seen in this specimen, but minimal material available to work with, highly degraded). Or it may be a species belonging to genera that can display long, thread-like colonies, such as *Leptogorgia*, *Eugorgia* (new species described in this work, Part II) or even an aberrant, bleached *Swiftia*. The notion that this colony form, described by Nutting, is not an accurately named species, or even a member of the genus *Eumuricea*, has support in final comments made by Kükenthal (1924), translated here: “(i)n no case does this form belong to *Eumuricea*, arguing against it in comparison is the overall construction (shape), the arrangement of the polyps in two lateral rows, their wide distance from one another, as well as the form of the coenenchymal sclerites.” This means that Nutting’s material at NMNH does not belong in the genus *Eumuricea*; unfortunately, with the material in such poor condition, it may never be possible to clearly confirm what genus and species the specimens do belong to. As Breedy and Guzmán (2015) have elected to place it in the genus *Swiftia*, Kükenthal’s comments are supported. Notably, no mention of this species is made in the WoRMS Database listing of accepted species in the genus *Swiftia*.

SBMNH has several lots (provided by both OCSD and LACSD) in its possession that closely resemble the fragments held by NMNH. They are without color (white) or very, very pale yellow, and show the polyp pattern seen in Breedy and Guzmán (2015: fig 11). However, they also very closely resemble, in branch form (diameter, polyp placement), some sclerites (in the predominance of longish spindles, and the unsymmetrical sclerites with jagged edges), and the dull coloring, the specimens that many field investigators in southern California are calling a paler, less common species of *Thesea*. The fragments shown in Nutting (1909: figs 3, 4), and those shown in Breedy and Guzmán (2015: fig. 11A), closely resemble what is seen with local, somewhat less abundant, specimens of a species of *Thesea*. In sclerite examinations of these paler, less common, “*Thesea*-like” specimens, they do not clearly match the sclerite forms that are seen in species of *Swiftia*, and while they come closer in matching the sclerites seen in the commonly encountered *Thesea* species, they do not exactly match those sclerites either. Based on that seen in numerous examinations of both locally collected specimens of both *Swiftia* and *Thesea*, encompassing a number of species, the suggestion would be that Nutting’s *Eumuriceapusilla* might be a species of *Thesea* rather than a *Swiftia*. Nothing can be certain until more specimens that fit his original description can be located, collected and studied. With *Eumuricearigida* having been recently assigned to the genus *Thesea* (Ofwegen, 2014), there is a likely possibility that S. (E.) pusilla might need to likewise be assigned to the genus *Thesea*.

##### Genus Thesea

Taxon classificationAnimaliaAlcyonaceaPlexauridae

Duchassaing & Michelotti, 1860

Thesea = Acis (non Acis, Billberg, 1820, Lesson 1830) Duchassaing & Michelotti, 1860: 18, 19; 1864: 12–14. Kölliker 1865: 136. [Thesea. = Acis (pars) Wright and Studer 1889: 56.
Acis Kükenthal, 1919: 836.
Thesea Duchassaing & Michelotti, 1860: 18, 19. Nutting 1912: 80. Kükenthal 1924: 153, 154. Deichmann 1936: 110–112. Bayer 1956: F206-F207; 1958: 50; 1981: 945.
non Thesea Nutting, 1910a: 50 {= Placogorgia}. Riess 1929: 401 [= Scleracis: see Deichmann, 1936: 111].
non Elasma (non E. Jaennicke, 1866); Studer (and Wright) 1887: 58.
non Elasmogorgia Wright & Studer, 1889: 132. Hickson 1905: 814. Nutting 1909: 717 (California = Thesea). Thomson and Simpson 1909: 238. Nutting 1910a: 45. Thomson and Russell 1910: 159. Nutting 1912: 85. Kükenthal 1919: 752, 836; 1924 (pars): 148]. Thomson and Dean 1931: 199. Matsumoto and Ofwegen 2016: 4.
Evacis (nomen nudum) Verrill, 1912: 373, 377 [Des. Deichmann, 1936].
Euacis Aurivillius, 1931: 126.
Filigella Gray, 1868: 443. Kinoshita (pars) 1909: 1. Verrill 1912: 389. Kükenthal 1919: 762, 844. Aurivillius 1931: 126–129. Deichmann 1936: 147. Bayer 1956: F206. Muzik 1979: 142, 143; Matsumoto and Ofwegen 2016: 2, 16, 19.
non Filigella = Elasmogorgia Kinoshita, 1909: 1, 4, 5. Kükenthal 1924: 148.
Heterogorgia (pars) Verrill, 1868c: 413. Nutting 1910a: 87.

###### Type species.

*Theseaexserta* Duchassaing & Michelotti, 1860 (non *Gorgoniaexserta* Ellis & Solander, 1786) = *Theseaguadalupensis* Duchassaing & Michelotti, 1864.

###### Diagnosis.

Colonies moderately threadlike, some (rare) sparsely branched in one plane; slightly flexible branches slender, long, each ascending to slightly expanded, stout, possibly truncated, distal branch tip; terminates with flattened arrow-head-like tip; proximal end, when free, also drawn into arrow-head (looking as distal end), or with small attachment disk; axis horny; coenenchyme thin. Calyces distinct, roughly placed alternately; low-domed with eight marginal teeth formed by simple converging spindles. Sclerites of coenenchyme in two layers: outer one containing large, spheroidal/oval or plate-like bodies, outer faces of which are commonly undulated, generating a wash-board appearance (key sclerite form for genus); inner layer including warted spindles of smaller diameter.

###### Remarks.

Kinoshita (1909) and Aurivillius (1931) considered the genus *Filigella* synonymous with *Elasmogorgia*. This synonymy was called into question by Matsumoto and Ofwegen (2016) in statements made regarding two distinct species. They stated that *Filigellamitsukurii* is actually *Euplexauramitsukurii* and that there is only one species in the genus *Elasmogorgia*, that being *Elasmogorgiafiliformis* Wright & Studer, 1889 (closely resembling a species in the genus *Astrogorgia* Verrill, 1868); in actuality, there now are three accepted species in this genus, listed accordingly by Cordeiro et al. (2019) in the WoRMS Database. Considering the characteristics of the two genera (*Euplexaura* Verrill, 1869 and *Elasmogorgia*), neither *E.mitsukurii* or *E.filiformis* belong in the genus *Thesea*; thus *Elasmogorgia* is not synonymous with *Thesea*. However, Matsumoto and Ofwegen (2016) did state that the genus *Filigella* is a synonym of *Thesea*. The basis for this might be the fact that the genus descriptions given by Bayer (1956a) for *Thesea* and *Filigella* overlap, in part; this would explain the suggestion of synonymy between the two genera made by Bayer in 1958. Bayer (1958) synonymized the two genera *Filigella* Gray and *Elasmogorgia* Wright and Studer with the West Atlantic genus *Thesea* Duchassaing & Michellotti, and transferred the genus to the family Plexauridae from the family Paramuriceidae (latter no longer a currently recognized taxon). Bayer (1981) then stated that *Filigella* was a synonym of *Thesea*; based on the recent work of Matsumoto and Ofwegen (2016), *Elasmogorgia* must be removed from Bayer’s 1958 synonymy, while *Filigella*’s synonymy might be retained. According to Utinomi (1961) however, the coenenchyme of *Filigella* is thinner and less distinctly displays the two layers seen in most plexaurids; as well, anthocodial armature is more powerful so as to form an operculum, typical of the group formerly known as the paramuriceids. Based on this, he considered it better to retain the genus name *Filigella* than to unite it with the plexaurid genus *Thesea*. Muzik (1979) also did not synonymize *Thesea* with *Filigella*; her rationale was that *Filigella* had a distinct collaret (having something more like a true operculum), along with numerous scales forming the tentacle backs. While *Filigella* was considered to be similar to *Thesea*, *Thesea* was stated to have “bulky boots” (Muzik, 1979: 143) forming its (collaret’s) points. She went on to surmise that, “depending on the importance of anthocodial armature, these three genera *(Thesea, Filigella*, along with *Muricella)* may remain distinct or one day be merged into one genus.” The NMNH did not use/recognize *Filigella* during times when author visited and worked in the collection; *Thesea* was the genus designation used. From examinations of specimens at NMNH, etc., noting specimen identification while also considering the synonymy discussion given here, *Thesea* and *Filigella* may or may not be synonymous; the genus *Thesea* is used here for colonies from California (and Mexican) waters. *Elasmogorgia* (and its species, including *E.filiformis*) is not considered, based on the recent work of Matsumoto and Ofwegen (2016). Cordeiro et al. (2019), in the WoRMS Database, indicated the genus *Thesea* as having accepted status, and they list some twenty-eight species within the genus; however, neither *Thesea* [non *Elasmogorgia*] *filiformis* (Nutting, 1909) or *Theseavariabilis* (Studer, 1894) are included in that listing.

##### Thesea

Taxon classificationAnimaliaAlcyonaceaPlexauridae

spp. (one or more unidentified species)

**Figure 28.**
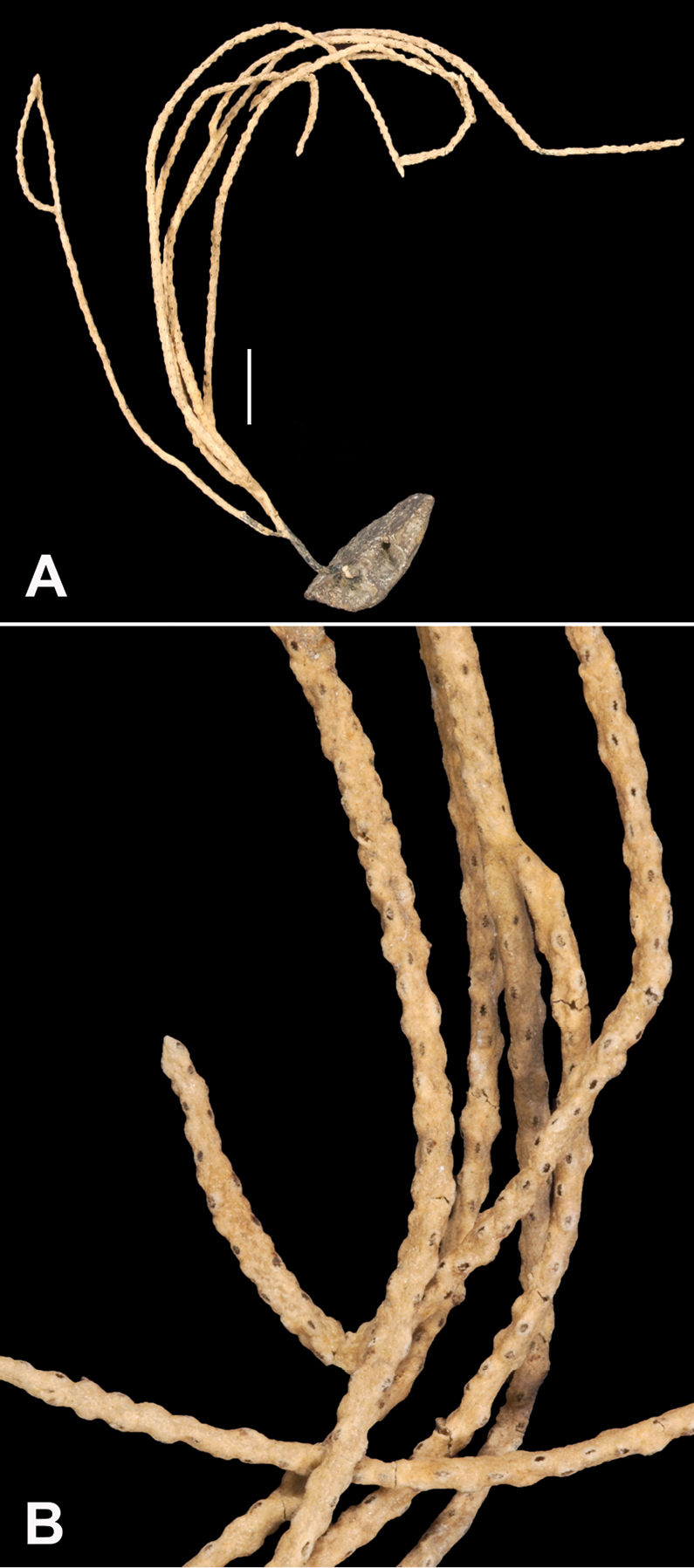
*Thesea*, spp., SBMNH 422414. **A** Colony 23 cm from base attachment point on rock to tip (attachment was very tenuous; rock now separated from colony) **B** Closer view of branches, branch tip and pattern of calyces on branch surface.

**Figure 29.**
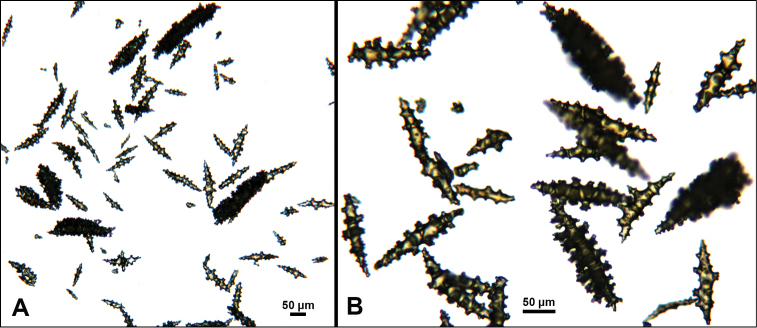
*Thesea* spp., SBMNH 422414. **A** Light microscopy sclerite array, 4× magnification, showing different sclerite forms seen in specimens of *Thesea*; large, dense sclerites characteristic for members of the genus **B** 10× magnification, illustrating not only dense sclerite form, but also common spindle form. Very densely warted sclerites range from 317–450 µm, slightly shorter, dense spindles ~220 µm, and those sclerites that are thinner, less warted measure 190–200 µm in length.

**Figure 30.**
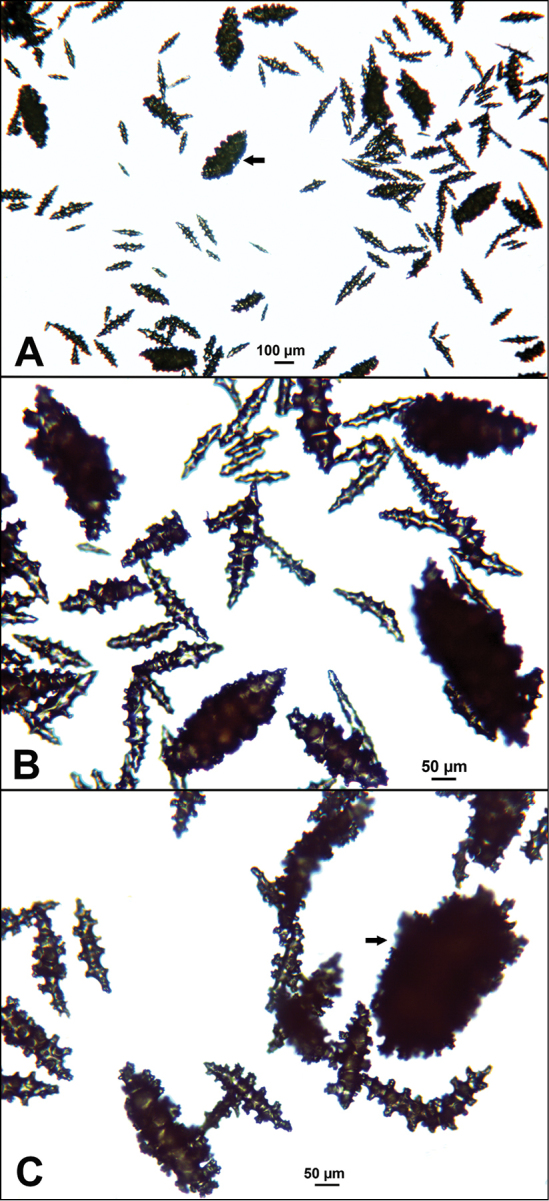
*Thesea* spp., SBMNH 265941, light microscopy array. **A** 4× magnification, showing dense sclerites as typical “football (arrow),” a key characteristic of the genus **B** Same specimen, SBMNH 265941, 10× magnification **C** One additional array, 10× magnification; shows distinctly dense warting of “typical” sclerite. Sclerites in **C** extracted from specimen T0-61, provided by research/survey staff, Los Angeles County Sanitation District.

**Figure 31.**
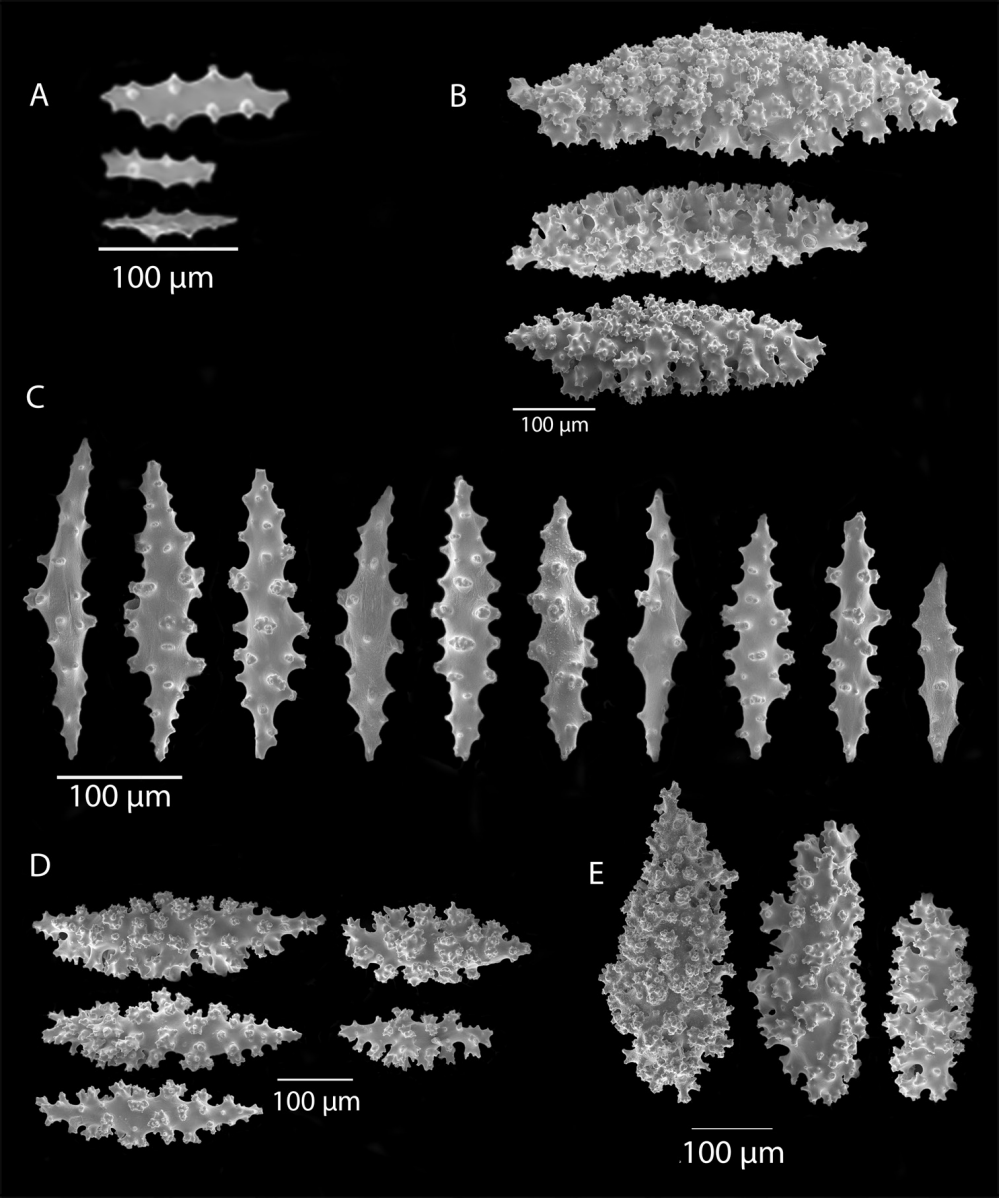
*Thesea* spp., SBMNH 422352, SEM image. **A** Possible, small developing inner coenenchymal sclerites **B, E** Characteristic, large, spheroidal sclerites of outer coenenchyme **C** Tentacular sclerites **D** Inner coenenchymal sclerites

**Figure 32.**
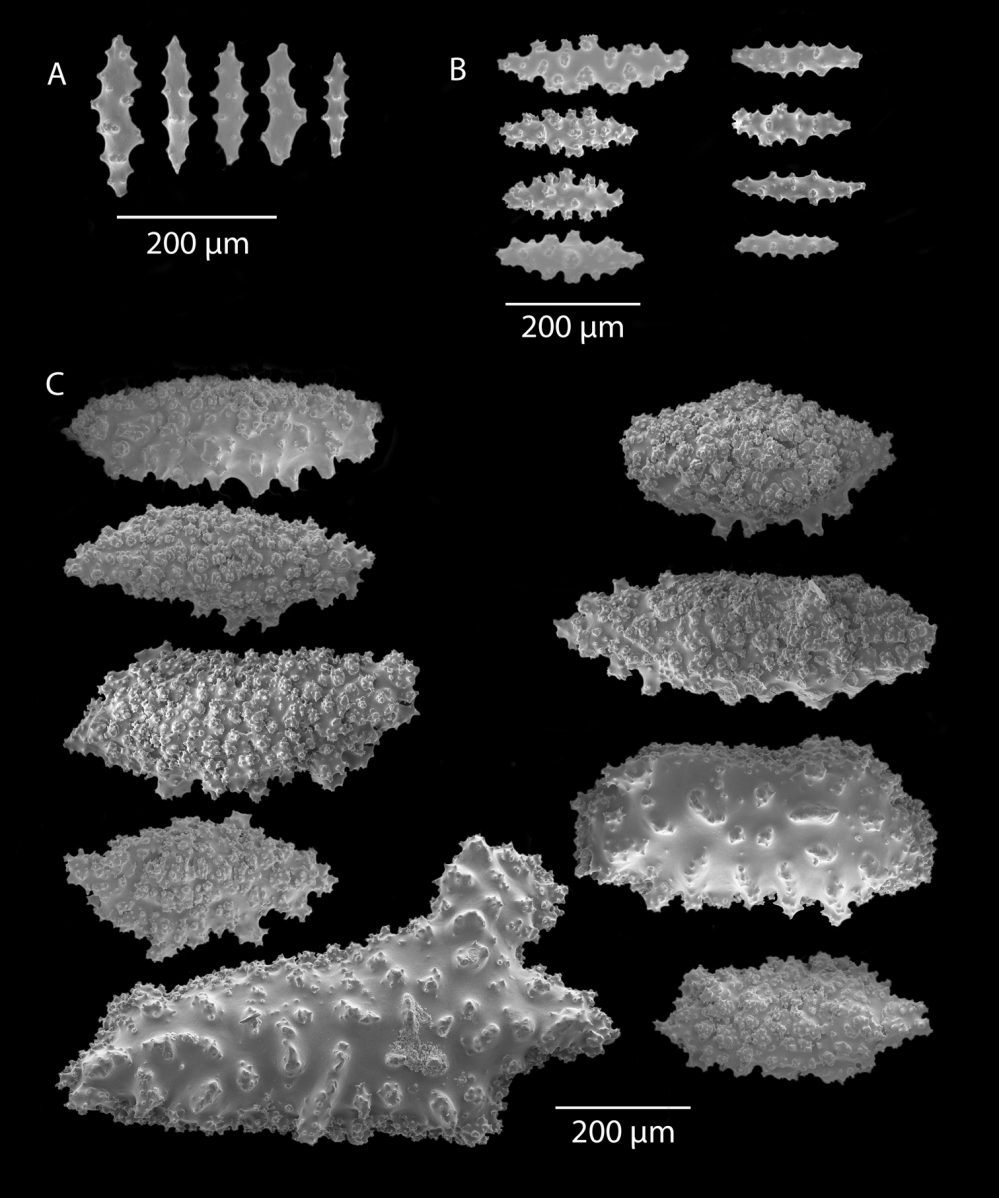
*Thesea* spp., SBMNH 422344, SEM image. **A** Possible tentacular sclerites **B** Inner coenenchymal sclerites **C** Large spheroidal sclerites of outer coenenchyme, characteristic of genus.

Figures 28A, B , 29A, B , 30A–C , 31A–E , 32A–C

? Theseafiliformis (Nutting, 1909 [non Elasmogorgia]), comb. nov. and/or. . .
? Theseavariabilis (Studer, 1894) = Psammogorgiavariabilis Studer, 1894: 67; [in Bayer 1958a: 51, 52, fig. 7].

###### Type locality and type specimens.

As a determination of species encompassed within this assortment of specimens from the eastern Pacific has not yet been established/confirmed, information regarding type locality and identification of any type specimens must await further study.

###### Material examined.

~65 lots (see Appendix 3: List of material examined).

###### Description.

*Colony* (Figure 28A) simple; long, thin, single, whip-like (wiry), unbranched stems or sparingly branched (branch can arise near small attachment disk/base, if present, or anywhere along length of long strand, at sharp angle or nearly perpendicular to primary strand; not usually longer than main strand); many specimens show neither end of branch/stem as having a base, each terminating with a bluntly-pointed arrowhead (usually three small polyps arranged in one plane) as do all branches coming off of main stem; all strands slender and flexible. Branchlets somewhat flattened, 1.0–3.0 mm wide, 1.0 mm thick; stem/branch length generally no more than 0.3 m (≤1 foot). Calyces (Figure 28B) low (~ ≤ 0.5–0.7 mm high), conical, broad domes with basal diameter ~1.8 mm (hard to determine; calyx walls slope very gradually into general surface of thin, transparent [can be, but not usually] coenenchyme); each rising slightly above general surface, nearly right angled, on all sides of stem and branches (in some colonies, appear to stand taller). Placement slightly alternate; although some calyces tend to be lateral, and alternate, actually present on all sides of stem and any branches, somewhat distant, irregularly separated by a space ~0.0–3.0 mm. Calyces ovate (sometimes round) in cross-section; longer diameter parallel with stem. Polyps usually completely retracted, almost entirely concealed by indrawn margins. Sometimes, polyps fully retracted, with large, visible opening above tentacles; margin edge easily seen, usually displaying eight triangular teeth-like projections; individual sclerites on tentacles not easily seen. Collaret not easily seen on most specimens; may not be present at all. Color of living colonies range from yellowish-beige or tannish-beige, dirty white to bright white (also perhaps bright golden yellow?); with white or cream polyps; axis pale yellow to yellowish brown to dark brown or black. Sclerites (Figures 29A, B, 30A–C, 31A–E, 32A–C) generally medium-sized spindles (average ~0.2 mm L X 0.07 mm W); largest-sized heavy, conspicuous, densely warted (dense, elongate footballs), often one-sided, covered with very jagged projections. Also many smaller sclerites: some slender spindles with surfaces covered. Largest sclerites found on stem between calyces; although often invading walls of the latter, usually of a slightly more slender type, appearing as small, short spindles arranged transversely on lower parts; a few (almost as blunt-ended scales) with several closely layered (two or three deep), vertically placed around margin, their ends forming moderately conspicuous circlet of points, annulations or oval markings around margins when viewed from above. Collaret consists of two or more circular rows of spindles; difficult to see in many colonies (see Remarks section, below). Coenenchyme filled with compact layer of short stout spindles lying lengthwise of stem; in gross examination of coenenchyme surface, stout spindles and/or spheroidal bodies very evident, dense in number. However, largest spheroidal or plate-like bodies common to genus not always abundant in sclerite arrays, but always present, exceedingly evident, very densely warted with jagged, bumpy edges.

###### Etymology.

The Latin *fili*- = “thread;” and *form*-, the Latin for “form or shape;” could refer to the slender thread- or wire-like nature of the branches; this branch pattern was seen consistently in all specimens examined (regardless of what species might be represented). Thus, specimens from at least one species group could potentially be named *Theseafiliformis*, but could not be synonymous with *Elasmogorgiafiliformis*Wright & Studer, 1889. For *T.variabilis*, variability is implied, but whether that is in regards to such characters as colony form, branch thickness or sclerite appearance, the description given by Bayer (1958) is not clear; he does, however, indicate that the colonies he examined displayed variety of color.

###### Distribution.

For this assemblage of specimens, from the northern Channel Islands, California, south to Baja, California, based on location data (see Appendix 3: List of material examined). NMNH has *T.filiformis* in their collection; those specimens were examined and they indeed belong in the genus *Thesea* (for example, USNM 30295, from San Diego, Point Loma, resembles those in SBMNH collection). It has been determined that many specimens collected by both LASD and OCSD are similar to those in SBMNH collection. NMNH has several other specimens (USNM 57172, Baja, CA; 57173 Baja, CA and 57525, from California, Point Loma) that also resembled those in SBMNH collection, or those collected by both LASD and OCSD. As to *T.variabilis*, the two specimens examined at NMNH (USNM 50633 and 50634) were both collected from La Jolla Canyon, San Diego County, CA.

###### Biology.

Found on sand/soft mud bottoms to depths of at least 30 m, based on collection data for many specimens in SBMNH collection. Branches of these colonies can be found with any number of other organisms growing on, or associated with, them. On specimens examined, both wet and dry, were found: 1) round, gall-like growths formed by a species of acorn barnacle (protruding out through the coenenchyme, 2) presence of a *Lepas*-like barnacle (found primarily on bare, exposed axis, rather than on strands with coenenchyme intact; one specimen with a note: “*Scalpellum*,” where barnacle was conspicuously affixed to strand), 3) presence of ovulid snails (genus *Neosimnia*), 4) intertwined with arms of Ophiuroidea, 5) sometimes with other kinds of cnidarian attached at base of colony; often mixed in with species of sea pen, having the same body form (thin strand-like colonies) and 6) some with what appeared to be a kind of worm (? ribbon worm) curled around the branch strands.

Muzik (1979) made the comment that “colony shape is largely environmentally, not genetically determined” in the *Thesea-Filigella-Muricella* genera cluster. Colonies will be “large, planar, even branched . . . when on a large firm substrate, but filiform on rubble.” The species group discussed here consistently grew on something other than large, firm substrates (based on consistent slender, filiform appearance of branch strands), confirmed through images provided by staff of LACSD and OCSD. Their growth and presence on a softer or rubble bottom would dictate aspects of their biology, such as materials fed upon, means of anchorage (or lack thereof), etc.

###### Remarks.

Of interest are specimens belonging to the genus *Thesea* that are consistently, and regularly collected by Los Angeles and Orange County Sanitation District (LACSD and OCSD) staff; these look very comparable to many lots of specimens housed in SBMNH collection. Both LACSD and OCSD regularly label the majority of their collected samples as “*Thesea* species B;” it appeared that this is the species described here, so plentiful in the SBMNH collection. These will have a tan, light beige to dull cream coloring. Based on the number of lots housed in SBMNH collection (see Appendix 3: List of material examined), and the consistent collection records that both LACSD and OCSD report, *Thesea* is very common in California waters. Also of interest are specimens (determined as belonging to this genus), present in both SBMNH collection (in fewer numbers) and collected by LACSD and OCSD, that have a noticeably yellower color than the typical *Thesea* described above. It was the range of color displayed in the SBMNH collection lots (and of those collected regularly by LACSD and OCSD) that was of more than passing interest. Nutting (1909) stated color for *Elasmogorgiafiliformis* from California (incorrect genus) as clear, light gray, with axis dark brown, and Nutting (1912) stated color (for same species, again incorrect genus) as rather dark brown; axis almost black (was unable to determine if these two statements by Nutting, regarding color, were for dry specimens, but likely). Multiple wet specimens examined revealed variable color in both coenenchyme and axis, without consistent color pairing, despite no real differences in appearance of sclerites (aside from color). With no apparent, clear-cut differentiation in variation of the sclerites (aside from color) in most of the arrays examined from colonies colored beige, white, or yellow, initial consideration was that all were several color morphs of the same species. At present, colonies are being treated as such; more extensive examinations currently being conducted may reveal differences that would warrant colonies of varying color being given separate taxonomic designations. Additionally, a small number of lots within the collection have far smaller, stouter (almost equal in width and length) dense sclerites; these are very similar in length to the less heavily warted spindles that are seen. The possibility that these specimens need to be separated out has not been excluded, but different environmental circumstances may account for these variations.

Determination as to whether or not an actual operculum was present on calyces was (and is as yet) not clarified. Kinoshita (1909), Kükenthal (1924) and Deichmann (1936) all specifically made mention, and described arrangements of, sclerites that form the operculum in this genus. In one known species the operculum was described as being strong, each flap consisting of three spindles forming an acute-angled triangle, reinforced by others lying parallel to these, or disposed longitudinally on distal parts of tentacles. In another species, the operculum was described as being composed of three to six pairs of spindles in converging double rows. Neither pattern was clearly seen in specimens examined. Considering the accepted definition of an operculum, and those families where one is quite evident, it did not seem that in this genus there is such a structure in the correct sense of that term; it might be more accurate to speak of a collaret, or a crown and points arrangement. Neither Kinoshita (1909), Kükenthal (1924) or Deichmann (1936) provided any clear illustrations of the situations they discussed regarding an operculum. Fabricius and Alderslade (2001) speak of the family as having crown and points, with no mention of an operculum. Despite an issue of semantics, or incorrect use of the term operculum, the simple fact was that very few colonies examined provided even one or a few calyces where a clear view could be had of what was covering over the tentacles. The eight tooth-like projections of the calyx margin were evident (on many), but any pattern of sclerites that might have been overlying the indrawn tentacles was another matter. The dry, often brittle nature of many of the specimens compounded the problem. With fresh material, more malleable to work with, this question could definitively be answered.

Regarding the species *T.* [non *Elasmogorgia*] *filiformis* (Nutting, 1909), Nutting reported a specimen taken from off San Pedro, California coast, in the University of California collection, as well as one described in Nutting (1909), from ‘Albatross’ station 4349, Point Loma light-house, NE 6.5 miles, 136–244 m (unable to locate them, thus these specimens could not be examined). He also reported specimens taken in the Dutch East Indies at 112 meters, and the specimen described in Nutting (1912) was taken from ‘Albatross’ station 4837, Tateisha Zaki Light, S 53 E 8 miles, 104 m. These latter two may not be the same species as the other two mentioned.

Regarding *Theseavariabilis*, there are no apparent specimens of the species in the SBMNH collection (see Appendix 3: List of material examined). Based on collection data for the specimens at NMNH (USNM 50633 & USNM 50634, from La Jolla Canyon, San Diego), this species required inclusion here but was unable to determine if these specimens are this species or actually a morph of *T.* [non *Elasmogorgia*) *filiformis*. Commonly called the White gorgonian, “*Theseavariabilis* resembles very narrowly by its exterior aspect some of the *Thesea’s* of the ‘American Indies’ ” (Bayer 1958). Drawings of sclerites from *Theseaguadalupensis* that Bayer (1958) provided for comparison with *T.variabilis* showed that the resemblance is more than superficial. The only real difference seen was that the sclerites of *T.variabilis* were smaller than those of *T.guadalupensis* (and of roughly the same size as those seen in *T.* [non *E.*] *filiformis*). “The sclerites are in limited accord, as are the external characters. Thus, there is no doubt that the eastern Pacific species belongs to the genus *Thesea*, a genus that was formerly thought to be restricted to the ‘American Indies.’ ” While noted on a list found in SBMNH files, indicating California sites and depth ranges (40–46 m, in the La Jolla area, only), there is no certainty that other coastal areas of California (even just southern California), would be an actual locality for this species. In Bayer’s personal SEM files, images were found for *Theseavariabilis* Studer = *Psammogorgiavariabilis*. These did not fully resolve identification issues but does lead to further consideration of this species belonging to the genus *Psammogorgia* rather than the genus *Thesea*. Found at depths greater than 100 ft [~33 m], on the two specimens Bayer examined (1958), barnacles that were present formed prominent cysts on the branches.

Overall branching pattern described puts *T.variabilis* at odds with the colony form commonly seen and named as *Thesea* spp. in southern California, where branches simply appear as long thin strands, often with no apparent base, both ends of each strand/branch frequently terminating in a flattened, arrowhead shape. From images sent by LACSD and OCSD, many have an attachment to the substrate simply by being partially buried in the soft bottom sand or mud. USNM 50633 and USNM 50634 appeared more as a flattened bush, with indication of an actual base structure, and had branches of a thicker diameter, with a slightly more yellow coloration. They did not appear as the many colonies in the SBMNH collection, but their sclerites were very comparable. Thus, there might be the possibility that *T.variabilis* and *T.* [non *E.*] *filiformis* are the same, with variable colony form (attached forms, with actual base structure, more extensively branched, even almost as a fan, while those with no attachment base more thread-like); perhaps this is a case of different living conditions dictated by the surrounding environment resulting in different colony morphologies.

Generally, this multiple-lot assemblage is composed of specimens that are best described as a conglomerate of what could be called *Theseafiliformis* (with few possible morphs) or is composed of a few different species. Based on location data for all, *T.filiformis* for all may be the better choice; further studies will need to be done, but there is no doubt that the genus *Thesea* is commonly encountered in southern California waters.

### Diagnosis of the Suborder Calcaxonia Grasshoff, 1999

Group of families lacking chambered axial core. In axis, large amounts of non-scleritic calcareous material present, either in the form of calcite or aragonite, deposited between horny fibers, or present as central core, or with solid internodal sections alternating with nodes of pure gorgonin in segmented axis.

**Remarks.** With the exception of one species (*Plumarellalongispina* Kinoshita, 1908a), Calcaxonia is not well represented in the SBMNH collection, although there is every indication that calcaxonian species are represented in the California Bight through multiple genera and species (see Appendix 3: List of material examined). Several species (*Callogorgiakinoshitai* (Kükenthal, 1913) and *Parastenellapacifica* Cairns, 2007) are each represented in the collection by no more than three to six separate colonies (plus numerous colony fragments); species that fall within the genera *Isidella* Gray, 1858 and *Keratoisis* Wright, 1869 are present in the collection, but are represented by only one or two colonies each. As all are deep-water taxa, this is not surprising. Unfortunately, specimens provided little material to work with, such that comparing/contrasting one specimen with another was not always possible. Furthermore, a number of the specimens in the collection are not in good shape; some are represented by a single branch, or portion thereof, rather than an intact, complete colony. In some, if all colony branches are present, the coenenchyme is largely or completely lacking. Even having material from another institution did not always help; often these are only identified to genus (due to very recent collection events), and when they are identified to species (often as new species), the material in the SBMNH collection often lacked some key structure that could have made comparison with a well-known, or newly described species, possible. Thus, the descriptions given here for species in this suborder from the collection are not always complete. More material is required, and further extensive comparisons with other specimens from other institutions are needed, to clarify not only what is present in the SBMNH collection, but to also clearly indicate which species have regular occurrence in the California Bight.

#### Key to Families represented in SBMNH collection (Suborder Calcaxonia)

**Table:** 

1	Axis jointed (segmented), with articulation of alternating, purely horny (gorgonin) nodes and nonscleritic calcareous internodes; calcareous material appearing radially oriented; internodes solid or hollow, but with no soft, central chord; polyps either retractile or non-retractile	**Family Isididae**
–	Axis in cross section not jointed, but continuous, with strong calcification in form of undulating concentric layers of strongly calcified material embedded in gorgonin, resulting from a longitudinal (not radial) pattern of calcification; core not a soft, hollow-chambered central one; polyps always non-retractile; sclerites usually scales	**Family Primnoidae**

#### List of species of Calcaxonia Grasshoff, 1999

Class Anthozoa

Subclass Octocorallia Haeckel, 1866

Order Alcyonacea Lamouroux, 1816

Suborder Calcaxonia Grasshoff, 1999

Family Primnoidae Milne Edwards, 1857

*Callogorgiakinoshitai* (Kükenthal, 1913)

*Parastenellapacifica* Cairns, 2007

*Parastenellaramosa* (Studer, 1894)

*Plumarellalongispina* Kinoshita, 1908

*Primnoapacifica* Kinoshita, 1907

*Narella* Gray, 1870

Family Isididae Lamouroux, 1812

*Acanella* Gray in Wright, 1869

*Isidella* Gray, 1857

*Keratoisis* Wright, 1869

*Lepidisis* Verrill, 1883

#### Descriptions of species of Calcaxonia Grasshoff, 1999

##### Family Primnoidae

Taxon classificationAnimaliaAlcyonaceaPrimnoidae

Milne Edwards, 1857

###### Diagnosis.

Axis of strongly calcified material embedded in gorgonin, unjointed, arranged in undulated concentric layers; core not a soft, chambered central chord. Attachment base a calcareous disc; rarely, a branched, rhizoidal structure. Colonies usually profusely branched, rarely flagelliform. Polyps single, in pairs, or in regular whorls, heavily armored with calcareous scales (sclerites primarily scales in all species), permanently exsert; in contraction, tentacles in-folded. Polyps protected by eight triangular scales making up distinct operculum, below which scales of polyp body aligned in eight rows, some of which may be reduced or missing on adaxial side; rarely (single species) scales not regularly arranged, operculum undifferentiated. In coenenchyme, a layer of plates or scales, commonly elongate, some with inner layer of stellate sclerites. Scales always distinguished by cruciform extinction pattern seen in polarized light.

###### Remarks.

A rationale for the distinction between the use of the words calyx and polyp required in reference to the family. S Cairns (pers. comm.), in a conversation with P Alderslade some years ago, determined that the term calyx should be reserved for those polyps that can contract to a small mound (such as those seen in the plexaurids), and that the primnoid morphology is a polyp. Thus, there is no calyx to be seen in this family; projections and living animals are called polyps; that usage has been incorporated here.

##### Genus Callogorgia

Taxon classificationAnimaliaAlcyonaceaPrimnoidae

Gray, 1858

Gorgonia Pallas, 1766: 160 (pars). Linnaeus 1767: 1289 (pars). Ellis and Solander 1786: 67 (pars).
Muricea Dana, 1846: 675 (pars).
Prymnoa Ehrenberg, 1834: 357 (pars).
Primnoa Milne Edwards & Haime, 1857: 139 (pars). von Koch 1878: 457; 1887: 85.
Callogorgia Gray, 1857 [1858]: 286. Bayer 1956: F220; 1961 [1962]: 296. Carpine and Grasshoff 1975: 102. Bayer 1981: 938; 1982: 119, 120. Bayer and Stefani 1989: 455. Bayer 1998: 162, 163. Cairns and Bayer 2002: 841–845; 2009: 29, 40. Cairns 2010: 425 (Hawaiian species); 2016: 58 (New Zealand species); 2018a: 6 (key to Indo-Pacific species); 2018b: 3. Cairns and Wirshing 2018: 8, 18, fig. 40.
Calligorgia Gray, 1870: 35 (unjustified emendation). Studer 1878 [1879]: 645; 1887: 51.
Fanellia Gray, 1870: 45. Bayer 1982: 134, 135. Bayer and Stefani 1989: 470, 471. Cairns and Bayer 2009: 40, 41. Cairns and Wirshing 2018: 8, 18.
Xiphocella Gray, 1870: 56 (type species, Gorgoniaverticillata: sensu Esper, 1797: 156, by monotypy).
? Callicella Gray, 1870: 37 (type species, Callicellaelegans Gray, 1870, by monotypy).
Caligorgia Wright & Studer, 1889: 75–77 (pars; unjustified emendation). Versluys 1906: 55 (pars). Kükenthal and Gorzawsky 1908: 19. Kinoshita 1908a: 34.
Nutting, 1908: 574. Kükenthal 1912: 320(?); 1915b: 146; 1919: 362 (pars); 1924: 267. Deichmann 1936: 158.

###### Type species.

*Gorgoniaverticillata* Pallas, 1766 (by monotypy).

###### Diagnosis.

Colonies usually branched pinnately, some rarely dichotomously, mostly in one plane; axis longitudinally striated, commonly iridescent. Polyps in regular whorls, strongly bent inward toward axis. Adaxial rows of body scales reduced; opercular scales distinctly differentiated from body scales, not overreached by marginals (which do not bend inward over them); sclerites usually elaborately sculptured externally, with ridges, crests or small granules; cortical sclerites thick, pebble-like or more elongate.

###### Remarks.

WoRMS Database (Cordeiro et al. 2019) gives this genus accepted status, with this spelling.

##### Callogorgia kinoshitai

Taxon classificationAnimaliaAlcyonaceaPrimnoidae

(Kükenthal, 1913)

**Figure 33.**
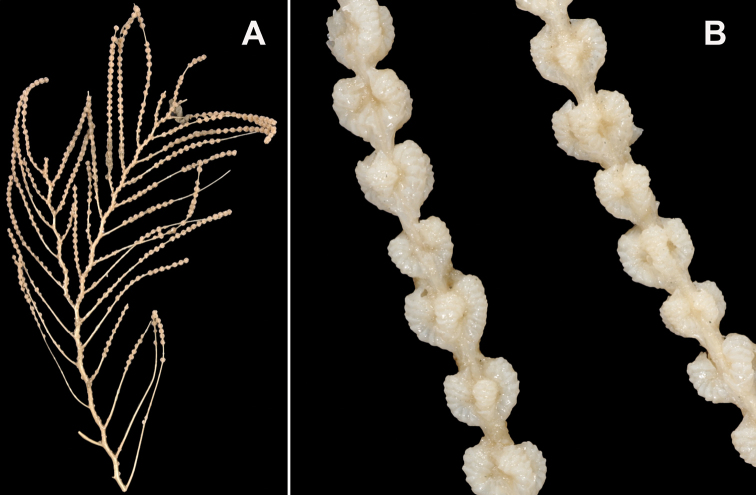
*Callogorgiakinoshitai*, SBMNH 422990. **A** Colony; height (base missing) ~30 cm **B**SBMNH 422982, branch close-up, illustrating arrangement of polyps in whorls, each polyp curving strongly upward and inward toward branch.

**Figure 34.**
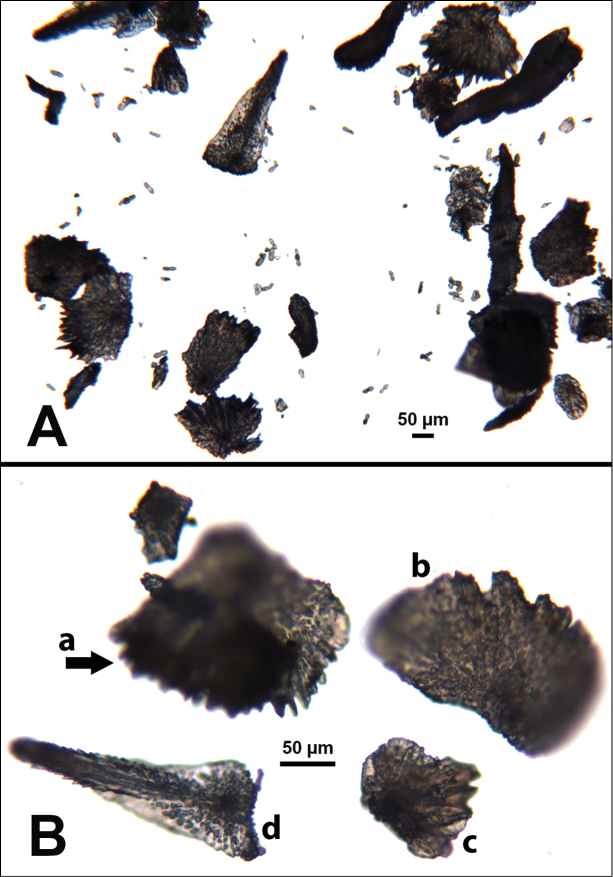
*Callogorgiakinoshitai*, SBMNH 422982. **A** Light microscopy array, 4× magnification, showing variety of large scales (body wall, coenenchymal, opercular, etc.) as well as very small coenenchymal rod-like sclerites **B** Array of representative scales, 10× magnification, SBMNH 422982; **a** abaxial body wall scale, indicated by arrow **b** possible inner lateral or abaxial from polyp base **c** outer lateral scale **d** opercular scale. Opercular scales average 239 µm in length, body wall scales range in breadth from 117–217 µm and very small spindles ~28 µm in length.

**Figure 35.**
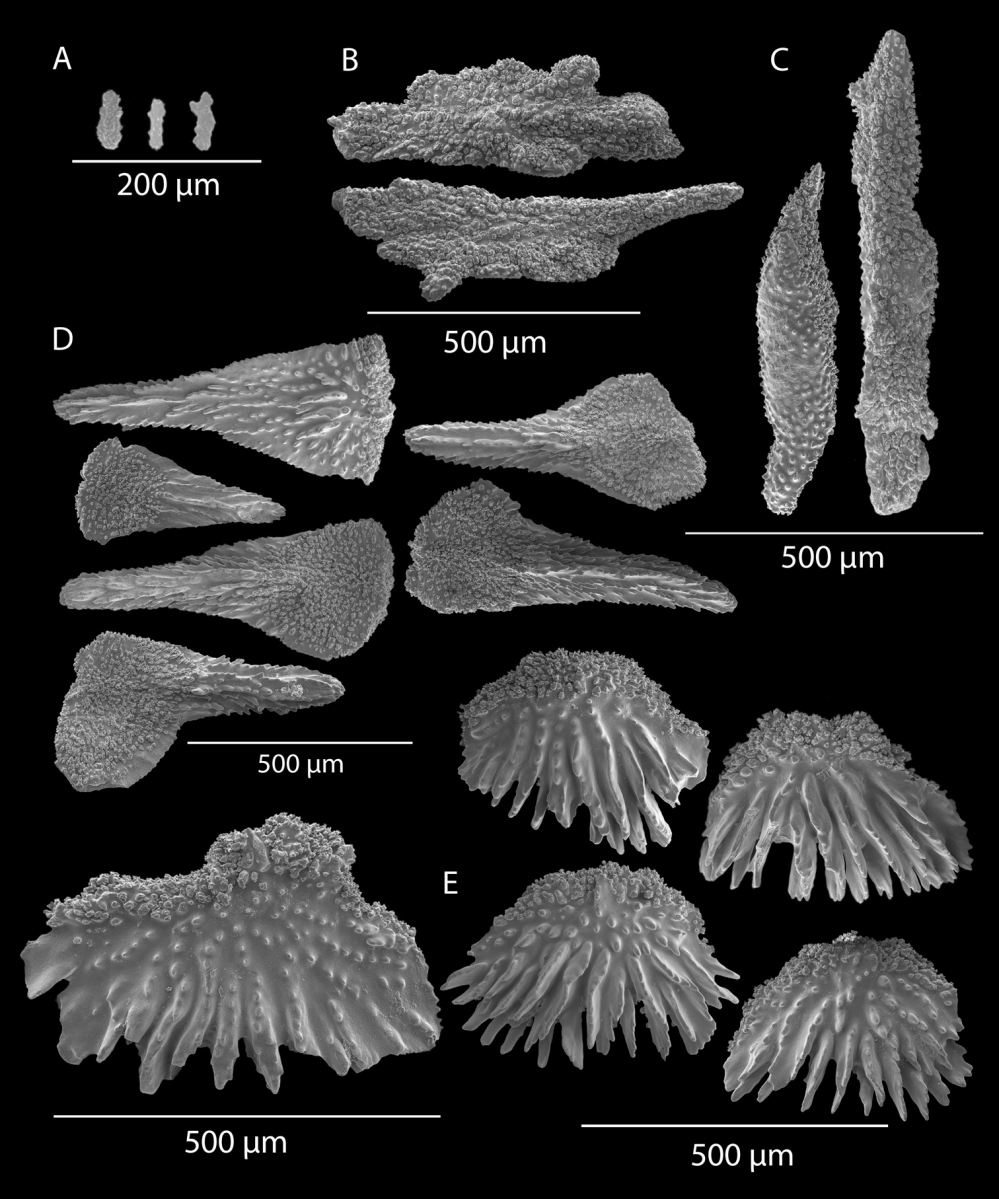
*Callogorgiakinoshitai*, SBMNH 422982, SEM image. **A** Small, developing coenenchymal rods **B** Very flattened, “odd” coenenchymal rods **C** Coenenchymal rods **D** Opercular scales **E** Highly ridged abaxials (polyp tip). Sclerite forms shown here correspond to figures shown in Cairn and Bayer 2002 for species in the genus.

Figures 33A, B , 34A, B , 35A–E

Callogorgia kinoshitae Kükenthal, 1913: 264–266; text figs E, F, pl 8, fig. 10 (= Caligorgiakinoshitae Kükenthal, 1913: 264–266 [spelling difference]); 1919: 370; 1924: 270.
Callogorgia kinoshitae : Bayer 1982: 122. Cairns 2007b: 512 (listed). Cairns and Bayer 2009: 29 (listed).
(?) Caligorgiasertosa Wright & Studer, 1889: 75–77. Nutting 1909: 715.

###### Type locality.

USA, California, 218–2472 m. Possible collection location for type La Jolla, San Diego, based on work of Kükenthal (1913; 1924).

###### Type specimens.

Repository of type(s) unknown.

###### Material examined.

6 lots (see Appendix 3: List of material examined).

###### Description.

*Colony* (Figure 33A) flabellate, usually branched in regularly alternate, pinnate pattern; some colonies (often main branches) rarely dichotomous; most branches in one plane. Maximum colony height over 30 cm (base excluded); average height of colonies in SBMNH collection ±15 cm. Central stem slightly bent in geniculate (jointed, zig-zag) pattern, giving off branches at angles or joints; few branches give off branchlets in similar manner. Distance between branches/branchlets on same side of central stem roughly one cm (slightly larger than one cm closer to base and less than one cm toward tips of branchlets). All branchlets unbranched, parallel to each other. Distal ends of branchlets extremely thin, more flexible, with branchlets often recurved back on themselves. Axis stiff, longitudinally striated; creamy yellow to tan, covered with fairly thin coenenchyme. Color of living colony (?)white to creamy-white; perhaps very light pinkish-beige; color in alcohol creamy whitish-beige to light tan. Five or six polyps (rarely four) regularly arranged in each whorl (most common number being five); whorl diameter 2.1–2.2 mm; generally, four to five whorls per centimeter of branch length. Minimal distance between whorls no more than 1.0 mm (often less), but evident. Polyps 2.0 mm tall; slightly clavate, covered in four to eight rows of nearly spindle-shaped (rods) sclerites; polyps strongly curved from base outward, upward and inward toward axis (Figure 33B), thus apertures directed toward stem or branch. Sclerites (Figures 34A, B, 35A–E) predominantly scales, flattened (sometimes oblong, fusiform; some appearing as flattened caveman clubs) on stem and branches, with long tooth-like spines, and radiating ribs. Outer sclerite surface may also have many small to medium-sized warts, bumps and granules. Scales imbricating (like roof tiles), fan-shaped on polyp walls. Aperture edge of polyp with ctenate marginal scales, inside of which are bases of eight opercular scales; these form tall, pyramidal opercula, with height ~0.5 mm. Opercular scales (Figures 34Bd, 35D) distinctly differentiated from body scales, not overreached by marginals, not bending inward over them. Individual opercular scales elongate triangles, especially on abaxial side, forming a conspicuous, elongated spine when polyp is fully retracted; these scales bear thickened, longitudinal ridges on their inner surface, ending with truncated points. Opercular scales up to ~0.65 mm long by 0.2 mm wide at broader end; adaxial opercular scales much smaller. Upper layer (ring) of marginal scales (Figure 35E) large, with radiating ribs, furrowed at their edge; others (proceeding proximally) show these markings feebly, if at all. Longitudinal rows of scales on polyp body commonly numbering seven (rarely eight), best seen on abaxial and lateral sides (inner lateral scales number four on each side of polyp); only abaxial rows of body scales complete. Adaxial rows reduced or absent; if present, generally two scales placed distally, two proximally, revealing large area of naked adaxial wall; thus, total number of scales within a row varies, but typically eight (with six to nine possible) scales in row; most numerous on exposed, abaxial side. Largest body wall scales, abaxial (Figures 34Ba, b, 35E); abaxial scales near tip of polyp smaller, those of adaxial side up to ~0.1 mm across by 0.1 mm tall. Lateral scales slightly smaller (Figure 34Bc). Coenenchymal sclerites (Figure 35A–C) dense, as elongated, nearly spindle-shaped rods often covered with numerous thorns or prickles.

###### Etymology.

Named in honor of Kumao Kinoshita of Japan (Cairns 2018).

###### Distribution.

*Callogorgiakinoshitai* appears to extend from Monterey Bay to as far south as Chile, based on collection location data found recorded at several institutions (see Appendix 3: List of material examined). Based on specimens housed in SBMNH collection, it would appear that the species does extend further north, into waters off Oregon and Washington (USA).

###### Biology.

Generally found in deep water (averaging 800–1,000 meters). Intertwined amongst branches may be found moderate to large Ophiuroidea, along with either what appear to be anemones (quite large, very fleshy and wrinkled) and/or possibly a type of acorn barnacle, attached to stems and branches.

###### Remarks.

Kükenthal (1919, 1924) speculated that the species *C.kinoshitae(i)* might be a junior synonym of *Caligorgiasertosa* Wright & Studer, 1889 (note error in spelling of genus name), as described by Nutting (1909). Nutting (1909) indicated five localities for what he called *C.sertosa*, all in the vicinity of USA, California, San Diego, Point Loma light-house. Nutting also established the type for *C.sertosa*, that being collected at Station 192, off Kei Island, South Pacific, 255 m, by R/V ‘Challenger’. Perhaps Nutting’s specimens from the San Diego area should be ascribed to this species rather than to *C.sertosa*. In any event, the two are indeed separate species. Researchers with greater exposure to, and expertise on, this species (SD Cairns) should be consulted regarding legitimacy of *C.sertosa* as senior synonym. Cordeiro et al. (2019) does not show this synonymy in the WoRMS Database. Earlier descriptions for both *C.kinoshitae(i)* and *C.sertosa* found in Kükenthal (1919) clearly indicated the distinct differences used to distinguish between these two species. Based on locations of collection events, with *C.sertosa* having its type collected from Kei Island in the South Pacific, the two appear to be separate, distinct species.

##### Genus Parastenella

Taxon classificationAnimaliaAlcyonaceaPrimnoidae

Versluys, 1906

non Stenella Gray, 1866: 213 [a cetacean].
Stenella Gray, 1870: 48. Studer 1878 [1879]: 643; 1887: 50. Wright and Studer 1889: 56 [pars; S.doederleini, S.spinosa]. Kinoshita 1908a: 27, 28. Kükenthal 1915b: 151, 152 [pars]; 1919: 443–445 [pars; key to species]; 1924: 303 [pars; key to species]. Molander 1929: [pars]. Aurivillius, 1931: 289, 290 [pars].
Stenella (Parastenella) Versluys, 1906: 39, 45.
Candidella (Parastenella) Bayer, 1956: F222.
Parastenella Bayer, 1961: 295 [ill. key to genus]; 1981: 936 [key to genus]. Bayer and Stefani 1988: 454 [key to genus]. Cairns 2007a: 245–247; 2007b: 518. Table 2 [generic revision, tabular key to species]. Cairns and Bayer 2009: 31, 45, 46. Cairns 2010: 434 [key to species]; 2011: 23; 2016: 94–96

###### Type species.

*Stenelladoederleini* Wright & Studer, 1889; subsequent designation Bayer 1956a.

###### Diagnosis.

Colonies primarily branched, planar dichotomous; occasionally slightly bushy. Polyps arranged in either whorls of up to four, in pairs, or isolated, generally standing perpendicular to branch. Operculum well developed, opercular scales decidedly keeled on inner surface. Marginal scales eight, in alternate position with respect to opercular scales. All polyps, generally, completely covered with five to eight longitudinal rows of body wall scales; outer surfaces covered with small granules. Coenenchymal scales arranged in one layer. Tentacular rods sometimes present.

###### Remarks.

Genus holds accepted status, shown in WoRMS Database (Cordeiro et al. 2019).

##### Parastenella pacifica

Taxon classificationAnimaliaAlcyonaceaPrimnoidae

Cairns, 2007

**Figure 36.**
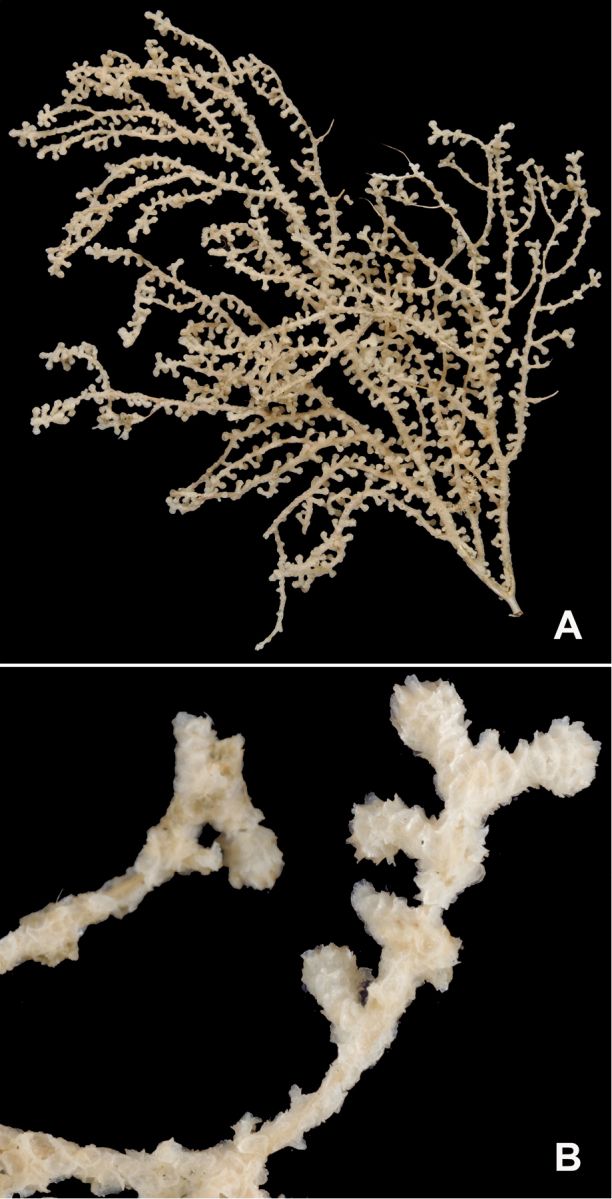
*Parastenellapacifica*, SBMNH 422983. **A** Colony (attachment base missing), ~15 cm × 13 cm, at widest point **B** Branch tip close-up.

**Figure 37.**
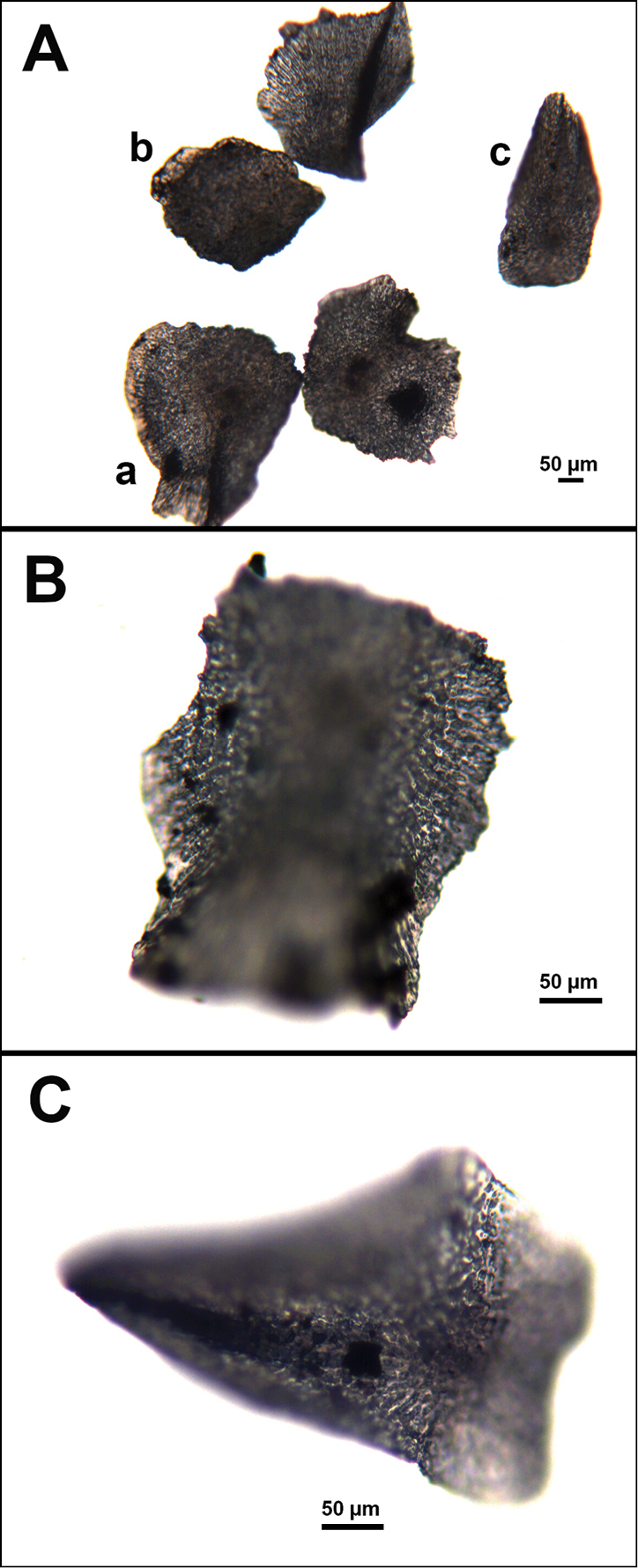
*Parastenellapacifica*, SBMNH 422983. Light microscopy arrays, scleritic scales. **A** An assortment **a** possible marginal scale **b** abaxial body wall scale **c** opercular scale **B** 10×-magnified image; marginal scale **C** 10×-magnified image; opercular scale.

**Figure 38.**
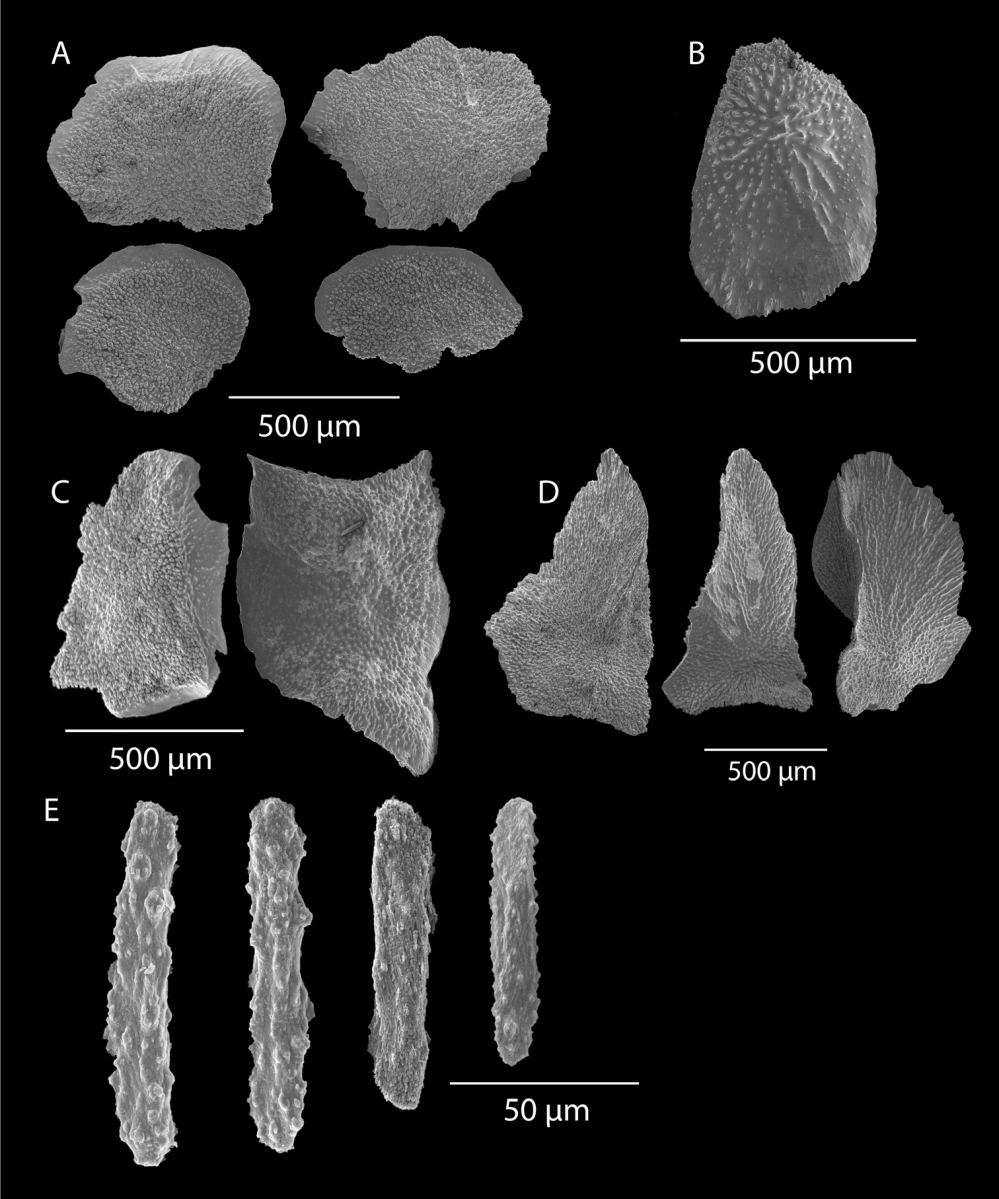
*Parastenellapacifica*, SBMNH 422983, SEM image. **A** Abaxial body wall scales **B** Coenenchymal scale **C** Marginal scales **D** Opercular scales **E** Pinnular sclerites. Compare to those shown in Cairns 2007 (Figure 9).

Figures 36A, B , 37A–C , 38A–E

Parastenella pacifica Cairns, 2007b: 526, 527; figs 1C; 8, 9.

###### Type locality.

USA, Oregon, west of Cape Meares, 45°25'18"N, 125°11'01"W, 1498–1527 m.

###### Type specimen.

**Holotype**USNM 1071799 [dry]; type was not examined.

###### Material examined.

1 lot (see Appendix 3: List of material examined).

###### Description.

*Colony* (Figure 36A) with dichotomous branching, somewhat irregular, generally in one plane; some SBMNH specimens slightly bushy, flabellate, up to +30 cm in height (next largest, 14 cm tall). In largest colonies branchlet tips tend to droop down, curling slightly back on themselves (Figure 36A); branching intervals vary from ~3.5 cm distance at lower end of main stem (near base) to less than 1.0 cm near branch/branchlet tips. Polyps (Figure 36B) with opercula well differentiated; usually spaced 0.5–1.5 mm apart in/as singles, pairs or whorls of up to three, erect (perpendicular to axis), or slightly bent downward toward stem. Polyp height 2.0–3.5 cm, flared distally with slender, delicate stalk, heavily armored with calcareous scales. Polyps found on numerous branches, tending to favor one side of colony. Axis as described for family; visible through single layer of white, translucent coenenchymal scales; dark to light brown in color. Color of living colony (?)cream or white; in alcohol, cream to light tan. Sclerites are scales (Figures 37A–C, 38A–E); marginal scales (standard number eight) alternating in position from opercular scales (latter forming distinct operculum, creating obvious projection out from polyp). Marginal scales (Figures 37Aa, 37B, 38C) all of similar shape and size, most showing broad, shallow apical flute; these generate symmetrical rosette when viewed from above. Submarginal body wall scales (Figures 37Ab, 38A) roughly arranged in eight longitudinal rows, each row with three to four scales that appear to overlap those in adjacent rows; distal end obviously rounded, no fluting apparent; flutes absent on submarginal abaxial body wall scales. Otherwise, polyp completely covered with body wall scales, including adaxial region. Opercular scales (Figures 37Ac, 37C, 38D) alternate with marginal scales (as opposed to overlapping them) around polyp; triangular shape, prominently keeled on inner surface; most all of similar size (0.5 mm in length, on average). Coenenchymal scales (Figure 38B, possibly) generally elliptical, very evident on branches (resembling sea pansy rachis or water lily pad), in one thin layer; few with irregular shape. Pinnular sclerites (Figure 38E) small rods, with granular surface.

###### Etymology.

The species name *pacifica*- in reference to its general location; stated to be closely similar to *Parastenellaatlantica* (Cairns (2007b). Cairns suggested that these could form a geminate (twin species) pair, differing largely in having ranges in different oceans. The species designation is listed as accepted in WoRMS Database (Cordeiro et al. 2019)

###### Distribution.

Deep-water species (~1,500–2,086 m, currently known to live on the continental slope off Oregon up to British Columbia (Queen Charlotte Islands); see Appendix 3: List of material examined. Material in the SBMNH collection came from an area north of the California Bight’s northern limit; whether it will be found further south (into the California Bight) remains to be seen.

###### Biology.

Of the many fragments/partial colonies present in the one lot from the SBMNH collection, one of the fragments has bits of a distinguishable, pale orange ophiuroid (brittle/basket star) intertwined/tangled within it. This could either be an artifact of collection or a true living condition. The specimen from Moss Landing Marine Labs (see Appendix 3: List of material examined) also showed presence of Ophiuroidea; based on the nature of their location, etc. within the colony, likely a living situation, not an artifact.

###### Remarks.

Sclerites in specimens from SBMNH were consistently a bit smaller than those from holotype shown in Cairns (2007b). Furthermore, the SBMNH material not generally in good condition; was often difficult to get good microscopic arrays showing enough of the different forms of body wall scales (abaxials, laterals and adaxials) so as to see clear differences. The coenenchymal sclerites on branches were very evident, however, and examination of sclerites showed clearly the broad, shallow fluting. While tentacular rods are considered common in this species, sometimes very few would be found in the fragments examined; the condition of many of the polyps may partly explain their absence. Further examination of undamaged colonies, collected from the same area, may better reveal their presence.

##### Parastenella ramosa

Taxon classificationAnimaliaAlcyonaceaPrimnoidae

(Studer, 1894)

Stenella ramosa Studer, 1894: 64, 65.
Stenella (Parastenella) ramosa (Wright & Studer, 1889): 56. Versluys 1906: 47, 48. Kükenthal & Gorzawsky, 1908: 34, 35. Kükenthal 1919: 445; 1924: 303.
Parastenella ramosa Cairns, 2007b: 518–523, figs 1E, 4, 5. Cairns and Bayer 2009: 31 (listed). Cairns 2011: 24, 25.
? Stenelladoederleini Studer, 1894: 64; see Remarks, below.

###### Material examined.

No specimens in SBMNH collection (see Appendix 3: List of material examined).

###### Remarks.

Species included here as collection records examined (see Appendix 3: List of material examined) show a distributional range that includes the California Bight. Based on those collection records, this is a deep-water species that has been collected off the west coast of Central America, and from areas of the California coast, to just north of the Santa Barbara Channel Islands (Rodriquez Seamount). It has also been collected from Monterey Bay (Davidson Seamount), north to Oregon, Washington, Vancouver Island to Gulf of Alaska; 665–1750 m. Cairns (2011) stated that the known distributional range of this species now extends west to Adak Canyon in the Aleutian Islands and the Commander Islands, Russia. This represents a substantial range, encompassing the California Bight region.

Cairns (2007b) stated that given the similarity of *Parastenelladoederleini* (Wright & Studer, 1889) and *Parastenellaramosa*, “it is likely that Studer’s (1894) identification of *S.doederleini* from off Panama at 1,429 m (specimen missing from MCZ), taken quite close to the type locality of *P.ramosa*, is probably also *P.ramosa*.” This species is generally most similar to the type for the genus, *P.doederleini* (Wright & Studer, 1889). That specimen was collected from off Sagami Bay, Japan at 3,427 m. Cairns (2007b) differentiated between the two; *P.doederleini* has more elongated and slender marginal flutes, more delicate polyps and coenenchymal scales with one or more small rounded knobs either at their center and/or on their perimeter. Specimens of *P.ramosa* examined (indicated in the Appendix 3: List of material examined), exhibited polyps more distinctly directed downwards and the marginal flutes were slightly broader than those seen in *P.doederleini*. A check of the WoRMS Database (Cordeiro et al. 2019) show both *P.ramosa* and *P.doederleini* as separate, accepted species. As well, *P.ramosa* can easily be distinguished from *P.pacifica* by the latter having obviously broad, shallow marginal flutes, eight rows of submarginal body wall scales and the absence of flutes on submarginal abaxial body wall scales; *P.ramosa* exhibits narrow, tubular marginal flutes, five rows of submarginal body wall scales and abaxial body wall scales with flutes. Additionally, Cairns (2011) stated that the confirmed presence of nematocyst pads on the inner surface of the marginal scales in this species might be the case for all species in the genus, as suggested in Cairns, 2010.

##### Genus Plumarella

Taxon classificationAnimaliaAlcyonaceaPrimnoidae

Gray, 1870

Cricogorgia Milne Edwards, 1857: 6, pl B2, fig. 6 [nomen nudum]. Gray 1870: 36, 37.
Plumarella Gray, 1870: 36. Studer 1887: 51. Wright and Studer 1889: xlix, 73, 74, 281. Versluys 1906: 13, 14. Kinoshita 1908a: 6–8. Kükenthal 1915b: 144, 145 [key to genus and species]; 1919: 340–343 [key to genus and species]; 1924: 255 [key to genus and species]. Diechmann 1936: 155, 156 [key to genus]. Bayer 1956: F220; 1961: 293 [ill. key to genus]; 1981: 936 [key to genus]. Bayer and Stefani 1988: 454 [key to genus]. Fabricius and Alderslade 2001: 244, 245. Cairns and Bayer 2004b: 448, 449 [key to western Atlantic species]; Cairns and Bayer 2009: 29, 39, 40. Cairns 2011: 7–9; 2016: 51, 52; 2018b: 39. Cairns and Wirshing 2018: 1, 11.

###### Type species.

*Gorgoniapenna* Lamarck, 1815; subsequent designation by Wright & Studer, 1889: 73.

###### Diagnosis.

Branching in one plane, pinnate, with branches close together in many colonies. Polyps biserial, alternate (rarely, opposite) or irregularly scattered; never in whorls or pairs. All eight rows of body scales present; adaxial surface usually has fewer scales; inner face of opercular scales with inconspicuous apical keel, or none; opercular scales aligned with marginals. Sclerites of coenenchyme (some species) as scales or warty radiates in lower parts of colony and inner cortex.

###### Remarks.

Genus bears accepted status in WoRMS Database (Cordeiro et al. 2019).

##### Plumarella longispina

Taxon classificationAnimaliaAlcyonaceaPrimnoidae

Kinoshita, 1908

**Figure 39.**
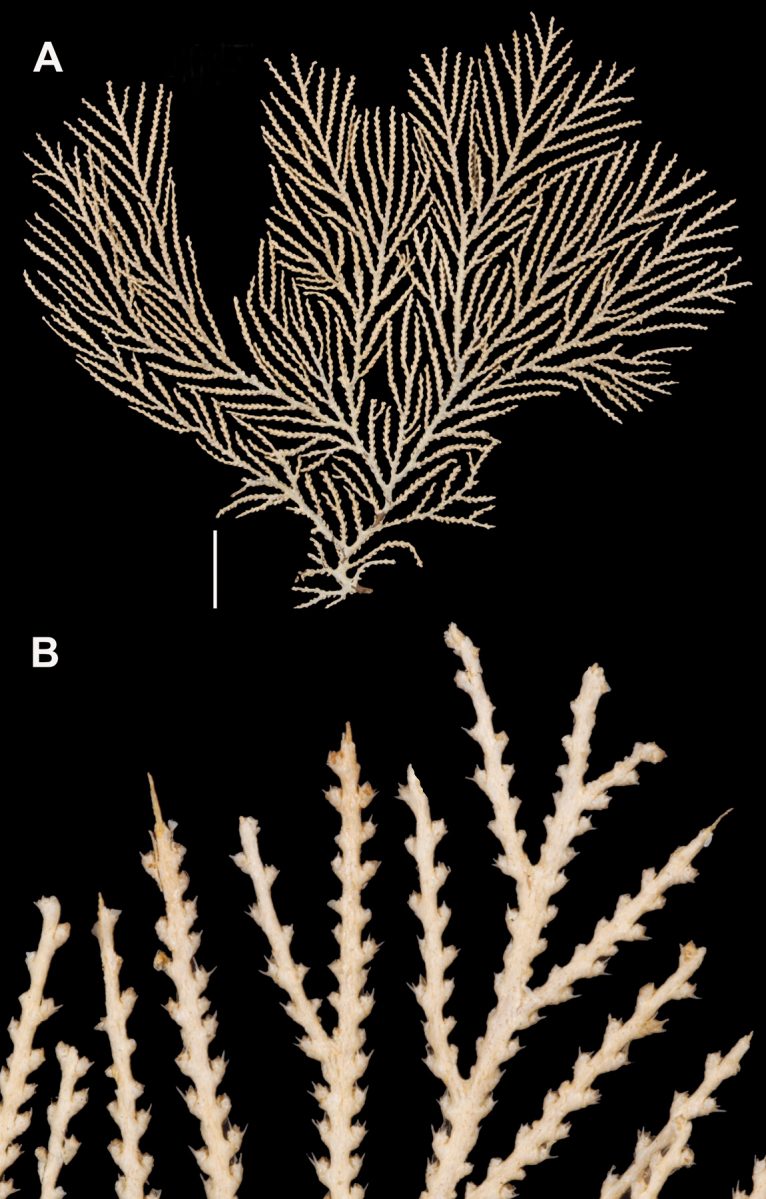
*Plumarellalongispina*, SBMNH 422394. **A** Colony, 14 cm tall × 15 cm wide **B** Branch tips. Scale bar: 2 cm (**A**).

**Figure 40.**
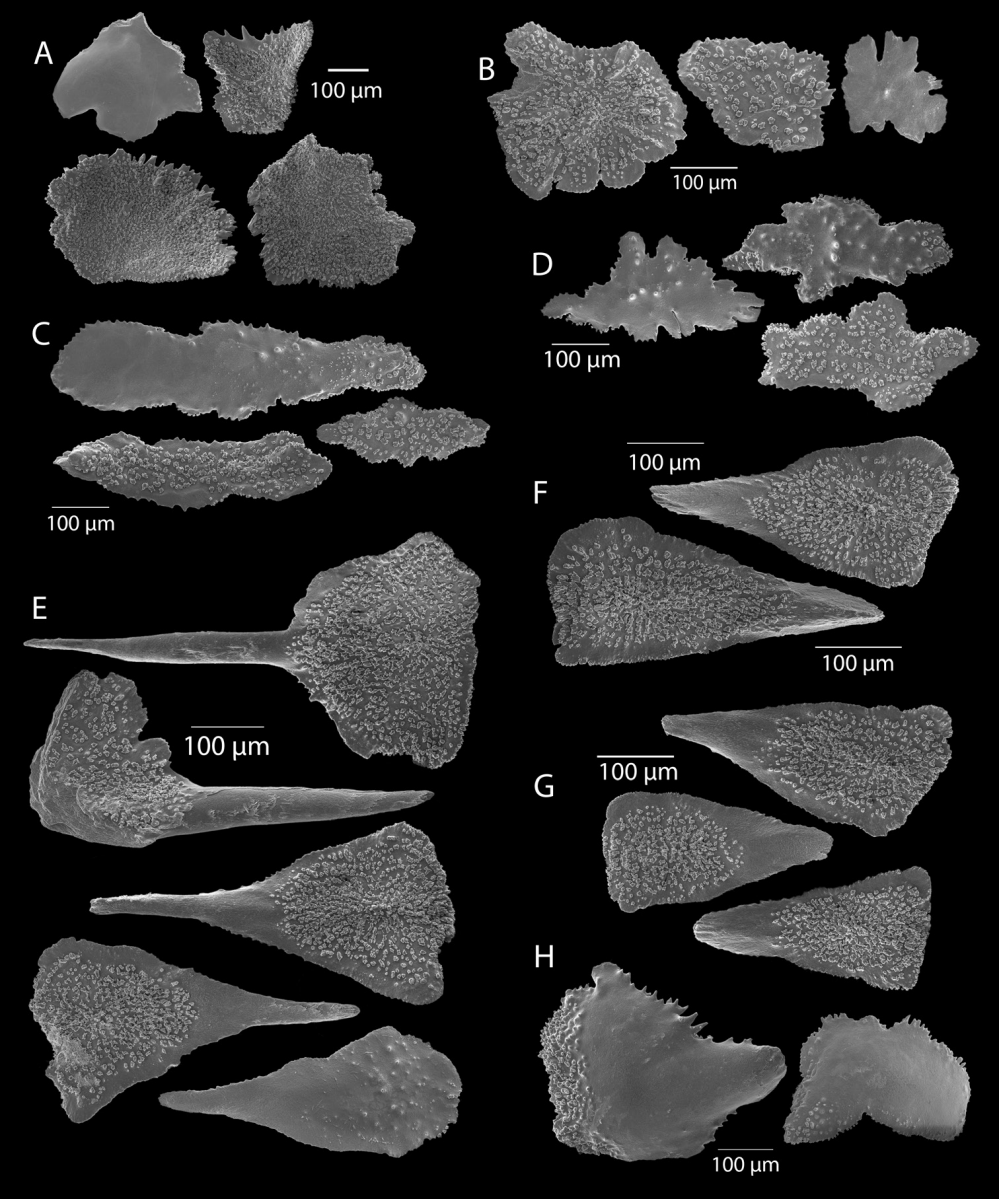
*Plumarellalongispina*, SBMNH 422394, SEM image. **A, B** Body wall scales **C** Flatter coenenchymal scales **D** Marginal scales **E** Marginal spinous sclerites **F–H** Opercular scales (worn). Compare/contrast these images with those shown in Cairns 2011 (Figure 5, *P.spicata*).

Figures 39A, B , 40A–H

Plumarella longispina Kinoshita, 1908a: 14, 15. Nutting 1909: 716. Kükenthal 1924: 260, 261.

###### Type locality.

N. Pacific Ocean, Japan, Honshu Island, Sagami Bay, Okinose Bank, 600 m.

###### Type specimens.

**Holotype**USNM 50117 [dry]; branch (from holotype), donated by Tokyo Imperial Museum; this material was examined. Main colony presumably still housed in collection at Tokyo Imperial Museum (all scientific and “natural materials” collections housed separately at what is now called the National Museum of Nature and Science); was unable to verify or confirm catalog number.

###### Material examined.

~33 lots (wet/dry) (see Appendix 3: List of material examined).

###### Description.

*Colony* (Figure 39A) exhibits dense, alternate, pinnate branching in one plane, leading to flabellate form. Main stem somewhat flattened, giving rise to alternate main branches at irregular distances; both main stem and branches may subdivide. Each main branch gives forth regularly alternate, slightly smaller branches that do not subdivide. Branchlets flattened, 1.5 mm thick (Figure 39B). Polyps small, short, cylindrical projections, 0.5 mm tall (to summit of operculum), 0.5 mm across, 1.5 mm apart; arranged laterally in two opposite rows on flattened stems, branches and branchlets; some polyps placed such that they project toward a front side of colony, with back of colony smooth; strictly alternate to strictly opposite in different parts of colony, with upper edge of one polyp ordinarily reaching to base of next one above. Polyp aperture pointed upward, slightly outward. Walls of polyps armed with sclerites; these conspicuous, flattened scales, vary greatly in size and form in different polyps. Color of colony (? alive) generally white; dry or in alcohol, dull creamy-white; some preserved colonies light grayish-brown, with surfaces of stem and branches being more distinctly gray. Sclerites (Figure 40A–H) quite varied in form, generally more or less flattened into scales; thin, cycloid. Key characteristic sclerite a flattened basal portion bearing on its distal edge long thorn-like processes (spines) projecting above margin of polyp (Figure 40E). Many scales ornamented with convex, ctenate margin. Surfaces of scales ornamented with evenly, closely distributed granules, irregularly placed nodular warts and occasional spines. Typical arrangement of scales on polyp wall is eight longitudinal rows, each row having roughly four scales in a ring; two proximal rings composed of broad curved scales with their distal convex edges ctenate, distal-most marginal ring composed of scales (with no keel), bearing prominent thorn-like, unwarted spines extending beyond end of operculum. Marginal spines usually number from two to six, two of which (abaxial) are often distinctly longer than the others. Operculum composed of eight irregularly shaped scales, not keeled, points of which often joined into spine-like processes (Figure 40F–H). Adcauline opercular scales reduced to narrow band, the antero-lateral processes from proximal rings of sclerites being the only ones that meet to complete the ring on abcauline side.

###### Etymology.

From the Latin, *longi*- = long and *spina*- = spine; long-spined, referencing the spinose marginal sclerites that extend beyond end of operculum on polyps.

###### Distribution.

Found off California coast between ~55–735 m. Of specimens examined, could not confirm that this species is found off the Oregon coast (thus far, all specimens examined were collected either from Baja California [Mexico] and California [USA] or Washington [USA]; it seems odd that it would skip an entire area between CA and WA). Based on material collected by staff of Olympic Coast National Marine Sanctuary (May, 2006 and July, 2008) that was examined, seen off northwest Washington coast at depths of at least ~208–309 m. Specimens from the genus have been taken in Alaskan waters (Bering Sea, etc.) in depths from 85–2514 m; collection data for these specimens can be found by doing a search of the online data base for the NMNH, Smithsonian, Invertebrate Collection. Listings of this particular species (by Wing and Barnard 2004; Heifetz et. al. 2005; and Stone and Shotwell 2007) mentioned in Cairns, 2011, could not be confirmed.

###### Biology.

Work by Lissner and Dorsey (1986) along Tanner and Cortes Banks and the Santa Rosa-Cortes Ridge area off southern California showed a depth range as follows: at depths <67 m the species is sparse, at depths ranging from 67–122 m the species is common to abundant, and at depths below 122 m, again becoming sparse. Deep-water video images taken by MBARI indicated the possibility of the genus (perhaps this species) being more common at greater depth (at least in some areas) than once thought.

In all specimens examined, only one had any other organism associated with it; on this specimen there appeared two anemones, both on branches near the tip. One, the larger of the two, is on the exposed axis. On this same specimen, on the area of branches just above the base, there appeared to be the anchor tendrils from the egg case of a shark. These tendrils are quite thin, but with the stiff curl they usually display. Egg cases were noticeable on specimens collected by OCNMS in May 2006. Colonies of this species are quite rigid, so it is likely that they provide good anchorage.

###### Remarks.

A key data point in the distribution of this species was Nutting’s specimen locality (1909): ‘Albatross’ station 4359, Point Loma light-house, 32°42'00"N, 117°14'00"W (N 85, E 9 miles), 191 fathoms (347 m). This specimen currently housed at NMNH (USNM 25429); specimen was examined.

In a comparison with a different species (from the Aleutian Islands, *Plumarellaspicata* Nutting, 1912), it presented marginal scales that were similar in shape to those seen in this species, but the spinous process of the marginal scales in *P.longispina* are much less ornamented. As well, all of the operculars in the species described here display areas of surface that appear very smooth and undecorated; in *P.spicata*, surface ornamentation is more prevalent, although perhaps not continuous along entire surface. Colony form of *P.spicata* (delicate and flimsy, more or less dichotomously branched), does not match what is seen for this species.

Unless there are very subtle differences, e.g., characteristics that might specify several subspecies, this species seemed to be one of the most abundant deep-water primnoids occurring in the California Bight (and elsewhere). Its overall colony form is quite distinctive, and easily recognizable. While appearing to be quite delicate, closer examination and handling indicated that it is actually fairly hardy. In the near future, an examination of all specimens in the SBMNH collection will have to be undertaken, with special attention paid to any feature(s) that could be assessed as a key characteristic that might show some degree of variability. The question arose as to whether there are transitional variations over the entire range of this species, and if so, whether those variations might subdivide the specimens, such that they point in the direction of distinct subspecies (or for that matter, species). Molecular studies on any of those groupings could add further clarity. However, it may be that this is simply an enormously successful species, thus very common, with adequate and successful dispersal abilities. Cordeiro et al. (2019) shows *P.longispina* with accepted species status.

##### Genus Primnoa

Taxon classificationAnimaliaAlcyonaceaPrimnoidae

Lamouroux, 1812

Primnoa Lamouroux, 1812: 188; 1816: 442. Johnston 1847: 171. Gray 1870: 44. Studer 1878 [1879]: 642; 1887: 49. Wright and Studer 1889: xlviii. Versluys 1906: 84–85. Kinoshita 1908a: 42. Kükenthal 1915b: 143 [key to genus]; 1919: 357–360 [key to genus]; 1924: 265–266 [key to genus and species]. Bayer 1956: 220, fig. 157, 1; 1961: 294 [illustrated key to genus]; 1981: 937 [key to genus]. Bayer and Stefani 1988: 454 [key to genus]. Cairns and Bayer 2005: 226–228 [revision and key to species]. Cairns and Bayer 2009: 30, 41, 42. Cairns 2011: 19.
Lithoprimnoa Grube, 1861: 174–175.
Lithoprimnoa : Lithoprimnoaarctica Grube, 1861, by monotypy.

###### Type species.

*Gorgonialepadifera* Linnaeus, 1767 (= *Gorgoniaresedaeformis* Gunnerus, 1763), by monotypy.

###### Diagnosis.

Dichotomously branched, arborescent form with polyps not arranged in whorls but closely crowded on all sides of branches and branchlets; polyps distinctly curved downward toward axis. Marginal scales eight, operculum strongly developed. Tentacles bear small, thorny rods.

###### Remarks.

This genus, in the form of *P.resedaeformis* (Gunnerus, 1763), has been known since the earliest days of science (1605), and *P.resedaeformis* (Atlantic species to which the species discussed here is most closely related) is one of the most often reported deep-water octocorals. It is likely that the Atlantic *Primnoa* were some of the very first deep-water octocorals to be seen and acknowledged (Cairns and Bayer 2005).

Genus, with accepted status in WoRMS Database (Cordeiro et al. 2019), mentioned here due to evidence provided by collection records examined (see Appendix 3: List of material examined), which showed a distributional range that includes the California Bight. Based on those collection records (NMNH), this is a deep-water form that has been collected off the California coast, from a southern location of La Jolla, San Diego to a northern location of Monterey Bay. As well, NMNH, OCNMS and MBARI, through collection and video records, also indicated sightings or collections off Oregon, Washington, British Columbia to the Gulf of Alaska, at 64-≥1000 m. This represents a substantial range but does include the entire California Bight region. Additionally, Cairns and Bayer (2005, 2009), along with Heikoop et al. (2002) and Risk et al. (2002), reported species of *Primnoa* from the subantarctic Pacific sector. Sánchez (in Gordon 2009), reported species of *Primnoa* off New Zealand coasts.

##### Primnoa pacifica

Taxon classificationAnimaliaAlcyonaceaPrimnoidae

Kinoshita, 1907

Primnoa pacifica Kinoshita, 1907: 232; 1908a: 42–45, text figs 8–9, pl 3, figs 19–20, pl 6, fig. 49; 1908b: pl 18, fig. 3; 1909: 2, 3, text fig. Wing and Bernard 2004: 24, fig. 15. Cairns and Bayer 2005: 233–239. Stone and Shotwell 2007: 72, 93, 107, in situ fig. 2.23. Whitmire and Clarke 2007: 152 (listed). Cairns and Bayer 2009: 30 (listed). Cairns 2011: 19.
Primnoa resedaeformis var. pacifica Kukenthal, 1915b: 146; 1919: 361–362. Aurivillius 1931: 295–296.
Primnoa japonica Verrill, 1922: 15 (nomen nudum).
Primnoa resedaeformis pacifica Kukenthal, 1924: 267, fig. 152. Heifetz et al. 2005: 132.
Primnoa resedaeformis forma pacifica Broch, 1935: 29–33, figs 17a-e, 18a; 1940: 20, 21. Naumov 1955: 66, pl 11, fig. 5.

###### Material examined.

No specimens in SBMNH collection (see Appendix 3: List of material examined).

###### Remarks.

Members of this genus display, in texture and color (when preserved in alcohol) that reminiscent of large-curd cottage cheese, arranged into branches. *P.resedaeformis* from the Atlantic is known to Canadian fishermen commonly as Seacorn or Popcorn coral. (On a first examination of preserved specimens, which were creamy yellow-white in alcohol, the appearance of popcorn immediately came to mind.) Information given here primarily focuses on *Primnoapacifica* typical; known distribution ranges from Honshu, Japan; California, north to at least the Aleutian Islands and Gulf of Alaska (Cairns and Bayer 2005). *P.pacifica* is known by some (anecdotal, via fishermen working in the Pacific) as Red tree coral (when living, the colony’s pink color is quite beautiful), as well as Seacorn or Popcorn coral.

This species has now been synonymized with *P.willeyi* Hickson, 1915, following work done by Cairns and Bayer (2005, 2009); this is shown in the WoRMS Database (Cordeiro et al. 2019), where it is considered in the Database as Primnoapacificavar.willeyi (Hickson, 1915).

Research staff at OCNMS originally believed that *Primnoa* occurred only on hard substrates (such as large boulders, and exposed bedrock) in areas of low turbidity, at a minimum yearly temperature of 3.7 °C, at depths of at least 9–800 m (Brancato et al. 2007). However, the OCNMS expedition in May of 2006 noted its location at several sites having muddy or sandy bottoms.

Verrill noted, in his original unpublished notes for the ‘Blake’ Expedition manuscript (transcribed by Bayer in personal notes but not published with the plates in Bayer and Cairns 2004) that in the deep sea, because of the “absolute stillness of the water,” many deep-dwelling forms exhibited extreme delicacy and fragility. He noted that numerous examples of the more delicate features occurred in the family Primnoidae. At depth, this species has been found with crinoids intertwined amongst its branches; it may further provide shelter within its branches for species of deep-water rockfish (Brancato et al. 2007). While Verrill noted (unpublished personal note transcriptions made by Bayer) that many of the deep-sea Alcyonaria are “phosphorescent” (bioluminescent), no recent information was found that could confirm/deny that characteristic for this species, or any other in the family.

The specimen mentioned in the Appendix 3: Other material, may be the southern-most report in eastern Pacific (USA) waters for a specimen of this genus and species. Occurring as far south as La Jolla, California, it may also be found further north into the California Bight, perhaps off the Channel Islands, in deep water. Of the thirteen genus records noted at CAS, the majority of specimens are from Alaska; their only record of this species is from the Sea of Japan. MBARI has records (provided by L Lundsten) for colonies known to belong in the Family Primnoidae, but most are not identified to genus or species. It would not be surprising if some of those specimens represent species within this genus, if not this species. As this manuscript was in preparation, a colony fragment (this genus and likely this species) was located (by myself and my research student, C Schaefer, in 2015) in material sampled from LACoMNH; fragment was found in fishing nets in 1981, set in SW Alaskan waters.

##### Genus Narella

Taxon classificationAnimaliaAlcyonaceaPrimnoidae

Gray, 1870

Narella Gray, 1870: 49. Deichmann 1936: 168. Bayer 1951: 41–43; 1956: F222; 1961: 295 (key); 1981: 937 (key); 1995: 147, 148; 1997: 511. Cairns and Bayer 2003: 618, 619; 2004a: 7–10. Cairns and Baco 2007: 392, 393 [a more complete synonymy and discussion]. Cairns and Bayer 2008: 84–86; 2009: 2, 30, 31, 43. Cairns 2012: 14. Taylor and Rogers 2017: 4. Cairns 2018a: 20, 21; 2018b: 19. Cairns and Taylor 2019: 1–15.
Stachyodes Wright and Studer in Studer, 1887: 49; 1901: 40. Wright and Studer 1889: xlvii, 53. Versluys 1906: 86–88. Thomson and Henderson 1906a: 35. Kinoshita 1907: 233; 1908a: 45–47. Thomson and Russell 1910: 142. Kükenthal 1912: 59; 1915b: 152; 1919: 452–456; 1924: 308, 309.
Calypterinus Wright and Studer in Studer, 1887: 49. Wright and Studer 1889: xlviii, 54.
(?) Calyptrophora (pars) Verrill (in Bayer and Cairns 2004).

###### Type species.

*Primnoaregularis* Duchassaing & Michelotti, 1860.

###### Type locality.

North Atlantic Ocean, Caribbean Sea, St. Lucia, south of 13°36'27"N, 61°03'36"W, 514 m.

###### Type specimen.

**Neotype** of type housed at NMNH (USNM 49385, wet); not examined.

###### Material examined.

None housed at SBMNH.

###### Diagnosis.

Colonies of moderate size (to 50 cm height), branched dichotomously or pinnately (some few trichotomously) in single plane, or unbranched. Polyps conspicuous, facing downward, in discrete whorls or pairs, non-retractile. Axis continuous; strongly calcified, especially in lower branches; generally grey to black, sometimes with metallic sheen; down center of axis (longitudinally grooved) is solid core of calcareous material. Base a discoidal holdfast, for attachment to solid substrates. Sclerites are scales, on polyps, usually numbering sixteen to eighteen on each polyp (not counting tentacular sclerites), arranged in three or four pairs of large unfused abaxial body wall scales that partially encircle polyp, but rarely meet adaxially; arranged so as to have definite pattern and number. With adaxial buccal scales commonly present, one well developed buccal in each row. Operculum consists of eight (four pairs) generally triangular scales, each with distinct longitudinal medial keel on inner surface, with corresponding trough on outer surface. Tentacles can contain few to numerous, minute, flat rodlets; coenenchymal sclerites elongate or elliptical scales, often with tall longitudinal keels.

###### Distribution.

Exclusively deep water (55–4,594 m), found worldwide (Cairns and Bayer 2008; 2009). The genus is noted (Cairns and Baco 2007) as having the second deepest location record of all primnoid genera (4,594 m in the Gulf of Alaska). In addition to species from the Atlantic, there are some 23 species recorded (Cairns and Baco 2007) from regions in the Pacific (Alaska, Japan, Hawaiian Islands, Indonesia and eastern Pacific). Also, a few species are recorded from either the SW Indian Ocean, the Galápagos Islands or off Antarctica (Cairns and Baco 2007). Cairns stated (2007b) that species of *Narella* have been found along the southern California coast, from both San Marcos Seamount (2,193 m), and Rodriquez Seamount (664 m). These specimens were very fragmented and could not be definitively identified, but each one may represent an undescribed species. Some 54 named species are considered valid within this genus currently, as seen in the WoRMS Database, listed by Cordeiro et al. (2019).

###### Biology.

In Studer’s 1894 description of *N.ambigua*, he discussed the presence of an annelid worm from family Eunicidae Berthold, 1827 that had established itself on the coenenchyme. It apparently sought shelter under the wing-shaped extensions, in a space (a tunnel of sorts) produced by the greatly enlarged basal scales of each of the neighboring polyps. In personal note transcriptions (unpublished) made by Bayer, Verrill had outlined thoughts he had concerning the deeper water gorgonians. Regarding the annelid worm found in Studer’s specimen, Verrill (unpublished personal note transcriptions made by Bayer) discussed a comparable situation and referred to Studer’s 1894 examination.

###### Remarks.

The genus is presented here; based on collection records examined (NMNH), there is indication of a distributional range that includes the California Bight. Based on those collection records (NMNH), this is a deep-water genus that has been collected (if only as fragments) several times off the southern California coast (Cairns 2007b).

According to Cairns and Baco (2007) and Cairns (2007b), there were some 38 recognized species (that number has increased, according to Cordeiro et al. 2019), making this a species-rich genus; in fact, it is said to be the most prolific of the primnoid genera (Cairns 2007b). Of those, there may be at least a few species that could potentially be found in or near the California Bight; *Narellaambigua* Studer, 1894 is one species that might yet be found in the Bight. CAS has seven records of this genus (none identified to species), coming from Hawaii and Alaska. MBARI has posted on-line images of those in this genus found on Davidson Seamount, photographed at depths of 2,669 and 3,079 m. Only one specimen identified to this genus has been recorded as having been collected by MBARI staff, but there are a few additional video observations. This one collected specimen was taken in the general area slightly north and west of San Miguel Island, California Channel Islands. Of interest is a specimen housed at NMNH; from California, Rodriquez Seamount, W of San Miguel Passage, 34°02'17"N, 121°02'49"W, 662 m; coll. unknown, date unknown; USNM 1027059 [wet]. The MBARI specimen and the one at NMNH appeared to be from the same collection event. It is the shape and sculpturing of the abaxial body wall scales that are the best means to identifying a species in the genus; however, finer details regarding sculpturing of scales can only be seen with SEM. Further work with unidentified species housed at CAS and NMNH should be undertaken.

##### Family Isididae

Taxon classificationAnimaliaAlcyonaceaIsididae

Lamouroux, 1812

###### Diagnosis.

Axis distinctly segmented, composed of alternating purely horny (gorgonin) nodes and nonscleritic calcareous, mostly solid, internodes (in some, hollow); internodes may be colored, quite smooth or with small projections or ridges. Base may be either a root-like calcareous structure for anchoring colony in soft substrate or a basal disc for attachment to hard object. Colonies whip-like, profusely branched, bushy or fan-like, with polyps retractile (or not). Majority of species in family found in deeper waters; all members of family commonly called Bamboo coral.

##### Genus Acanella

Taxon classificationAnimaliaAlcyonaceaIsididae

Gray in Wright, 1869

Acanella Gray in Wright, 1869: 23–26. Gray 1870: 16. Wright in Studer 1887: 44. Nutting 1910b: 14. Kükenthal 1915a: 117, 119; 1919: 573; 1924: 414. Deichmann 1936: 243. Bayer 1956: F222; 1981: 941 (key). Bayer and Stefani 1987a: 51 (key); 1987b: 941 (key).
Isidella Muzik, 1978: 737.

###### Type species.

*Mopseaarbusculum* Johnson, 1862.

###### Type locality.

Atlantic Ocean, Canada, Nova Scotia, Sable Island, ~43°56'10"N, 59°56'10"W, 503 m.

###### Type specimen.

**Type** (status not researched); YPM 4744 [dry]; as *Acanellanormani* Verrill, 1878a, now considered synonymous with Acanella (Mopsea) arbuscula (Johnson, 1862).

###### Material examined.

No specimens of this genus in collection at SBMNH.

###### Diagnosis.

Colonies densely or openly bushy, moderate-sized (no more than 20 cm); usually anchored in soft substrates (ooze or fine sand) by lobate, root-like holdfast, in deep water. Colonies generally larger and compressed (to one meter in height) when attached to hard substrates. Internodes white; nodes generally some shade of brown. Branched in whorls (three to six, at least in upper parts) from horny nodes; internodes solid, shorter (up to 2.0 cm). Polyps generally non-retractile, often prominent, columnar; coenenchyme thin. Sclerites of polyps mostly spindles; some flattened blunt rods, with fine prickles or low warts. Larger spindles and/or rods in body wall; sometimes rods conspicuously projecting between bases of tentacles. Small, slightly flattened, sometimes thorny, rods and/or double stars in pharyngeal walls.

###### Etymology.

While members of this genus are commonly referred to as a type of Bamboo coral, no discussion of genus name derivation was found. Genus is listed with accepted status by Cordeiro et al. (2019).

###### Distribution.

Deep water, throughout all oceans, based on an examination of collection records for specimens housed at various institutions (MBARI, NMNH, CAS).

###### Biology.

Verrill (unpublished personal note transcriptions made by Bayer) stated that most of the deep water Alcyonaria are bioluminescent; “among the ‘phosphorescent’ gorgonians, the abundant deep-sea species, *Acanellanormani* Verrill, 1878 was very ‘phosphorescent.’ It is also very well protected by sclerites and has a highly developed root-like branching base for anchorage in the deep-sea ooze. This has allowed it to become one of the commonest and most widely diffused of all deep-sea genera.”

From examinations of recent deep-water video and digital stills (MBARI), species in this genus are usually seen on a muddy/sandy soft bottom. *Acanelladispar* Bayer, 1990 (a species that was described from material taken in Hawaii, and thus, found in the Pacific Ocean) is the only species noted (thus far) that inhabits a hard bottom and has a stout trunk.

###### Remarks.

Discussion of this genus included as there are reports of unidentified species (noted by MBARI in collection/video records undertaken by them) found north of the California Bight. It is not certain what, if any, species from this genus occur within the Bight, geographically lying some distance south of MBARI’s usual study locations. However, the California Bight has not been fully explored specifically for deeper water gorgonian forms; there is the possibility of species from this genus being found within it.

Andrews et al. (2005) discussed a specimen of this genus collected off San Francisco, California that was used in an age determination study of a gorgonian colony, and MBARI (posting on-line) displayed an image of a specimen, identified to this genus, sighted on Davidson Seamount, at a depth of 1,682 m (photograph taken 28 January 2006). From the MBARI data lists, roughly four specimens collected have been identified to this genus. Several other observations, without collection, have also been recorded in the area extending from southwest of Morro Bay to off the coast of Oregon (lat./long range of 35/36–45°N, 122–130°W). As for the total number of species within this genus, most are from the Atlantic; Cordeiro et al. (2019) in the WoRMS Database list 13 species. CAS has five specimens recorded, three from Japan and two from USA, Massachusetts, off Martha’s Vineyard, while the NMNH has quite a few specimens (~305), from either Hawaii, Japan, the Philippines, or Indonesia; however, the vast majority are from the North Atlantic. Pacific Ocean species include the previously mentioned *A.dispar* Bayer, 1990 as well as *A.sibogae* Nutting, 1910b and *A.weberi* Nutting, 1910b. Further expeditions, with collection and study, need to be done to determine if species from this genus occur within the California Bight.

##### Genus Isidella

Taxon classificationAnimaliaAlcyonaceaIsididae

Gray, 1857

Isidella Gray, 1857 [1858]: 283; 1870: 14. Verrill 1883: 13. Studer [and Wright] 1887: 44. Kükenthal 1915a: 118; 1919: 564, 783; 1924: 414. Deichmann 1936: 239. Madsen 1944: 8. Bayer 1956: F222; 1981: 941 (key). Carpine and Grasshoff 1975: 107. Bayer and Stefani 1987a: 51 (key); 1987b: 941 (key). Bayer 1990: 207. Etnoyer 2008: 543. Brugler and France 2008: 126–127. Watling et al. 2011: 76, fig. 2.11. Dueñas et al. 2014: 20. Cairns 2018a: 37.
*Isis*. G. von Koch, 1887: 90 [description of *Isis neapolitana* Koch (= Isidellaelongata [Esper, 1788])].

###### Type species.

*Isis elongata* Esper, 1788.

###### Type locality.

Generally, eastern North Atlantic; likely, Mediterranean Sea.

###### Type specimen.

Location of type specimen unknown.

###### Material examined.

2 lots (see Appendix 3: List of material examined.)

###### Diagnosis.

Colonies sparsely branched from horny nodes, dichotomously (at ~30–35° angle; also trichotomously or lateral), generally in one plane, thus colony usually open, flat and spreading; a candelabrum shape possible. Internodes long, with axis in preserved colonies white; axis of nodes orangey-gold/brown; coenenchyme colorless. Branching not in whorls; branches moderately slender; distance from one branch to next (thus from node to node, establishing internode length) long, 3.5–4.0 cm; calcareous internodes hollow (distal tips; solid at proximal ends), longitudinally grooved, straight (or nearly so); horny nodes three-pronged, 3.0–5.0 mm tall at joints of older branches, but a simple cylinder (~1.0 mm tall) at joints of younger branches. Base of main stem a calcareous root, lobate, for anchoring in soft substrate or discoid, calcareous holdfast for anchorage on hard substrate. Polyps non-retractile and cylindrical. Sclerites of polyps mostly long rodlets that do not project between bases of tentacles; or stout, slightly prickly needles. Verrill (unpublished personal note transcriptions made by Bayer) made reference to “girdled ellipses,” which are elongated scales with rounded ends having a notable median constriction or emargination on each edge; these sclerites are normal in all Isididae.

###### Etymology.

All members of this genus are commonly called Bamboo coral, but no discussion of exact derivation could be found; genus has accepted status in the WoRMS Database (Cordeiro et al. 2019).

###### Distribution.

Deep water, likely worldwide; at depths averaging ~1,000 m (determined from collection records of various institutions, such as MBARI and NMNH).

###### Biology.

Can grow to very large size, perhaps able to attain great age (Andrews et al. 2005). Despite the calcareous nature of the internodes, as is true of many deep-sea gorgonians, species in this genus can be somewhat delicate and fragile. Quieter waters of the deep sea likely allow for the larger size.

###### Remarks.

Inclusion of the genus reflects locality data for the few collection and video records made by MBARI and NMNH off southern California. Of particular interest is USNM 1082174; specimen collection by D Clague (MBARI) on ‘Tiburon’ dive #630, 16 October 2003 (see Further remarks, below). No sclerite preparations could be done for specimens in SBMNH collection, as no coenenchyme tissue is present.

There are some six species recognized in this genus, according to Cordeiro et al. (2019); at least two are from the Atlantic. The species described by Bayer (1990), *Isidellatrichotoma*, Etnoyer (2008), *Isidellatentaculum*, and Cairns (2018a), *Isidellatenuis*, are confirmed from the Pacific. As access to deeper areas becomes more common, it is certainly possible that new species will be found.

Of specimens examined at CAS, none were identified to species. One specimen came from California, Humboldt County, two were from Oregon and two were from Alaska. None (as able to determine) are recorded in web-posted MBARI images, but some two dozen-plus specimens have been sampled (with even more video observations made) by MBARI in a region encompassing an area just west of San Miguel Island in the northern California Channel Island group extending northward to an area SW of San Francisco (lat./long range = 34–37°N, 121–123°W). From the collection at NMNH, the one specimen (identified as *Isidellatentaculum* Etnoyer, 2008) is of interest; taken off California at Rodriquez Seamount, 34°01'26"N, 121°05'59"W, 846.9 m; USNM 1082174. The holotype for this species, is USNM 1076658, collected by P Etnoyer in the Gulf of Alaska, on Dickins Seamount, 7 August 2004 (see Etnoyer 2008). A paratype of this species is found in the SBMNH collection, SBMNH 369349 (Gulf of Alaska, Welker Seamount). Only further study with collection can determine how much further south members of this genus can range, and whether or not they are present in the California Bight.

##### Genus Keratoisis

Taxon classificationAnimaliaAlcyonaceaIsididae

Wright, 1869

**Figure 41.**
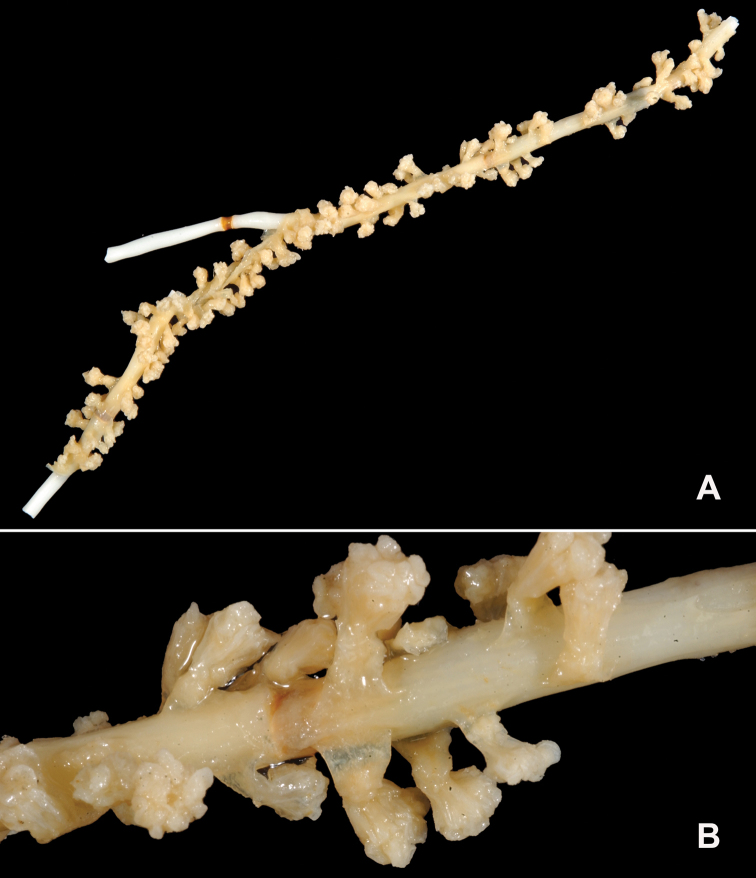
Genus *Keratoisis*, SBMNH 422980. **A** Branch fragment; coenenchyme thin, translucent yellow, easily coming off underlying axis; the fragment measures ~15 cm long **B** Close up of polyps and very thin coenenchyme on branch fragment.

**Figure 42.**
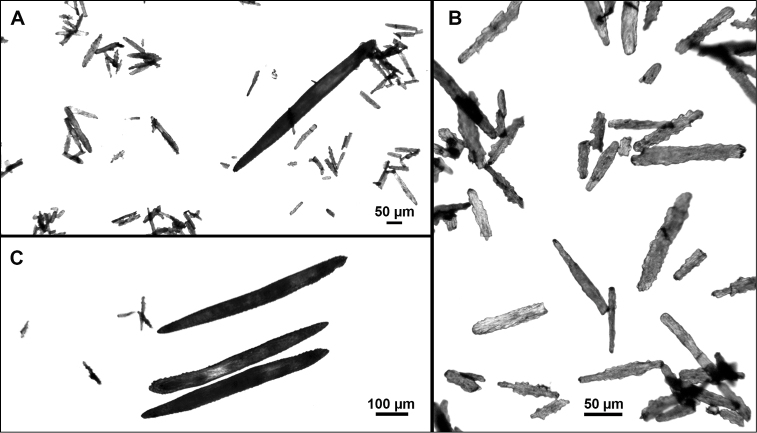
Genus *Keratoisis*, SBMNH 422980, light microscopy arrays. **A** 4× magnification, showing sclerites, most notably very long needle form **B** Array of shorter needles, 10× magnification **C** Image specifically highlights very long needles characteristic of species in genus *Keratoisis*. Long needle-like sclerites range from 620–775 µm in length, while very small spindles average ~80 µm.

**Figure 43.**
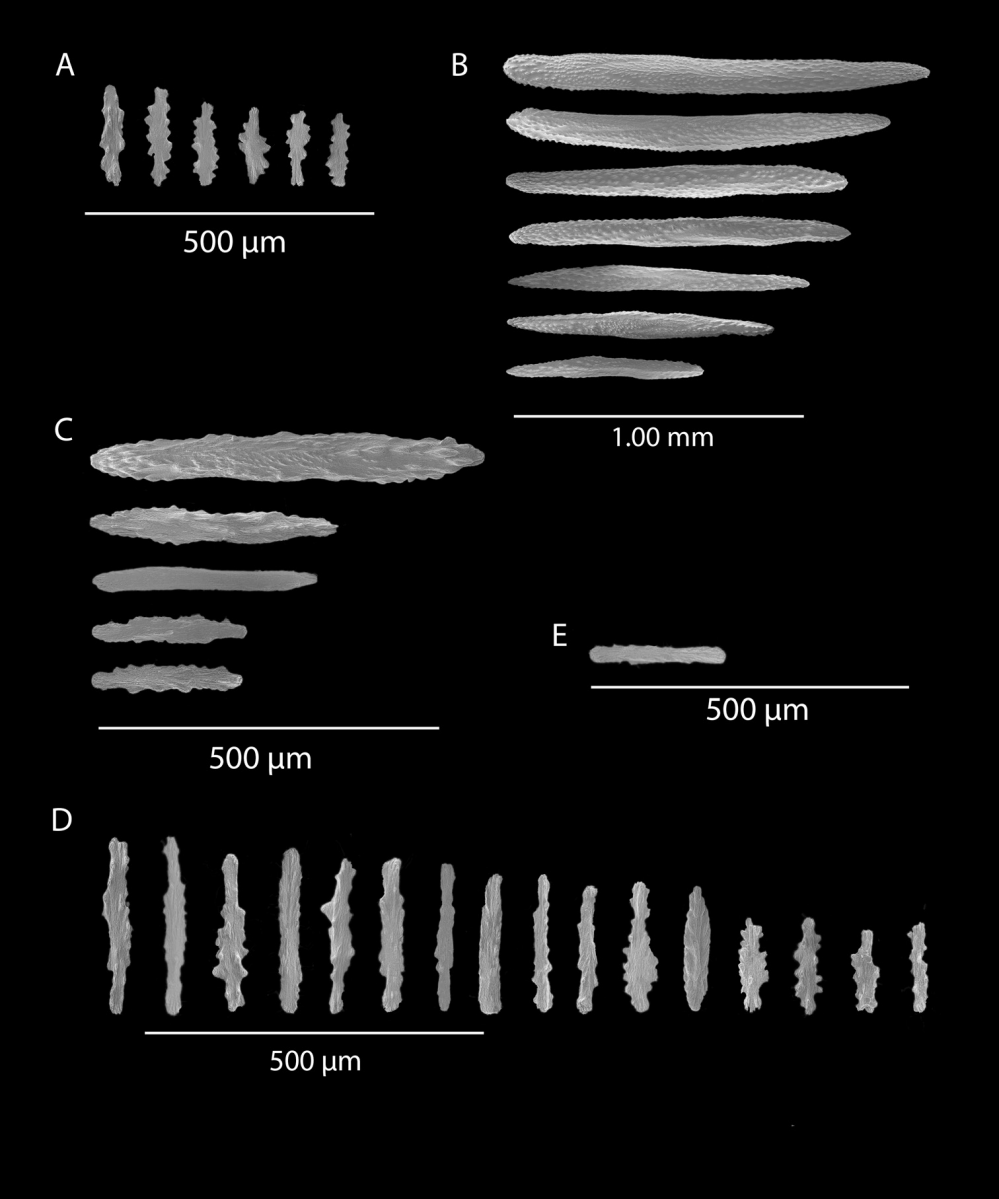
Genus *Keratoisis*, SBMNH 422980, SEM image. **A, C–E** Moderate-length spindles **B** Very long, fusiform spindles (needles) characteristic of genus.

Figures 41A, B , 42A–C , 43A–E

Ceratoisis Wright, 1868: II, p. 427 (name only). Verrill 1883: 10. Wright and Studer 1889: 26. Hickson 1907: 5. Nutting 1908: 570.
Keratoisis Wright, 1869: III, p. 23, 24. Gray 1870: 18. Studer 1878: 662. Wright [and Studer], 1889: xlii-xliii, 25, 26. Thomson and Henderson 1906b: 429. Nutting 1910b: 9. Kükenthal 1915a: 117, 120, 121. Molander 1929: 78. Grant 1976: 30.
(= Bathygorgia Wright & Studer, 1889: 691; Cairns and Bayer 2005, listing only).

###### Type species.

*Keratoisisgrayi* (Wright, 1869). Some few years ago, UNESCO-IOC Register of Marine Organisms proposed the possibility of *Keratoisisornata* Verrill, 1878 being a synonym of the type. Information provided on World Register of Marine Species (WoRMS) indicated that that synonymy is now accepted (Cordeiro et al. 2019).

###### Type locality.

Specific locality of type unknown; generally, bathyal, from NE to NW Atlantic Ocean; also Mediterranean Sea.

###### Type specimen(s).

Location of the type species could not be determined.

###### Material examined.

~3 lots (see Appendix 3: List of material examined.)

###### Diagnosis.

Colonies (Figure 41A) branched (few and distant, ~5.0 cm from one branch to next), with branches arising at nearly 90° angle, on same side, or opposite(?); near a node or from middle to end of long (4.0–5.0 cm), calcareous internode, then slightly curving; no secondary branching; some unbranched; generally uniplanar. Base can be either root-like calcareous structure for anchoring into soft substrate or a basal disc for attachment to a hard substrate. Axis as seen in the family; internodes calcareous, white, not composed of fused sclerites, hollow (often) or solid; and purely proteinaceous, horny, shorter (2.0 mm tall), reddish-brown to dark brown nodes, alternating with internodes. Overall color of colony (preserved) creamy yellowish; coenenchyme translucent yellow. Polyps (Figure 41B) cylindrical, height between 4.0–8.0 mm; not retractile. Polyps irregularly arranged, but with tendency toward biserial arrangement; in general, somewhat curved, distal part of polyp body with eight longitudinal rows of spindles and needles, some projecting beyond tentacles. Tentacles of polyp form a rounded top, like a mushroom, with individual tentacles usually visible. Distance between polyps no more than 1.0 cm but usually less. Coenenchyme very thin, transparent; straw-yellow in specimens examined. Sclerites (Figure 42A–C, 43A–E) generally long, fusiform spindles; some (Figure 43B) very long (needles) and others (Figure 43A, C–E) more numerous, of moderate length, in coenenchyme and polyp bodies; those in coenenchyme not always obvious; polyps armed with eight-plus, needle-like sclerites (largest), often (not always) projecting beyond tentacles as sharp marginal spines between bases of tentacles, coming from eight longitudinal rows of spindles and needles. Sclerite surfaces seemingly smooth, or (if present) with dense low warts, in parallel. Stellate forms seen in pharynx. Sclerites colorless to light tan, depending on species.

###### Etymology.

No clear derivation for this genus name was found. All members of this genus are referred to as species of Bamboo coral. Genus *Keratoisis* is accepted; WoRMS Database (Cordeiro et al. 2019) shows the spelling variation, *Ceratoisis* Verrill, 1883, as synonymized with *Keratosis* Wright, 1869.

###### Biology.

It had been noted (Verrill, 1922) that this genus included some of the largest known species of the family; specimens of *K.ornata* (now *K.grayi*), from considerable depth, on the banks off Newfoundland and Nova Scotia, can be ~ four feet high. As well, this genus is composed of species that may live to considerable age. Andrews et al. (2005) stated that age for some of these coral colonies may exceed 200 years.

###### Remarks.

Discussion of the genus included here, as several fragmented specimens in the collection of SIO and NMNH were collected off California (see Appendix 3: List of material examined and discussion in this section). The speculation is that this genus may be seen throughout much of the Pacific Ocean, at depth, based on collection location data found via on-line databases, etc. Several species that might be of interest with regards to the California Bight could include *K.paucispinosa* (Wright & Studer, 1889) ranging from Alaska to Hawaii, *K.philippinensis* (Wright & Studer, 1889), which is generally a western Pacific form, ranging from Russia to Indonesia and *K.flabellum* (Nutting, 1908), which apparently has only been recorded from Hawaii. *K.profunda* (Wright, 1885), recorded from Alaska and Japan (as noted by B Wing and G Williams in Andrews et al. 2005), was at first thought to be the only species in this genus actually listed for the northeastern Pacific Ocean. However, *K.profunda* is no longer recognized as belonging to this genus, having been accepted as *Bathygorgiaprofunda* Wright, 1885 (Cordeiro et al. 2019).

Of the approximately fourteen specimens identified as belonging to this genus, housed in the collection at CAS, roughly half are from California; the other half from either Alaska or Hawaii. Of these fourteen, most are not identified to species, but of those that are, three of the four species mentioned above are listed, with the two records from California that have species identification listed as *K.flabellum* and *K.philippinensis*; no opportunity to verify those identifications. The southernmost California records seen previously were from Monterey Bay and from the now extensively studied Davidson Seamount; one other specimen from this genus collected/photographed by MBARI (shown on a public website) at Davidson Seamount noted it as being collected at 1,455 m. Searching Excel data sheets from MBARI (provided by L Lundsten in 2008), three dozen or so specimens have been collected over the last few years, but there are many more video observations (many of those will be multiple observations of the same organism) that have been identified as belonging to this genus. NMNH has records of specimens belonging to this genus from both Oregon and Alaska, and of note are several other specimens: those from Fieberling Guyot, 32°26'00"N, 127°47'36"W, 490 m, 16 October 1990; USNM 94443, and those from Rodriquez Seamount, 33°57'12"N, 121°08'41"W, 1840 m, 14 October 2003; USNM 1027077, both of these areas off the California coast.

##### Genus Lepidisis

Taxon classificationAnimaliaAlcyonaceaIsididae

Verrill, 1883

Lepidisis Verrill, 1883: 18 [pars]. Studer 1894: 62. Kükenthal 1915a: 117, 119; 1919: 569 [pars]; 1924: 417 [pars]. Deichmann 1936: 240–242 [pars]. Bayer 1956: F222 [pars]. Muzik 1978: 737. Grasshoff 1986: 30. Bayer 1989: 198, 201. Bayer 1990: 204, 205.
non Lepidisis Grant, 1976: 30 (= Keratoisis).
Acanella Verrill, 1883: 13 [pars]. Wright and Studer 1889: 29 [pars].
Bathygorgia Wright, 1885: 691 (type species, Bathygorgiaprofunda Wright, 1885 by monotypy). Wright and Studer 1889: 32.
Ceratoisis Wright & Studer, 1889: 26 [pars]. Hickson 1907: 5 [pars]. Kükenthal 1915a: 120 [pars]; 1919: 585 [pars]; 1924: 423 [pars]. Deichmann 1936: 246 [pars].
Keratoisis Bayer, 1956: F222 [pars]. Tixier-Durivault 1966: 434 [pars]. Grant 1976: 15 [pars].

###### Type species.

*Lepidisiscaryophyllia* Verrill, 1883; subsequent designation Kukenthal, 1915a (*L.caryophyllia* accepted species; proposed synonymy for *Lepidisisvitrea* Verrill, 1883 has been accepted as shown in WoRMS Database, Cordeiro et al. 2019).

###### Type locality.

Generally, northern and western Atlantic Ocean; bathyal.

###### Type specimen(s).

Location of type unknown.

###### Material examined.

No specimens of this genus in collection of SBMNH.

###### Diagnosis.

Colonies simple, unbranched, or (rarely) sparsely branched from horny nodes; internodes hollow. In overall shape, whip-like, often exhibiting spiral growth form. Base root-like, for anchorage in deep-water bottoms of soft ooze or fine sand. Polyps non-retractile. Sclerites of polyps projecting needles and elongate scales.

###### Etymology.

No explanation was found for the rationale behind the naming of this genus; they are however, commonly called Sea whips.

###### Distribution.

A deep-water genus, likely found worldwide.

###### Biology.

The apparent fragile and delicate nature of many deep-sea species of gorgonian in this suborder, including this genus, may demonstrate the relaxation of certain selection pressures in the deep sea, as proposed by Childress (1995) for deep-water forms. As well, many deep-water forms of Alcyonaria can be bioluminescent. This was certainly true for species described by Muzik (1978) seen in Hawaiian waters; further studies should reveal whether that feature is true of other members in this genus.

###### Remarks.

To date, there are approximately a dozen species recognized and accepted within the genus (Cordeiro et al. 2019); brief discussion is included based on location data for specimens collected (or at least noted) by both MBARI and NMNH. Both institutions have specimens that were either collected or note locations that put them in close proximity of, if not actually in, the California Bight, but only a very few described species have potential for being located within the region (although new species are certainly possible as deep-water sites are further explored). There are two specimens of interest housed at NMNH: one from California Channel Islands, San Nicolas Island, ~40 miles SW of the island, 32°31'08"N, 119°42'10"W, 950 m; coll. J Ljubenkov, no date given; USNM 59821 [wet], the other from California, Fieberling Guyot, W of Channel Islands, 32°27'36"N, 127°49'30"W, 640 m; coll. un-known, via submersible ‘Alvin’, 14 October 1990; USNM 94447 [wet]. A posted MBARI image showed a pink specimen from Davidson Seamount, at 2,683 m. MBARI data records indicated that approximately a dozen different samples have been taken, classified as belonging to this genus, with many more video-recorded sightings, in the vicinity of 32–35–37°N, 121–122–123°W. CAS has only three specimens, none of them from California waters; none of these specimens have been identified to species. This is a genus that requires further study; only with collection events south of Monterey Bay, in and near central California or some distance west of the northern California Channel Islands, will we know the extent to which members of this genus are present within the California Bight.

The description given by Studer, 1894 for *Lepidisisinermis* originally did not seem to fit with general characteristics ascribed to members of the genus. He did, however, in his description, mention similarities with Ceratoisis (Keratoisis) nuda Wright & Studer, 1889; this was later recognized as synonymous with *Lepidisisnuda* (Wright & Studer, 1889); the species *L.inermis* has branching from the internodes. It would appear that in some instances, sparse branching does occur in some species within the genus *Lepidisis*.

## Discussion (summation of Parts I, II, and III)

Originally, SBMNH inhouse listings indicated that no more than a few dozen (at most) gorgonian coral species existed in the California Bight. This comprehensive study of the holdings in the SBMNH Invertebrate Research Collection, bolstered by a significant incorporation of specimens collected by the Allan Hancock Foundation (AHF) ‘Velero’ Expeditions of 1931–1941 and 1948–1985, donated to the SBMNH, revealed that central and southern California temperate water species are far more numerous and diverse than previously thought, with most not easily identifiable to species by cursory examination. This diversity is not surprising, in light of the fact that the California Bight is an area rich in species, the result of three major bodies of water all convening off the southern and central California coast, along with the presence of many different microhabitats (coastal shallow, subtidal, deep water, long coastlines with scattered bays, as well as several channel basins with islands, ridges, canyons and basin-like depressions). The array of gorgonian coral specimens housed at SBMNH, while not large in total number, well represents this broad diversity, with some species revealing wide ranges of distribution within this geographic region.

Specimens in the SBMNH collection displayed one or the other of the two basic body configurations seen in gorgonians (branched and fan-shaped or slender and whip-like), revealed over a wide range of species. Uniplanar configuration is a possibility for fan-shaped colonies, but many species with extensive branching displayed a more three-dimensional aspect to their colony shape. This is an accurate reflection of the environmental conditions under which many live. In examinations of a number of colonies (of various species) their plasticity was very evident. This aspect of gorgonian biology implied that a more flexible body form was possible than was indicated in older literature where descriptions were given of colony form for a species. A species, while described as being “always in one plane,” was often rarely so. All specimens examined were identified to species whenever possible, and species likely to occur in the CA Bight have all been considered. Taxonomic listings of higher order taxa were provided where applicable and simple taxonomic keys to families were included for each of the suborder designations; keys to genera, and most species, were not. The goal of this three-part work was to provide a comprehensive review that would enable most field researchers to identify most gorgonians encountered in California waters. Consideration had be given to the fact that the SBMNH collection is composed of more than just the typical, commonly encountered species. Accompanying the discussion of a few problematic genera (genera that presented taxonomic questions where there are multiple species present in the SBMNH collection, most notably the genus *Swiftia*, Part III), a key to species is provided. In some instances, no previous description existed for the conditions and characteristics seen in a specimen. This was particularly true of several thread- or whip-like forms. One of these thread-like gorgonians was described earlier (Horvath 2011), and a second thread-like form had to be introduced in Part II as a new species (*Eugorgialjubenkovia* sp. nov.).

Understanding the significance and variability of sclerites continues to be essential to the identification of alcyonacean gorgonian corals. While it takes time to become familiar with these structures, they are foundational to species identification. The best source for identifying both common and more unusual forms of sclerite continues to be the work by Bayer et al. (1983), but as more, and unusual, species are discovered and described, new sclerite forms will need to be added to the listing of sclerite shapes (such as the “double-dunce cap” proposed for *Chromoplexauramarki* or the “tardigrade-like” spindle seen in *Muriceafruticosa* in Part II).

The “red whips” (most from family Plexauridae) were of particular interest. While at times difficult to link each of several different groups (red and “whip-like”) to species previously named, it ultimately required that California “red whips” be divided between at least two families and three or four different genera and species. These “red whips” continue to be a focus of study. Likewise, the entire genus *Thesea*, as found in the California Bight, presented the same whip-like body form, but this genus presented several additional challenges with regards to taxonomy, largely due to the fact that the genus had been studied far more extensively in the Atlantic (Deichmann 1936), but little studied in the eastern Pacific. In this case (and in some other instances) the thread- or whip-like body form could be attributable to simple genetics but may also (and equally) be a response to specific environmental or microhabitat conditions, demonstrating again the plasticity of these organismal multi-unit colonies.

Two distinct audiences might find helpful the work related in this review; those researchers whose primary interests are the gorgonian coral species of the California Bight or the eastern Pacific Ocean (and indeed, forms of gorgonian, in general) and those field biologists, ecologists and taxonomists who encounter gorgonian corals in the context of survey and study of other marine phyla. The work presented in this three part-work does not completely resolve all outstanding taxonomic questions regarding eastern Pacific species. There are several taxon groups that still need extensive work from a taxonomic perspective. As well, new species are likely to become more common or evident in collections. It may be that many new species are already housed in those collections, but have not been looked at, or, because of insufficient material to make comparisons with, have been looked at, then placed back into a drawer as no satisfying conclusions could be drawn.

### Conclusions

While the gorgonian material at SBMNH encompasses a good working collection, reflecting to a significant degree the diversity of these animals as seen within the California Bight, it is apparent that further material is needed to enhance and complete the research collection. As access to deep-water sites, not only in the California Bight, but throughout the United States’ coastal eastern Pacific region improves and becomes a more common occurrence (hopefully), the discovery of new forms of gorgonian are certainly a possibility. However, the SBMNH research collection, and this study review, has already revealed several key things: 1) a higher diversity of both genera (those previously reported and several not previously reported) and species (encompassing those previously reported, those already known but newly reported for the CA Bight, as well as entirely new species) occurs in the region than was initially thought, 2) that a greater degree of understanding is necessary to adequately know the genus *Swiftia* and those morphologically similar “Red whip” forms, 3) that some interesting and significant geographical/ecological trends (transitional endemics, etc.) exist within certain genera along the California coast, and 4) that morphological plasticity is clearly displayed, likely reflecting both genetic makeup and response to several dynamic environmental conditions.

The collection highlights several taxonomic groups still in need of further study (“Red whips,” the genera *Thesea* and *Muricea*) and those groups/genera where further collected material would be invaluable (*Paragorgia*, *Sibogagorgia*, *Placogorgia*, *Acanthogorgia*, *Swiftia*, Primnoidae, to name but a few). And most significantly, were it not for some of those early expedition pioneers working in the eastern Pacific, notably the Allan Hancock Foundation’s ‘Velero’ Expeditions of 1931–1941 and 1948–1985, we would not have nearly as much material to work with as we do. The SBMNH collection will, with effort, continue to evolve, becoming an ever more valuable research tool as the work continues. As the SBMNH is the sole repository for the bulk of the AHF cnidarian collection, and one of the few museum collections in California (indeed, throughout all the western coastal United States) that has been fully curated and extensively reviewed and studied, focusing on gorgonians of the California Bight region, the SBMNH research collection is a significant resource for those studying this cnidarian group.

## Supplementary Material

XML Treatment for Primnoidae

XML Treatment for Swiftia kofoidi

XML Treatment for Swiftia pacifica

XML Treatment for Swiftia simplex

XML Treatment for Swiftia cf. spauldingi

XML Treatment for Swiftia torreyi

XML Treatment for Swiftia pusilla

XML Treatment for Thesea

XML Treatment for Thesea

XML Treatment for Primnoidae

XML Treatment for Callogorgia

XML Treatment for Callogorgia kinoshitai

XML Treatment for Parastenella

XML Treatment for Parastenella pacifica

XML Treatment for Parastenella ramosa

XML Treatment for Plumarella

XML Treatment for Plumarella longispina

XML Treatment for Primnoa

XML Treatment for Primnoa pacifica

XML Treatment for Narella

XML Treatment for Isididae

XML Treatment for Acanella

XML Treatment for Isidella

XML Treatment for Keratoisis

XML Treatment for Lepidisis

## Appendix 1

**Table A1.** Contrasts and comparisons of key “red whip” species and/or species of the genus *Swiftia*, as represented in SBMNH collection.

Red Whip species	Location, S to N	Location Depth	Colony Branching	Colony Color	Polyp Spacing	Polyp Height	Sclerite Color	Sclerite Form	Sclerite Size
* Leptogorgia flexilis *	Magdalena Bay, Baja to San Diego, CA	11 meters to ?	Thin, drooping branches; highly branched colony	Red/pink to tan/beige Polyps white to very pale orange	No more than 1 mm	No more than 1 mm	Bright Salmon	Spindles & Capstans	0.03–0.09 mm
* Leptogorgia chilensis *	N of Magdalena Bay to Santa Cruz Is., CA	Approx. 15–80 m	Thin branches; moderately branched	Orange-red Polyps white	1 mm	Generally, almost flush	Bright Salmon	Spindles & Capstans	0.03–0.05 mm
Red Whip (?”Transitional/Regional Endemic”)	San Diego, CA to off Oregon coast	?20-2,000 meters	Moderate thickness to branches; slightly branched to not branched	Orange-red Polyps white	Varies from 1 to 2 mm	Generally, from flush to nearly 1 mm; rarely taller	Salmon	Spindles & Dbl. Spinds.	Approx. 0.1 mm
Red Whip (?”Transitional/Regional Endemic”)	San Diego, CA to off Oregon coast; possible extension to WA coast	Approx. 12–150 m	Moderately thin branches, whip-like; slightly branched	Orange-red Polyps white (?pale pink)	Varies from 1 to 2 mm	Generally, consistently flush, rarely taller; on some, prominent	Salmon	Spindles & Dbl. Spinds.	Approx. 0.1 mm
“?*Swiftia* Transitional/ Regional species”	N Los Angeles County to Point Conception	Approx. 104–173 m	Single branches; also slightly branched (if so, dichotomous)	Bright red to salmon-pink Polyps white	Less than 1 mm	Approx. 1 mm	Salmon	Spindles; very few Capstans, Dbl. Spinds. or Rods	From 0.04 mm to nearly +.16 mm
* Swiftia simplex *	N Los Angeles County to Alaska	200–900 m	Single branches; sometimes slightly branched	Pinkish-red (Brick-red) Polyps pinkish-red	No more than 2 mm	Approx. 1 mm	Pinkish-red (Brick color) Rods orange	Spindles, Capstans, Rods and Dbl. Spinds.	0.1–0.3 mm
* Chromoplexaura marki *	Point Conception to Cape Mendocino, CA (?further north to WA state, on to Alaska)	20–60 m; possibly deeper (to 600 m)	Single branches; sometimes slightly to moderately branched	Bright red, orange, even pinkish Polyps white or colored	2 mm	Nearly flush to 2 mm	Salmon to reddish	Spindles, Capstans, Ovals and Dbl. Spinds; NO Rods	0.05 mm to ≅ 0.2 mm
* Swifita spauldingi *	Monterey Bay, CA to off Washington coast (?further north to Alaska)	40 to at least 300 m	Moderate branch thickness; branched to some degree	Orange-red Polyps white or very pale pink	about 1 mm	Nearly flush to often very conspicuous, rounded	Salmon to pinkish- orange; some yellow; Rods orange	Spindles, Capstans, Rods and Dbl. Spinds.	About 0.1 mm

Polyp Height: includes both calyx and actual polyp.

## Appendix 3

**List of material examined – Part III**

(Material examined = Whole colony study plus multiple sclerite preparations; all with light microscopy, plus selected colonies under SEM, shown in figures associated with text)

**Swiftiacf.kofoidi (Nutting, 1909)**

**Material examined.** ~20–25 lots. **USA, California** – 2 colonies; Los Angeles County, 5.5 or 6 miles off SE end, or SE of, Santa Catalina Island, 33°15'00"N, 118°11'35"W (end), gravel, rock, 264–282m; coll. R/V ‘Velero III’, station 1188-40, 29 September 1941; SBMNH 422955 [wet]. –1 colony; Los Angeles County, 6.25 miles NE or ENE X E of Long Point, Santa Catalina Island, 33°25'20"N, 118°14'40"W (end), rocks, sponges, cyclostomes, 415–486 m; coll. R/V ‘Velero III’, station 1400-41, 8 September 1941; SBMNH 422956 [wet]. –several fragments; Los Angeles County, San Pedro Channel, 70 Fathom Bank, on rock and pebbles, 33°24'15"N, 118°00'35"W (end), 238 m; coll. R/V ‘Velero III’, station 1213-40, 30 Nov. 1940; SBMNH 422957 [wet]. –fragment; Los Angeles County, off Redondo Beach, 33°49'55"N, 118°25'45"W (end), on gray mud and shell, 175–218 m; coll. R/V ‘Velero III’, station 1137-40, 5 May 1940; SBMNH 422958 [wet]. –1 colony; 6.7 miles, 330° T from N Light, Santa Barbara Island, dredge, tangles–2 large boulders and much small rock, rock bottom, 33°33'27"N, 119°04'00"W (end), 255 m; coll. R/V ‘Velero IV’, station 2062-51, 18 October 1951; SBMNH 422959 [wet]. –fragment; Santa Barbara County, Santa Rosa Island, 16.5 miles SSE of East Point, 33°40'55"N, 119°52'30"W (end), rocks, crinoids, sponges, 136–138 m; coll. R/V ‘Velero III’, station 1385-41, 25 August 1941; SBMNH 422960 [wet]. –1 fragment; Santa Barbara County, 10 miles SE X 1/2E of South Point, Santa Rosa Island, 33°46'30"N, 119°58'30"W (end), mud, rock and gravel, 195–227 m; coll. R/V ‘Velero III’, station 1393-41, 26 August 1941; SBMNH 422961 [wet]. –multiple fragments (one lot); Santa Barbara County, Santa Rosa Island, 7.37 miles, 350° T to East Point, 33°48'40"N, 119°56'20"W (end), 116 m; coll. R/V ‘Velero IV’, station 23291-75, 13 November 1975; SBMNH 422964 (with label reading: 23291 CH) [wet]. –2 colonies, fragmented (no base); Santa Barbara County, Santa Rosa Island, 2.59 miles, 291.5° T to Ford Point, 33°54'00"N, 120°00'00"W, 40 m; coll. R/V ‘Velero IV’, station 23290-75, 13 November 1975; SBMNH 422962 (with label reading: 23290 CH) [wet]. –2 colonies, on deep-water bivalve; Santa Barbara Channel, 34°15'00"N, 120°00'00"W, ~136 m; coll. Peterson, BLM, by dredge, June 1964; SBMNH 422967 [wet]. –2 colonies, fragmented (no base); Santa Barbara County, Santa Rosa Island, 6.1 miles, 50° T to Sandy Point, 33°56'00"N, 120°20'00"W (end), 127 m; coll. R/V ‘Velero IV’, station 24879-76, 28 April 1976; SBMNH 422963 (with label reading: 24879 CH) [wet]. **MEXICO, Baja California Norte (Pacific Coast)** – 1 colony; west coast side of Isla Cedros, 28°13'02"N, 115°15'01"W; coll. Pacific BioMarine, 26 April, 1974; SBMNH 422965 [DH ? = SBMNH-41, dry].

**Other material examined.** –1/few fragments; USA, California, Santa Barbara County, off Point Conception, 34°26'23"N, 120°28'31"W, SWFC station 5, 727 m; coll. R Snodgrass, 6 March 1986; SIO/BIC CO2024 [wet]. –1 colony; USA, California, Monterey County, Monterey Bay, 36°26'39"N, 122°01'47"W, ~684 m; coll. G McDonald, 14 August 1974; MLML C0072 [wet]. –1 colony; USA, California, Monterey County, Monterey Bay, ~36°46'15"N, 121°53'27"W, 504 m; coll. G McDonald, 29 September 1973; MLML C0060 [wet]. –1 colony; USA, California, Monterey County, Ascension Canyon, 36°55'05"N, 122°27'30"W, attached to sea anemones, ~1,245 m; coll. unknown, 4 June 1984 MLML C0140 [wet]. –colony; USA, California, Monterey County(?); coll. MBARI, T138-A1, NOAA CB 33994 [wet].

No locality could be ascribed to an additional colony as the station number did not correspond with the year; coll. unknown; ‘Albatross’, station (4- or) 5054, 1904; SIO/BIC CO360 [wet].

***Swiftiapacifica* (Nutting, 1912)**

**Material examined**: ~23 lots. **USA, California** – 1 colony + 1 fragment (2 lots); Ventura County, ~6 miles due south of Anacapa Island, Pilgrim Banks (Piggy Bank), at intersection of Santa Cruz Canyon and Pilgrim Banks, between Anacapa and Santa Cruz Islands, Channel Islands National Marine Sanctuary, 33°55'15"N, 119°28'18"W, 280–320 m; coll. NOAA vessel, ‘McArthur II’, Leg 3, 27 June-02 July 2010; NOAA ID Nos.: K2_01_062710_03 and K2_01_062710_10; SBMNH 232035/232036 [wet].

**Other material examined. USA, California** – 1 colony; Humboldt County, off Cape Mendocino, on the edge of Gorda Escarpment, 40°18'42"N, 124°59'06"W, 1,063 m; coll. NOAA, WCGS, 2007; CB 34406-040, FRAM/Cutting Barcode 112080 [wet]. **USA, Oregon** – 1 colony; Coos County, off Oregon coast, S and W of Bandon, ~43°01'37"N, 124°48'36"W, ~218 m; coll. NOAA, 2006; CB 34213-063, FRAM/Cutting Barcode 100105485 [wet]. –1 colony; Lane County, off Oregon coast, on Heceta Bank, near southern edge of Heceta Escarpment, due W of Florence, ~43°56'42"N, 124°55'06"W, ~397 m; coll. NOAA, 2006; CB 34213-054, FRAM/Cutting Barcode 100105476 [wet]. –1 colony; off Oregon coast, Heceta Bank, 43°57'10"N, 124°50'38"W, hard bottom, 134.8 m; coll. A Valdés by ROV; RV ‘Ronald H. Brown’ (NOAA), and S/V ‘Ropos’, Dive 615, 10 July 2001; LACoMNH Marine Biodiversity processing center number 99 [wet]. –2 colonies; off Oregon coast, Heceta Bank, 44°04'04"N, 124°55'11"W, muddy bottom, collected with small rock, sponge, 159.7 m; coll. A Valdés by ROV; RV ‘Ronald H. Brown’ (NOAA) and S/V ‘Ropos’, Dive 606, 6 July 2001; LACoMNH Marine Biodiversity processing center number 36 [wet]. –1 colony; Lincoln County, off coast, north of Hydrate Ridge, 44°46'01"N, 125°03'27"W, 1,159 m; coll. NOAA, RACE, CB 50003-008, 1996 [wet]. –1 colony; off Oregon coast, 46°06'22"N, 124°55'03"W, 1123.5 m; coll. Astoria RB-01-05, G Hendler; RV ‘Ronald H. Brown’ (NOAA) and S/V ‘Ropos’, Dive R602-Bio-0007, 3 July 2001; LACoMNH Marine Biodiversity Center processing number 373 [wet]. **USA, Washington** –multiple colonies; Grays Harbor County, Quinault Canyon, 47°32'05"N, 125°11'05"W, 558 m; coll. R/V ‘John N. Cobb’, 16 March 1962, USNM 53971 (labeled as *S.torreyi*) [wet]. –multiple colonies/3 lots; Jefferson County, W of Queets, 47°35'13"N, 125°09'17"W, 549 m; coll. R/V ‘John N. Cobb’, 16 March 1962; USNM 57219, 57220 and 57221 [wet]. –1 colony; Juan de Fuca Canyon, 47°55'06"N, 125°29'02"W, 429 m; coll. OCNMS Survey Expedition, 2008; EPI 202 [wet]. –fragments; Clallam County(?), no specific location or collection data provided; coll. OCNMS Survey Expedition, 2008; Jar 807, EPI 230 [wet]. –fragments; Clallam County, E edge of Swiftsure Bank, ~21 miles W of Ozette, 48°08'31"N, 125°11'10"W, 267 m; coll. OCNMS Survey Expedition, 2008, 12 July 2008; Jar 795, EPI/SUC 216 [wet]. –several colony fragments; Clallam County, off coast, just south of Cape Flattery, 48°10'05"N, 125°07'01"W, 230 m; coll. P Etnoyer, NOAA West Coast Survey, Fall 2010, 5 November 2010; BS007 [wet]. –1 colony; Clallam County, approx. 9–12 miles SW of Cape Flattery, Olympic Coast National Marine Sanctuary, 48°18'11"N, 124°56'10"W, 265.7 m; coll. OCNMS Survey Expedition, 2006, 1 June 2006; Jar 713, OC06-003, EPI 127 [wet]. –1 colony; Clallam County, approx. 9–12 miles SW of Cape Flattery, Olympic Coast National Marine Sanctuary, 48°18'13"N, 124°56'10"W, 250.4 m; coll. OCNMS Survey Expedition, 2006, 1 June 2006; Jar 714 [wet]. –colonies in 3 lots; Clallam County, approx. 9–12 miles SW of Cape Flattery, Olympic Coast National Marine Sanctuary, 48°18'17"N, 124°56'08"W, 251.5 m; coll. OCNMS Survey Expedition, 2006, 1 June 2006; Jars 381-A, B and C [wet]. –1 fragment; Clallam County, E edge of Swiftsure Bank, just W of entrance to Strait of Juan de Fuca, ~10 miles from Cape Flattery, Olympic Coast National Marine Sanctuary, 48°24'33"N, 124°57'45"W, 273 m; coll. OCNMS Survey Expedition, 2008, 9–13 July 2008; Jar 733, EPI 153 [wet]. **USA, Alaska** –1 colony; Gulf of Alaska, no lat/long, no depth, no date; Alaska Fisheries Science Center, NOAA Fisheries, 41-39-1 [dry]. –1 colony; Bering Sea, Aleutian Islands, near islands, SE of Agattu Island, 52°13'55"N, 174°13'24"W, 881 m; coll. R/V ‘Albatross’, station 4781, 7 June 1906; USNM 30024 (labeled as *Callistephanuspacificus*) [wet]. –colonies; Gulf of Alaska, Denson Seamount, 53°56'58"N, 137°23'51"W, 2,432 m; coll. Amy Baco-Taylor using ‘Alvin’ DSR/V, 3 August 2004; USNM 1075771 [dry]. –1 colony; 26 miles south of Chowiet Island, 55°36'12"N, 157°05'39"W, 86 m; coll. NOAA, RACE, 2000; CB 50003-021 [wet]. –1 colony; Gulf of Alaska, ~57°37'29"N, 136°37'09"W, 803 m; coll. unknown, 19 July 2004; Alaska Fisheries Science Center, NOAA Fisheries, 41-100A-2 [dry]. –colonies; Gulf of Alaska, Kayak Island, 59°29'06"N, 144°50'42"W, 601–800 m; coll. unknown, 31 July 2002; USNM 1011170 [dry].

**Other material, not examined.** – colony (?); Hawaii (likely an error in location); coll. R/V ‘Albatross’, 1902; part of a specimen from Bishop Museum collection, #101–as **Holotype**; USNM 49513 (labeled as *Allogorgiaexserta*) [wet].

Additional specimens collected from the Bering Sea, Pribilof Islands and British Columbia (Queen Charlotte Islands) found in NMNH collection.

***Swiftiasimplex* (Nutting, 1909)**

**Material examined.** ~24 lots. **USA, California** – 1 colony; North Pacific, Monterey County, Monterey Bay, 5.4 miles (5.8 miles), 313°T (314°T) from Point Piños Light to Mid Point, 36°42'05"N, 122°01'45"W (end), 436–809 m; coll. R/V ‘Velero IV’, station 7462-61 or 7463-61, 10 October 1961; SBMNH 422979 [wet].

**Other material examined. USA, California** – Eastern N Pacific, San Juan Seamount, 33°05'46"N, 120°56'57"W, 746.2 m; coll. MBARI staff, PI, D Clague, 2 May 2004; MBARI T665-A5, 2004-123 [wet]. –1 colony; Los Angeles County, Channel Islands, Santa Barbara Island, bearing N 49°, W 4.7 miles, 815 m; coll. USBCF ‘Albatross’, station 4416, SW, Rock, 1904; potential **Paratype** (?); SIO/BIC CO990 [wet]. –Eastern N Pacific, Santa Barbara County, Channel Islands, Santa Cruz Island, Point San Pedro, ~34°02'03"N, 119°31'12"W, 817–933 m; coll. R/V ‘Albatross’, 14 April 1904; **Syntype**, USNM 25431, (Nutting) [dry]. –Eastern N Pacific, Santa Barbara County, Channel Islands, Santa Cruz Island, Point San Pedro, ~34°02'03"N, 119°31'12"W, 817–933 m; coll. R/V ‘Albatross’, 14 April 1904; **Syntype**, USNM 43130, (Nutting) [dry]. –Eastern N Pacific, West of San Miguel Passage, Rodriquez Seamount, 34°01'12"N, 121°04'48"W, 895.3 m; coll. MBARI staff, PI, D Clague, 15 October 2003; MBARI T630-A13 [wet]. –Eastern N Pacific, West of San Miguel Passage, Rodriquez Seamount, 34°03'00"N, 121°03'00"W, 637.6 m, on an Arkosic sandstone erratic; coll. MBARI staff, PI, D Clague, 13 October 2003; MBARI T628-R22-1 [wet]. –Eastern N Pacific, West of San Miguel Passage, Rodriquez Seamount, 34°03'00"N, 121°03'00"W, 689 m; coll. MBARI staff, PI, D Clague, 13 October 2003; MBARI T628-A6 [wet]. –Eastern N Pacific, NE of Davidson Seamount, just S of Monterey Bay, ~36°44'24"N, 122°02'24"W, 870–911 m; coll. MBARI staff, PI, D Clague, 24 June 2007; MBARI T1104-A6 [wet]. –Monterey County, Monterey Bay, in channel, 732–914 m; coll. unknown, 25 June 1925; USNM 77287 [dry]. –Monterey County, Monterey Bay, ~36°27'41"N, 122°06'37"W, 504 m; coll. G McDonald, 29 September 1973; MLML C0051 [wet]. –San Mateo County, off Pigeon Point, ~37°10'55"N, 122°23'40"W, 273 m; coll. M/V ‘Joseph Alioto’, no collection date; CAS-IZ 96744 [wet] (as *Euplexaurasimplex*). –Eastern N Pacific, Carmel Canyon, 198 m; coll. M Morris, 12 April 1976; MLML C0038 [wet]. –Eastern N Pacific, Pioneer Seamount, ~37°24'03"N, 123°32'24"W, 847.5 m; coll. MBARI staff, PI, D Clague, 19 June 2007; MBARI T1101-A20 [wet]. –Marin County, W of Point Reyes, Cordell Bank National Marine Sanctuary, southwest of Cordell Bank, ~37°56'34"N, 123°28'19"W, 182–396 m; coll. N.B. ‘Scofield’, 25 August 1949; CAS-IZ 96739 [wet] (as *Euplexaurasimplex*). –1 colony; Mendocino County, off the coast, 39°12'28"N, 124°10'30"W, 991 m; coll. NOAA, WCGS, 2006; CB 34212-014 [wet]. –Humboldt County, Eel River Canyon, ~40°38'01"N, 124°21'26"W, 364 m; coll. RR Talmadge on R/V ‘Flicker’, December 1966; CAS-IZ 96758 [wet] (as *Euplexaurasimplex*). **USA, Oregon** – 2 colonies; off Oregon coast, Heceta Bank, 43°56'35"N, 124°57'11"W, 340 m; coll. Heceta RB-00-05, G Hendler; RV ‘Ronald H. Brown’ (NOAA) and S/V ‘Ropos’, Dive R534-bio-0001, 23 June 2000; LACoMNH Marine Biodiversity Center processing number 227 [wet]. –1 colony; off Oregon coast, Heceta Bank, 44°11'33"N, 124°58'15"W, cold seep, 275 m; coll. N Puniwai by ROV; RV ‘Ronald H. Brown’ (NOAA) and S/V ‘Ropos’, Dive 609, 8 July 2001; LACoMNH, Marine Biodiversity Center processing number 68 [wet]. –1 colony; Lincoln County, off the coast, north of Hydrate Ridge, 44°46'01"N, 125°03'27"W, 1,159 m; coll. NOAA, RACE, 1996; CB 50003-009 [wet]. **USA, Washington** –2 colonies; Grays Harbor County, 11 miles south of Quinault Canyon, 47°13'49"N, 125°07'35"W, 838 m; coll. NOAA, WCGS, 2007; CB 34405-028, FRAM/Cutting Barcode 105535 and FRAM/Cutting Barcode 127018. –1 colony; Jefferson County, between Juan de Fuca Canyon and Quinault Canyon, 47°38'09"N, 125°33'45"W, 1,184 m; coll. NOAA, WCGS, 2006; CB 34212-039, FRAM/Cutting Barcode 100081368 [wet]. **USA, Alaska** –North Pacific, Gulf of Alaska, 56°06'54"N, 144°15'45"W, 546–664 m; coll. unknown, 2 July 2000; USNM 1006328 [dry]. –1 colony; Gulf of Alaska, ~57°54'15"N, 137°33'20"W, 601 m; coll. unknown, 21 July 2008; Alaska Fisheries Science Center, NOAA Fisheries, 81-99B-1 [dry].

Unknown location: WCGS 2006–2006H30 (No FRAM or CB number), provided by E Berntson, NOAA Fisheries Office, Port Orchard, WA.

**Swiftiacf.spauldingi (Nutting, 1909)**

**Material examined.** ~9 lots

**Other material examined.** – Eastern North Pacific, USA, California, Monterey County, Monterey Bay, in channel, 128 m; 13 April 1928; USNM 78385 [dry]. –Eastern North Pacific, USA, California, Monterey County, Monterey Bay; **Holotype**, USNM 91854 [wet]. –Eastern North Pacific, USA, California, Monterey County, Monterey Bay, 146 m; 22 January 1930; USNM 77289 [dry]. –Eastern North Pacific, USA, California, Monterey Bay, 91 m; 8 February 1930; USNM 77288 [dry]. –1 colony; Eastern North Pacific, Oregon, Curry County, 21 miles slightly north and west of Gold Beach, 42°27'26"N, 124°50'24"W, 452 m; coll. NOAA/NFSC, WCGS, 2008; CB 34806-455, FRAM/Cutting Barcode 100116455 [wet]. –Eastern North Pacific, Washington State, W of Juan de Fuca Strait, 48°30'00"N, 124°57'00"W, 49 m.; 24 September 1888; USNM 75052 [dry].

Several other lots [wet], indicating collection locations off the coast of USA, Oregon, Lane County, at Heceta Bank [USNM 57165, wet], and in the Strait of Juan de Fuca, USA, Washington [USNM 57158, wet].

***Swiftiatorreyi* (Nutting, 1909)**

**Material examined**. ~16 lots.

**Other material examined. USA, California** – San Diego County, San Diego, Point Loma, 31°59'53"N, 116°59'59"W, 201–262 m; coll. R/V ‘Albatross’, station 4311, 4 March 1904; USNM 49522 [wet]. –Eastern N Pacific, Rodriquez Seamount, west of San Miguel Passage, 34°02'57"N, 121°06'03"W, 1,029 m; coll. MBARI staff, PI, D Clague, 15 October 2003; MBARI T630-A7 [wet]. –Eastern N Pacific, Rodriquez Seamount, west of San Miguel Passage, 34°02'57"N, 121°06'03"W, 1,029 m; coll. MBARI staff, PI, D Clague, sample T630-A7, 15 October 2003; USNM 1027078 [wet]. –Eastern N Pacific, Davidson Seamount, ~35°41'51"N, 122°41'58"W; coll. MBARI staff, PI, A DeVogeleare, 22 May 2002; MBARI T429-A20C [wet]. –Monterey County, Monterey Bay, 36°38'00"N, 121°55'00"W (bearing S 78°E, 6.8 miles) off Point Piños light-house, 1,373–1,742 m; coll. USBCF ‘Albatross’, station 4530, 27 May 1904; **Holotype**–USNM 25433 [wet]. –Monterey County, Monterey Bay, 36°38'00"N, 121°55'00"W (bearing S 78°E, 6.8 miles) off Point Piños light-house, 1,373–1,742 m]; coll. USBCF ‘Albatross’, station 4530, 27 May 1904; **Holotype**–USNM 43147 [wet]. –Monterey County, Monterey Bay, 36°38'00"N, 121°55'00"W (bearing S 74°E, 7.4 miles) off Point Piños light-house, 1,574.77–1,942.4 m; coll. R/V ‘Albatross’, station 4537, 31 May 1904, det. Nutting; **Paratype**–USNM 58985 [dry]; previous lat./long., 1,566–1,931 m; CAS-IZ 96786 [wet]. –Monterey County, Monterey Bay, 146 m; coll. unknown, 20 February 1930; USNM 87968 [dry]. –Monterey County, Monterey Bay, Point Piños, 36°38'00"N, 121°55'00"W, 1,381–1,752 m; coll. R/V ‘Albatross’, station 4530, 27 May 1904; USNM 91878 [wet]. –Monterey County, Monterey Bay, 36°38'00"N, 121°55'00"W (bearing S 46°E, 8.4 miles) off point Piños light-house, 1,552.82 m; coll. R/V ‘Albatross’, station 4546, 3 June 1904, det. Nutting; **Paratype**–USNM 91896 [dry]. –Monterey County, Monterey Canyon, Point Sur, by beam trawl; coll. MLML staff on class cruise, PI, K Coale, 7 April 2008; no station or collection number [wet]. –Eastern N Pacific, Pioneer Seamount, ~37°23'49"N, 123°23'58"W, depth unknown; coll. MBARI staff, PI, D Clague, 18 June 2007; MBARI T1100-A7, 61807 [wet]. –Eastern N Pacific, Pioneer Seamount, ~37°23'49"N, 123°23'58"W, 847.5 m; coll. MBARI staff, PI, D Clague, 19 June 2007; MBARI T1101-A21, 61907 [wet]. **USA, Washington** – Grays Harbor County, Quinault Canyon, 47°28'59"N, 125°11'45"W, 558 m; coll. R/V ‘John N Cobb’, 16 March 1962; USNM 53971 [wet] (? on ID). –1 colony; Clallam County, edge of Swiftsure Bank, W of Olympic Peninsula, ~47°55'54"N, 125°29'11"W, 429 m; coll. OCNMS Survey Expedition 2008, 12 July 2008; Jar 787, EPI 202 [wet]. –Olympic Peninsula, Clallam County, 48°07'22"N, 125°50'39"W, depth not indicated; coll. R/V ‘Miller Freeman’, 2000 west coast slope fisheries survey, 12 October 2000; USNM 100840 [wet]. –Strait of Juan de Fuca, ~2,200 m; coll. MBARI staff, PI, D Stakes, 4 September 2004; MBARI T738-A1, 2004-248 [wet].

**Other material examined.** – USA, California, Monterey County, Monterey Bay, 36°38'00"N, 121°55'00"W (bearing S 39°E, 10.7 miles) off Point Piños light-house, 716–953 m; coll. USBCF ‘Albatross’, station 4514; repository unknown. –1 colony; no location data or date; CAS-IZ 96794 [wet].

***Swiftiapusilla* (Nutting, 1909)**

**Material examined.** No specimens in SBMNH collection.

**Other material examined.** – fragment; USA, California, San Diego County, San Diego, Point Loma, ~32°39'10"N, 117°17'47"W, 166–177 m; coll. R/V ‘Albatross’, station 4361, 15 March 1904; USNM 25430, **Holotype** [wet & dry]. (Identification still uncertain.)

***Thesea* sp. (various possible species, such as *T.filiformis, T.mitsukurii* ?)**

**Material examined.** ~65 lots. **USA, California** – 1 fragment; San Diego County, off Point Loma, mud, ~32°38'30"N, 117°13'20"W, 64–69 m; coll. ‘EW Scripps’, 9 October 1946; (in ovulid snail collection, with shells of *Neosimnialoebbeckeana*), SBMNH 13304 [dry]. –multiple fragments; San Diego County, off Point Loma, 32°39'00"N, 117°14'00"W, with an ID H46-102; SBMNH 422908 [wet]. –2 fragments; San Diego County, San Diego, from a station off Point Loma, ~32°41'03"N, 117°16'35"W; coll. MBL, no other data; SBMNH 265944 [wet]. –fragments; San Diego County, San Diego, from a station off point Loma, station SD-1 ~32°41'03"N, 117°16'35"W, 61 m; coll. MBL, 12 January 1998; SBMNH 265943 [wet]. –fragments; San Diego County, 2.1 miles, 268° T from Scripps Institute Pier, Hayward grab–compact green mud, 32°52'00"N, 117°18'00"W, coll. R/V ‘Velero IV’, station 4757-56, 8 December 1956; SBMNH 422346 [wet]. –fragments; Los Angeles County, San Clemente Island, 1 mile NE of Castle Rock, gray sand, 33°03'00"N, 118°36'20"W (end), 84–91 m; coll. R/V ‘Velero III’, station 1326-41, 8 June 1941; SBMNH 422339 [wet]. –strands; Los Angeles County, Santa Catalina Island, 1 mile SW of Ben Weston Point, mud, sand, gravel, 33°20'55"N, 118°30'35"W (end), 82–89 m; coll. R/V ‘Velero III’, station 1316-41, 17 May 1941; SBMNH 265942 [wet]. –fragments; Los Angeles County, off Corona del Mar, 33°35'48"N, 117°52'48"W, 46 m; coll. MacGinitie, 21 February 1954; SBMNH 422355 [wet]. –multiple fragments; Los Angeles County, Catalina Island, Ship Rock, 33°27'00"N, 118°29'00"W, 82–101 m; coll. J Morin, 7 July 1977; SBMNH 422353 [wet]. –strands; Los Angeles County, Catalina Island, 2 mi. W of Church Rock, mud, sand, 33°17'25"N, 118°21'50"W (end), 82–96 m; coll. R/V ‘Velero III’, station 1321-41, 18 May 1941; SBMNH 422338 [wet]. –multiple strands; Los Angeles County, Santa Catalina Island, White Cove, mud and sand, 33°23'05"N, 118°21'00"W, 66–75 m; coll. R/V ‘Velero III’, station 998-39, 12 August 1939; SBMNH 422335 [wet]. –a few strands; Los Angeles County, Santa Catalina Island, off Bird Rock, rock, coarse shell, kelp, 33°27'20"N, 118°29'00"W (end), 56–73 m; coll. R/V ‘Velero III’, station 1187-40, by small dredge boat, 29 Sept. 1940; SBMNH 422347 [wet]. –fragments; Los Angeles County, N of Santa Catalina Island, off Eagle Bank, gray sand, 33°27'40"N, 118°30'00"W (end), 73–78 m; coll. R/V ‘Velero III’, station 1178-40, 10 September 1940; SBMNH 422345 [wet]. –multiple fragments; Los Angeles County, 3.6 miles, 250° T from Newport Beach Pier, Hayward grab–dark green, sandy silt, 33°35'12"N, 117°59'52"W; coll. R/V ‘Velero IV’, station 5087-57, 22 May 1957; SBMNH 422337 [wet]. –multiple colony fragments; Los Angeles County, 8.6 miles, 142° T, from Point Fermin, dredge–sand, 33°35'34"N, 118°11'03"W (end), 49 m; coll. R/V ‘Velero IV’, station 2043-51, 20 July 1951; SBMNH 422332 [wet]. –multiple strands/colonies; Los Angeles County, Palos Verdes Estates, Bluff Cove, 33°47'18"N, 118°24'47"W; coll. T Burch, station 40129, 22 August 1940; SBMNH 423086 [wet]. –multiple strands; Los Angeles County, off Redondo Beach, 33°50'48"N, 118°24'51"W, 27–91 m; coll. T Burch, station 3929, 3 August 1939; SBMNH 265941 [wet]. –multiple fragments; Santa Monica, Santa Monica Bay, 33°51'35"N, 118°26'49"W, by VanVeen grab, 60 m; coll. City of Los Angeles, Environmental Monitoring Division, 21 July 2003; LACoMNHMarine Biodiversity Center process number 10231 [wet]. –3 samples (combined), multiple strands; Los Angeles County, near Hyperion Stack, between Castle Rock and the Manhattan Beach Pier, lot 4068–.85 miles, 285° T from Hyperion Stack, 33°55'58"N, 118°26'52"W, lot 4069.9 miles, 294° T from Hyperion Stack, 33°56'07"N, 118°26'51"W, lot 4071–1.4 miles, 235° T from Hyperion Stack, 33°54'57"N, 118°27'15"W; coll. R/V ‘Velero IV’, stations 4068, 4069 & 4071, 16 April 1956; SBMNH 422907, [wet]. –multiple colonies/fragments; Los Angeles County, Santa Monica Bay, dredge–rocky bottom, 33°52'16"N, 118°31'44"W (end), 67 m; coll. R/V ‘Velero IV’, station 3539-55, 12 October 1955; SBMNH 422344 [wet]. –numerous fragments (colonies); Los Angeles County, Santa Monica, 34°00'02"N, 118°30'41"W, 61 m; coll. SCCWRP, station S-20, 16 April 1974; SBMNH 422354 [wet]. –fragments/colonies; Santa Barbara County, 3 miles south of Santa Barbara, 34°22'23"N, 119°40'21"W, 76 m; coll. P Scott, 29 September 1986; SBMNH 422352 [wet]. –numerous fragments/colonies (mixed species?); Santa Barbara County, 3 miles south of Santa Barbara, 34°22'23"N, 119°40'21"W, 76 m; coll. P Scott, 29 Sept. 1986; with barnacles, possibly from the genus *Lepas* attached; SBMNH 422356 [wet]. –several fragments (long); Santa Barbara County, Santa Barbara, 2 miles off Lighthouse, 34°22'01"N, 119°43'12"W, 55 m; coll. J Vucci and J Butterfield, 29 April 1974, with an otter trawl on a sand and rock bottom; SBMNH 422905 [DH 416 =SBMNH-01; dry]. –fragment/colony; Santa Barbara County, N of Santa Barbara, 5 Mile Reef, 34°45'00"N, 123°02'00"W, 204 m; coll. P Brophy, 4 June 1967; SBMNH 45694 [wet]. –1 fragment; Santa Barbara County, N of Santa Barbara, 5 Mile Reef, 34°45'00"N, 123°02'00"W, 204 m; coll. P Brophy, 4 June 1967; [DH, SBMNH-05; SBMNH 422906; dry]. –several colonies; California Channel Islands, by trawl, 55 m; coll. P Brophy, April 1974; SBMNH 45596 [wet]. –1 colony, with base (?); California Channel Island area, by trawl; coll. P Brophy; SBMNH 45606 [wet]. –fragment/colony; Santa Barbara County, Santa Cruz Basin, in a beam trawl, 10.5 miles, 242° S from Anacapa Lighthouse, 33°55'00"N, 119°35'45"W, 909 m; coll. T Phillips, ‘Velero IV’, station 13621-69, 14 November 1969; SBMNH 45598 [wet]. –1 colony fragment; Santa Barbara County, 3.7 miles, 21° T to Crook Point, San Miguel Island, 33°56'48"N, 120°21'42"W (end), 118 m; coll. R/V ‘Velero IV’, station 24882-76, 28 April 1976; SBMNH 422904 [wet]. **MEXICO, Baja California Sur (Pacific Coast)** – multiple fragments; 7 miles, 260° T from Punta Abreojos, sigsbee trawl, sand and mud bottom, 26°39'48"N, 113°40'43"W (end), 44 m; coll. R/V ‘Velero IV’, station 1953-50, 20 April 1950; SBMNH 422342 [wet]. –1 colony; 8.5 miles S of Canal de Dewey, sand, broken shell, gravel, 27°42'15"N, 115°05'02"W (end), 89 m; coll. R/V ‘Velero III’, station 1259-41, 27 February 1941; SBMNH 422343 [wet]. **MEXICO, Baja California Norte (Pacific Coast)** – multiple colonies; 8 miles SW of Isla Cedros, green, fine sand, coral, 28°00'00"N, 115°29'00"W (end), 115–118 m; coll. R/V ‘Velero III’, station 1254-41, 26 February 1941; SBMNH 422350 [wet]. –1 colony (with base, on flat, black rock); Isla Cedros, Cabo de San Augustin, SW of island, 8 miles, 243° T, dredge–fine sand, mud bottom, 28°01'02"N, 115°29'30"W (end), 109 m; coll. R/V ‘Velero IV’, station 1948-50, 27 April 1950; SBMNH 422341 [wet]. –fragment; off Islas San Benitos, S of islands, fine green sand, 28°12'45"N, 115°35'15"W (end), 129–173 m; coll. R/V ‘Velero III’, station 1010-39, 20 August 1939; SBMNH 422348 [wet]. –1 fragment; 5.5 miles S of Islas San Benitos, fine green sand, coarse grey sand, 28°13'55"N, 115°35'05"W (end), 120–147 m; coll. R/V ‘Velero III’, station 1251-41, 26 February 1941; SBMNH 422351 [wet]. –1 colony; outer coast, SE of Cabo San Quintin, 30°17'40"N, 115°54'40"W, 40–55 m, bottom composed of shale; coll. J McLean and P LaFollette, at ‘Searcher’ station 226-227 71-150, 17 October 1971; SBMNH 422414 [dry]. –multiple colony fragments; 8.21 miles, 324° T from Isla San Martin, dredge–sea stars, sand, 30°35'05"N, 116°11'49"W (end), 67 m; coll. R/V ‘Velero IV’, station 1692-49, 3 March 1949; SBMNH 422336 [wet]. –multiple fragments; 4 miles N of Todos Santos Island, shell, mud, gray sand, 31°53'20"N, 116°48'15"W, 75 m; coll. R/V ‘Velero III’, station 1245-41, 24 February 1941; SBMNH 422349 [wet].

**Other material examined. USA, California** – strands; San Diego County, La Jolla Canyon, N wall, Wheeler’s Bank, 46 m; coll. J Stewart, 19 December 1959; (in Limbaugh Collection, NMNH) [dry]. –strands; San Diego County, San Diego, Point Loma, 137–245 m; coll. R/V ‘Albatross’, 12 March 1904 (Nutting, 1912); USNM 030295 [wet]. –strands; San Diego County, San Diego, straight off Point La Jolla, 101–183 m; coll. Fager, with otter trawl 1, station-908, 28 January 1965; SIO/BIC CO 996. –strands; San Diego County, San Diego, from a station off point Loma, station SD-13; coll. MBL; no other data given [wet]. –strands; San Diego County, off La Jolla, 32°52'12"N, 117°17'24"W, 73.2-Van Veen grab (0.18m^2^), fine silty mud; coll. R/V ‘Oconostota’, F Rokop and S Luke, November 1969; SIO/BIC CO992. –strands; San Diego County, N La Jolla, 32°52'36"N, 117°17'48"W, 73 m, sand and shells; coll. unknown, Haul 1092, 19 June 1906; SIO/BIC CO997. –strands; San Diego County, off San Onofre, 91 m, M-15, OT-4, to 33°13'06"N, 117°29'36"W (end), with 25’ otter trawl; coll. Matsui and Burnett, R/V ‘Agassiz’, 29 March 1974; SIO-CO993, BI 74-4 [wet]. –strands; San Diego County, off San Onofre, 33°16'30"N, 117° 30'30"W, M-15, OT-5, 25’ otter trawl, 27 m; coll. T Matsui, R/V ‘Agassiz’, 29 March 1974; SIO/BIC CO994. –strands; San Diego County, off San Onofre, 33°16'00"N, 117°31'00"W, M-15, OT-6, 25’ otter trawl, 54 m; coll. T Matsui, R/V ‘Agassiz’, 29 March 1974; SIO/BIC CO995. –strands; San Diego County, off shore from Canyon de Las Encinas, 24 m; coll. C Turner, California Fish and Game, Terminal Island; 1 June 1967; (SIO-CO998) [wet]. –strands; Orange County, northern edge of San Gabriel Canyon/Newport Canyon, south of, and between, Huntington Beach and Newport Beach, trawl, ~33°34'20"N, 117°59'51"W, 60 m; coll. OCSD, station T-22, Haul 1, 28 February 2012 [wet]. –strands; Orange County, northern edge of San Gabriel Canyon/Newport Canyon, slightly eastward towards Newport Beach, Van Veen grab, ~33°34'22"N, 117°59'31"W, 59 m; coll. OCSD, station T-9, replicate 1, 15 July 2002 [wet]. –strands; Orange County, western edge of Newport Canyon, south of Newport Beach, trawl, ~33°34'51"N, 117°57'21"W, 55–60 m; coll. OCSD, station T-3, 22 February 2012 [wet]. –strands; Orange County, off Huntington Beach, trawl, ~33°35'57"N, 118°02'47"W, 36 m; coll. OCSD Survey 8612, station T-6, replicate 1, 1986 (vial with label “OCSD 0010”) [wet]. –strands; Orange County, off Huntington Beach, trawl, ~33°35'57"N, 118°02'47"W, 35 m; coll. OCSD, station T-6, Haul 1, 22 February 2012 [wet]. –strands; Los Angeles County, San Pedro, south of Point Fermin, northern edge of San Pedro escarpment, PV Trawl, T5 transect, ~33°41'07"N, 118°19'36"W, 137 m; coll. LACSD, station T5-137, 14 February 2012 [wet]. –fragment; Los Angeles County, (?)just south and west of San Pedro, PV Benthic, ~33°41'32"N, 118°20'14"W, 152 m; coll. LACSD, station E8 rock (8B?), JMB; Marine Biology Lab, City of Los Angeles), 16 July 1992 [wet]. –strands; Los Angeles County, off Torrance, Redondo Canyon, PV Trawl, T0 transect, ~33°48'34"N, 118°25'51"W, 61 m; coll. LACSD, station T0-61, 16 February 2012 [wet]. –strands; Los Angeles County, off Torrance, Redondo Canyon, PV Trawl, T0 transect, ~33°48'50"N, 118°26'22"W, 137 m; coll. LACSD, station T0-137, 16 February 2012 [wet]. –strands; Los Angeles County, Santa Monica, 33°52'30"N, 118°34'00"W, 25’ otter trawl, 73 m; coll. R/V ‘Agassiz’, T Matsui, M-15, OT-10, 29 March 1974; SIO/BIC CO1246 [wet].

Two specimens examined, SIO/BIC CO1856; SIO/BIC CO1859, had no collection data that could be found.

***Theseavariabilis* Studer, 1894**

**Material examined.** No apparent specimens of this species in SBMNH collection.

**Other material examined.** – 1 colony; USA, California, San Diego County, 400 m off shore of Scripps Institution, 50 m; coll. C Limbaugh, 23 July 1954; USNM 50633 [dry]. –single colonies in 3 lots; USA, California, San Diego County, La Jolla Canyon, South Wall, 43 m; coll. R Ghilardi and J Stewart, 02 January 1957; USNM 50634 [1 wet lot, 2 dry lots].

***Callogorgiakinoshitai* (Kükenthal, 1913)**

**Material examined.** 6 lots. **USA, Oregon** – Lane County, off the Oregon coast, 61.59 miles NW of light house and Sealion Cave, 44°02'11"N, 125°05'09"W, 1,400–1,600 m; coll. R/V ‘Yaquina’, Cruise 6710, 30 October 1967; SBMNH 422982 [wet]. **USA, Washington** – Clallam County, approximately 105 mi W of Cape Flattery, 48°36'30"N, 127°00'48"W, 2,189 m; coll. R Ruff, OSU, R/V ‘Yaquina’, Cruise DWD/BMT 9 = OSU BMT 558, 11 September 1971; SBMNH 422990 [wet]. –Clallam County, approximately 105 mi W of Cape Flattery, 48°36'30"N, 127°00'48"W, 1,998 m; coll. R Ruff, OSU, R/V ‘Yaquina’, Cruise DWD/BMT 10 = OSU BMT 559, 11 September 1971; SBMNH 422991 [wet].

**Other material examined.** – 1–2 fragments; USA, California, San Diego County, San Diego, Point Loma light-house, ~32°42'00"N, 117°14'00"W, (N. 82°30’ E. 5.9 miles), 220–240 m; coll. R/V ‘Albatross’, station 4356, 1904; SIO/BIC CO991 [wet]. –3 fragments; USA, California, San Diego County, San Diego, Point Loma light house, ~32°42'00"N, 117°14'00"W, no depth recorded; coll. ‘Albatross’, station 4359, 1904; SIO/BIC CO1808 [wet]. –several fragments; USA, California, San Diego County, off San Diego, off Point Loma light-house, 33°02'15"N, 120°36'30"W, 2,468 m; coll. ‘Albatross’, station 4391, 1904; SIO/BIC CO1809 [wet].

**Other material, not examined.** – Eastern S Pacific, South America, Zapallar, Chile, 350 m; 27 September 1977; USNM 75125 [wet]. –USA, California, San Diego County, San Diego, Point Loma, 245–283 m; 15 March 1904; USNM 30084 [wet]. –USA, California, San Diego County, San Diego Trough, ? station R-37, 32°26'02"N, 117°25'54"W, 1,194–1,097 m, by otter trawl, in mud; coll. F Rokop, R/V ‘Agassiz’, 21 April 1971; SIO/BIC CO1523. –USA, California, Monterey County, Pacific Grove, Monterey Bay, Point Piños, 36°38'16"N, 121°55'46"W, 1,381–1,752 m; 27 May 1904; USNM 49611 [wet]. –USA, California, Monterey County, Pacific Grove, Monterey Bay, Point Piños, 36°38'16"N, 121°55'46"W, 1,575–1,942 m; 31 May 1904; USNM 30030 [wet]. –USA, California, Monterey County, Pacific Grove, Monterey Bay, Point Piños, 36°38'16"N, 121°55'46"W, 1,575–1,942 m; 31 May 1904; USNM 43126 [wet]. –USA, California, Monterey County, Pacific Grove, Monterey Bay, Point Piños, 36°38'16"N, 121°55'46"W, 1,575–1,942 m; 31 May 1904; USNM 58986 [dry]. –USA, California, San Francisco Bay, 37°48'36"N, 122°14'15"W (label error: Monterey Bay), 1,609–1,476 m; 20 March 1975; USNM 75231 [wet].

–SIO/BIC CO946 is also identified as this species, but no locality data could be found.

***Parastenellapacifica* Cairns, 2007**

**Material examined.** 1 lot. **USA, Oregon** – numerous colonies/fragments; Oregon, Lane County, off Oregon coast, 58.14 mi NW of Sealion Cave, 44°21'53"N, 125°14'01"W, 2,086 m; coll. R/V ‘Acona’, OSU, Cruise 6408, haul OTB 41; SBMNH 422983 [wet].

***Parastenellaramosa* (Studer, 1894)**

**Material examined.** No apparent specimens of this species in SBMNH collection.

**Other material examined.** – 1 colony; N Pacific Ocean, USA, off Central California coast, Rodriquez Seamount, 34°02'26"N, 121°02'24"W, 735 m; 16 October 2003; USNM 1102453 [wet]. –2 colonies; N Pacific Ocean, USA, off Central California coast, Rodriquez Seamount, west of San Miguel Passage, 34°03'06"N, 121°03'00"W, 663 m; 13 October 2003; USNM 1027058 [wet]. –fragment; N Pacific Ocean, USA, off Central California, Rodriquez Seamount, ~34°03'11"N, 121°03'04"W, 664.6 m; coll. MBARI staff, PI, D Clague, 14 October 2003; MBARI T628-A12 [wet]. –1+ colony; USA, California, Monterey County, Monterey Bay, 36°27'09"N, 122°01'28"W, 950 m; coll. W Nybakken, 27 October 1988; MLML C0177 [wet].

***Plumarellalongispina* Kinoshita, 1908**

**Material examined.** ~33 lots. **USA, California** – fragment; San Diego County, W end of Cortes Bank, dredge–rocks, 32°33'24"N, 119°15'13"W (end), 82 m; coll. R/V ‘Velero IV’, station 1882-49, 26 August 1949; SBMNH 422399 [wet]. –fragments; San Diego County, W end of Cortes Bank, Snapper–Foraminifera sand, algae, 32°35'05"N, 119°18'52"W, no depth recorded; coll. R/V ‘Velero IV’, station 1876-49, 26 August 1949; SBMNH 422398 [wet]. –many fragments; San Diego County, 9.5 miles SW of Tanner Bank, 32°36'30"N, 119°20'00"W, loose rock, coralline, 131 m; coll. R/V ‘Velero III’, station 1346-41, 11 June 1941; SBMNH 422397 [wet]. –fragments; San Diego County, 9.5 miles NW of buoy, Cortes Bank, 32°33'15"N, 119°15'15"W, white sand, rock, 91 m; coll. R/V ‘Velero III’, station 1342-41, 10 June 1941; SBMNH 422395 [wet]. –1 colony, no base; Orange County, 33°32'47"N, 118°07'31"W, 216 m; coll. M Love, and party, with submersible ‘Delta’, 6 October 2005; with M Love’s number: A6649; SBMNH 422402 [wet, with small tip in 95% alcohol for sequencing]. –fragment; Los Angeles County, Santa Catalina Island, 7.25 miles SE of Seal Rocks, 33°14'25"N, 118°10'45"W, 276–364 m; coll. R/V ‘Velero III’, station 1430-41, 25 September 1941; SBMNH 422396 [wet]. –10 fragments (3 lots); Los Angeles County, Santa Catalina Island, 2.5 to 7.5 miles SE of Seal Rocks, 33°17'30"N, 118°15'55"W, approx. 164 m; coll. R/V ‘Velero III’, station 1429-41, subset station (?) D1, D2 or D3, 25 October 1941; SBMNH 422394 [dry; 1 wet].] . –2 colonies in 2 lots; Los Angeles County, Santa Catalina Island, off Avalon, 33°20'40"N, 118°19'31"W, 155–182 m; coll. R Fay, by trawl, 31 January 1974; SBMNH 45553 [wet]. –1 fragment; Los Angeles County, Santa Catalina Island, Avalon, 33°20'40"N, 118°19'31"W, taken alive with beam trawl off PBM boat; coll. unknown; legit. P Brophy, 31 January 1971; SBMNH 45555 [wet, with second label, data below (*)]. –multiple fragments; Los Angeles County, 15.42 miles, 248° T to Jewfish Point, Santa Catalina Island, 33°24'58"N, 118°01'00"W, 318 m; coll. R/V ‘Velero IV’, station 22790-75, 18 September 1975; SBMNH 422923 [wet]. –1 fragment; Los Angeles County, San Pedro Channel, 70 fathom Bank, 33°24'15"N, 118°01'15"W (end), rock, sponge, 156–235 m; coll. R/V ‘Velero III’, station 1212-40, 30 November 1940; SBMNH 422400 [wet]. –1 fragment; Los Angeles County, Santa Catalina Island, off Emerald Bay, in mud, 33°28'55"N, 118°30'05"W, 118–164 m; coll. R/V ‘Velero III’, Station 909-39, 29 January 1939; SBMNH 422393 [wet]. –1 colony; Los Angeles County, 4.5 miles, 113° T to Santa Barbara Island, N. Light, 33°30'55"N, 119°05'26"W (end), 255 m; coll. R/V ‘Velero IV’, station 24455-76, 7 March 1976; SBMNH 422922 [wet]. –1 colony; Los Angeles County, 6.7 miles, 330° T from N Light, Santa Barbara Island, dredge, tangles–2 large boulders and much small rock, rock bottom, 33°33'27"N, 119°04'00"W (end), 255 m; coll. R/V ‘Velero IV’, station 2062-51, 18 October 1951; SBMNH 422391 [wet]. –2 fragments; Los Angeles County, 10.4 miles, 351.5° T from N Light, Santa Barbara Island, dredge and tangles–mollusks, annelids, shrimps, *Lovenia*, brittle stars, mud bottom, 33°39'09"N, 119°03'00"W (end), 300 m; coll. R/V ‘Velero IV’, station 2061-51, 18 October 1951; SBMNH 422433 [dry]. – (*)1 fragment; Santa Barbara County, Santa Barbara, 34°23'28"N, 119°41'39"W, 109–164 m; coll. P Brophy, 26 April 1974; SBMNH 422925 [DH 420 = SBMNH-07, dry]. –multiple colony fragments; Santa Barbara County, 6.25 miles SE of South Point, Santa Rosa Island, rocky bottom with alcyonarians, 33°51'00"N, 120°00'20"W, 84 m; coll. R/V ‘Velero III’, 1291-41, 11 April 1941; SBMNH 422432 [dry]. –1 colony; Santa Barbara County, 1.3 miles, 154° T from San Pedro Point, Santa Cruz Island, dredge–*Ophiothrix*, few mollusks and sponges, bottom sand, 34°00'57"N, 119°30'10"W (end), 55 m; coll. R/V ‘Velero IV’, station 3021-55, 2 April 1955; SBMNH 422392 [wet]. –1 colony, Santa Barbara County, no base; Santa Barbara Channel, soft coral–snail and growth in separate container, 34°02'40"N, 119°18'57"W, 150 m; coll. M Love, 28 September 2004 (photo taken); SBMNH 422401[wet]. **MEXICO, Baja California Sur (Pacific Coast)** – 1 fragment; 9.5 miles W of Punta Malarrimo (just S of Isla Cedros), dredge–rocks, sand bottom, 27°49'00"N, 114°42'09"W (end), 16 m; coll. R/V ‘Velero IV’, station 2024-51, 18 April 1951; SBMNH 422431 [dry]. **MEXICO, Baja California Norte (Pacific Coast)** – multiple fragments; 25.4 miles, 181° T from Punta Banda Light, 31°19'00"N, 116°44'00"W (end), 264 m; coll. R/V ‘Velero IV’, station 10986-66, 19 February 1966; SBMNH 422924 [wet].

**Other material examined.** – 7+ fragments; N Pacific Ocean, Mexico, Baja California Norte, Cabo Colnett, 30°58'00"N, 116°22'00"W, 100–101 m; coll. D Brown and party, by midwater trawl 20, 24 November 1964; SIO/BIC CO1690 [wet]. –several fragments; Mexico, Baja California Norte, 40-mile Bank, 32°00'08"N, 117°56'16"W., 183 m; coll. by dredge; ‘EW Scripps’, 10 June 1938; Shepard No. 69, Access. No. BI 38-1, SIO/BIC CO403[wet]. –N Pacific Ocean, USA, California, San Diego County, San Diego, Point Loma, 32°41'57"N, 117°14'51"W, 179–402 m; 15 March 1904; USNM 25429 [wet]. –N Pacific Ocean, California, Channel Islands area, 33°45'51"N, 119°12'16"W, 250 m; 30 October 2002; USNM 1026795 [wet]. –N Pacific Ocean, California, Los Angeles County, Channel Islands, Santa Catalina Island, Gulf of Catalina, Dakins Cove (Avalon), ~33°20'47"N, 118°19'30"W, 146 m; 8 April 1897; USNM 49591 [dry]. –N Pacific Ocean, California, Los Angeles County, Channel Islands, Santa Catalina Island, Gulf of Catalina, Avalon, 155–183 m; 31 January 1974; USNM 73755 [wet]. –N Pacific Ocean, California, Monterey County, Monterey Bay, in Channel, 732 m; 9 July 1925; USNM 77285 [dry]. –N Pacific Ocean, California, Mendocino County, Point Arena, 38°57'45"N, 124°03'05"W, 437 m; 25 September 1890; USNM 49585 [wet]. –multiple colonies; North Eastern Pacific, off Oregon coast, Heceta Bank, hard bottom, 43°57'10"N, 124°50'35"W, 135 m; coll. A Valdés by ROV, RV ‘Ronald H. Brown’ (NOAA) and S/V ‘Ropos,’ Dive 615, 10 July 2001; LACoMNH Marine Biodiversity processing center number 99 [wet].

Specific location data for ~six lots collected by OCNMS during May 2006 collecting expedition was not accessible; report was published in July 2007 (Marine Sanctuary Conservation Series, NMSP-07-04). This species was found in the Survey Sites numbered 1, 11, 30 and 31, all located within the Olympic 2 Essential Fish Habitat (EFH) Conservation Area. As well, a specimen was collected by OCNMS in July 2008; data not available for publication at this time but is likely from the same area as the 2006 material. The 2008 material likely sent to NMNH, Smithsonian, for housing in the collection there.

***Primnoapacifica* Kinoshita, 1907**

**Material examined.** No apparent specimens of this species in SBMNH collection.

**Other material examined.** –1 colony; USA, southern coast of California, San Diego County, off La Jolla, near Soledad Hill, ~32°51'21"N, 117°18'24"W, 205–234 m; coll. R/V ‘Albatross’, 8 March 1904; USNM 57557 [wet]. –1 colony; USA, California, Monterey County, Monterey Bay, 36°21'46"N, 122°07'51"W, 1,609 m; coll. McDonald, Anderson, Antrim, 20 March 1975; MLML C0118 [wet]; specimen sent from same event to the Smithsonian, NMNH, in 1979.

***Narella* Gray, 1870**

**Material examined.** No apparent specimens from this genus in SBMNH collection.

***Acanella* Gray in Wright, 1869**

**Material examined.** No specimens of this genus in collection at SBMNH.

***Isidella* Gray, 1858**

**Material examined.** 2 lots. **USA (Oregon, Alaska)** – 3 fragments; USA, Oregon, Curry County, 29.79 miles W of Gold Beach, 44°24'17"N, 125°00'38"W, 1,244 m; coll. R/V ‘Yaquina’, Cruise Y7002 B, OSU, 19 February 1970; SBMNH 422981 [wet]. –1 complete (broken) colony; USA, Alaska, Gulf of Alaska, Welker Seamount, 55°01'05"N, 140°19'11"W, 1,049 m; coll. A Baco-Taylor, using submersible ‘Alvin’, dive 4035, sample 24, 11 August 2004; SBMNH 369349; [dry]. (This latter det. by P Etnoyer as *Isidellatentaculum*, new species, labeled as the **Paratype**).

***Keratoisis* Wright, 1869**

**Material examined**: ~3 lots. **USA, California** – multiple fragments; USA, California, Monterey County, Monterey Bay, 5.4–5.8 miles, 314° T from Point Piños Light to Mid Point, 36°43'00"N, 122°01'45"W, 436–809 m; coll. R/V ‘Velero IV’, stations 7462-61 or 7463-61, 10 October 1961; SBMNH 422980 [wet].

**Other material examined.** – 1 fragment; Eastern N Pacific, USA, California, Jasper Seamount, 30°21'54"N, 122°44'02"W, 1,375–1,910 m; coll. H Staudigel, by dredge, SEATOMADO Expedition, 2 November 1980; SIO CO2246 [wet]; this specimen could very likely be Keratoisis (Bathygorgia) profunda Wright. –fragments; Eastern N Pacific, USA, California, Fieberling Guyot, west of California Channel Islands, 32°23'25"N, 127°44'30"W, depth unknown; coll. unknown, 20 May 1991; USNM 93937 [wet].

***Lepidisis* Verrill, 1883**

**Material examined.** No specimens of this genus in collection of SBMNH.

**Other material examined.** – fragments; N Pacific Ocean, USA, California, Ventura/Los Angeles Counties, Channel Islands, ~40 miles SW of San Nicholas Island, 32°31'08"N, 119°42'10"W, 950 m; coll. J Ljubenkov, date collected not reported; USNM 59821 [wet].
